# Supplementary material for: Proportion of active tuberculosis among HIV-infected children after antiretroviral therapy in Ethiopia: A systematic review and meta-analysis
Source: PLOS Glob Public Health. 2024 Aug 2;4(8):e0003528. doi: 10.1371/journal.pgph.0003528 (PMC11296650; doi:10.1371/journal.pgph.0003528)
Supplement: S1 Text — (DOCX) [file pgph.0003528.s002.docx]

**PubMed searching strategy**

**(((((((((Tuberculosis) AND (incidence)) AND (HIV)) OR (Infected)) OR (positive)) OR (children)) OR (pediatrics)) AND (infant)) OR (Neonate)) AND (Ethiopia)**

((((("Tuberculosis"[All Fields] OR "tuberculosis"[MeSH Terms] OR "tuberculosis"[All Fields] OR "tuberculosis"[All Fields] OR "tuberculosis s"[All Fields]) AND ("epidemiology"[MeSH Subheading] OR "epidemiology"[All Fields] OR "incidence"[All Fields] OR "incidence"[MeSH Terms] OR "incidences"[All Fields] OR "incident"[All Fields] OR "incidents"[All Fields]) AND ("hiv"[MeSH Terms] OR "hiv"[All Fields])) OR ("infect"[All Fields] OR "infectability"[All Fields] OR "infectable"[All Fields] OR "infectant"[All Fields] OR "infectants"[All Fields] OR "infected"[All Fields] OR "infecteds"[All Fields] OR "infectibility"[All Fields] OR "infectible"[All Fields] OR "infecting"[All Fields] OR "infection s"[All Fields] OR "infections"[MeSH Terms] OR "infections"[All Fields] OR "infection"[All Fields] OR "infective"[All Fields] OR "infectiveness"[All Fields] OR "infectives"[All Fields] OR "infectivity’s"[All Fields] OR "infects"[All Fields] OR "pathogenicity"[MeSH Subheading] OR "pathogenicity"[All Fields] OR "infectivity"[All Fields]) OR ("positive"[All Fields] OR "positively"[All Fields] OR "positiveness"[All Fields] OR "positives"[All Fields] OR "positivity’s"[All Fields] OR "positivity"[All Fields]) OR ("child"[MeSH Terms] OR "child"[All Fields] OR "children"[All Fields] OR "child s"[All Fields] OR "children s"[All Fields] OR "children"[All Fields] OR "childs"[All Fields]) OR ("pediatrics"[All Fields] OR "pediatrics"[MeSH Terms] OR "pediatrics"[All Fields] OR "pediatric"[All Fields] OR "pediatric"[All Fields])) AND ("infant"[MeSH Terms] OR "infant"[All Fields] OR "infants"[All Fields] OR "infant s"[All Fields])) OR ("infant, newborn"[MeSH Terms] OR ("infant"[All Fields] AND "newborn"[All Fields]) OR "newborn infant"[All Fields] OR "neonatal"[All Fields] OR "neonate"[All Fields] OR "neonates"[All Fields] OR "neo natality"[All Fields] OR "neonates"[All Fields] OR "neonate s"[All Fields])) AND ("Ethiopia"[MeSH Terms] OR "Ethiopia"[All Fields] OR "Ethiopia s"[All Fields])

**Translations**

**Tuberculosis:** "tuberculosi"[All Fields] OR "tuberculosis"[MeSH Terms] OR "tuberculosis"[All Fields] OR "tuberculosis"[All Fields] OR "tuberculosis's"[All Fields]

**Incidence:** "epidemiology"[Subheading] OR "epidemiology"[All Fields] OR "incidence"[All Fields] OR "incidence"[MeSH Terms] OR "incidences"[All Fields] OR "incident"[All Fields] OR "incidents"[All Fields]

**HIV:** "hiv"[MeSH Terms] OR "hiv"[All Fields]

**Infected:** "infect"[All Fields] OR "infectability"[All Fields] OR "infectable"[All Fields] OR "infectant"[All Fields] OR "infectants"[All Fields] OR "infected"[All Fields] OR "infecteds"[All Fields] OR "infectibility"[All Fields] OR "infectible"[All Fields] OR "infecting"[All Fields] OR "infection's"[All Fields] OR "infections"[MeSH Terms] OR "infections"[All Fields] OR "infection"[All Fields] OR "infective"[All Fields] OR "infectiveness"[All Fields] OR "infectives"[All Fields] OR "infectivities"[All Fields] OR "infects"[All Fields] OR "pathogenicity"[Subheading] OR "pathogenicity"[All Fields] OR "infectivity"[All Fields]

**Positive:** "positive"[All Fields] OR "positively"[All Fields] OR "positiveness"[All Fields] OR "positives"[All Fields] OR "positivity"[All Fields] OR "positivity"[All Fields]

**Children:** "child"[MeSH Terms] OR "child"[All Fields] OR "children"[All Fields] OR "child's"[All Fields] OR "children's"[All Fields] OR "childrens"[All Fields] OR "childs"[All Fields]

**Pediatrics:** "paediatrics"[All Fields] OR "pediatrics"[MeSH Terms] OR "pediatrics"[All Fields] OR "paediatric"[All Fields] OR "pediatric"[All Fields]

**Infant:** "infant"[MeSH Terms] OR "infant"[All Fields] OR "infants"[All Fields] OR "infant's"[All Fields]

**Neonate:** "infant, newborn"[MeSH Terms] OR ("infant"[All Fields] AND "newborn"[All Fields]) OR "newborn infant"[All Fields] OR "neonatal"[All Fields] OR "neonate"[All Fields] OR "neonates"[All Fields] OR "neonatality"[All Fields] OR "neonatals"[All Fields] OR "neonate's"[All Fields]

**Ethiopia:** "Ethiopia"[MeSH Terms] OR "Ethiopia"[All Fields] OR "Ethiopia’s"[All Fields]
