# Supplementary material for: Proportion of active tuberculosis among HIV-infected children after antiretroviral therapy in Ethiopia: A systematic review and meta-analysis
Source: PLOS Glob Public Health. 2024 Aug 2;4(8):e0003528. doi: 10.1371/journal.pgph.0003528 (PMC11296650; doi:10.1371/journal.pgph.0003528)
Supplement: S1 Table — (DOCX) [file pgph.0003528.s003.docx]

Table S3: Quality appraisal result of included studies Ethiopia Using Joanna Briggs Institute (JBI) quality appraisal checklist

| **Author** | **Quality assessment questions** | | | | | | | | | | |  |  |  |
| --- | --- | --- | --- | --- | --- | --- | --- | --- | --- | --- | --- | --- | --- | --- |
|  | Q1 | Q2 | Q3 | Q4 | Q5 | Q6 | Q7 | Q8 | Q9 | Q10 | Q11 | Yes Total | Quality status | Overall appraisal |
| **Cohort studies** | | | | | | | | | | | | | | |
| **Muluget et.al (1)** | Y | Y | Y | UC | Y | Y | Y | Y | Y | Y | N | 10/11 | Low risk | Included |
| **Kebede et . al (2)** | Y | Y | Y | Y | UN | Y | Y | Y | Y | Y | Y | 10/11 | Low risk | Included |
| **Tiruneh et.al (3)** | Y | Y | Y | Y | Y | Y | Y | Y | Y | UN | N | 10/11 | Low risk | Included |
| **Kebede et.al (4)** | Y | Y | Y | Y | Y | Y | Y | Y | Y | Y | Y | 10/11 | Low risk | Included |
| **Tiruneh et.al (5)** | Y | Y | Y | Y | Y | Y | Y | Y | Y | Y | Y | 10/11 | Low risk | Included |
| **Endalamaw et.al(6)** | Y | Y | Y | Y | Y | Y | Y | Y | Y | Y | N | 10/11 | Low risk | Included |
| **Tessu et.al (7)** | Y | Y | Y | Y | Y | Y | Y | Y | Y | Y | Y | 10/11 | Low risk | Included |
| **Gebeyehu et.al (8)** | Y | Y | Y | Y | Y | Y | Y | Y | Y | Y | Y | 10/11 | Low risk | Included |
| **Wubale et.al (9)** | Y | Y | Y | Y | UN | Y | Y | Y | Y | Y | Y | 10/11 | Low risk | Included |
| **Sissay et.al (10)** | Y | Y | Y | Y | UN | Y | Y | Y | Y | UN | Y | 9/11 | Low risk | Included |
| **Mequanente et.al (11)** | Y | Y | Y | Y | UN | Y | Y | Y | Y | UN | Y | 9/11 | Low risk | Included |
| **Endalk et.al (12)** | Y | Y | Y | UN | Y | UN | Y |  | Y |  | Y | 8/11 | Low risk | Included |
| **Westerlund et.al (13)** | UN | Y | Y | UN | UN | Y | Y | Y | Y | UN | Y | 7/11 | Low risk | Moderate |

Key: Y=yes, N=no, UC=unclear, Q=Question

**References**

1. Alemu YM, Andargie G, Gebeye E. High Incidence of Tuberculosis in the Absence of Isoniazid and Cotrimoxazole Preventive Therapy in Children Living with HIV in Northern Ethiopia: A Retrospective Follow-Up Study. PloS one. 2016;11(4):e0152941.

2. Kebede F, Kebede T, Kebede B, Abate A, Jara D, Negese B, et al. Time to Develop and Predictors for Incidence of Tuberculosis among Children Receiving Antiretroviral Therapy. Tuberculosis research and treatment. 2021;2021:6686019.

3. Deyas FTY. Effect of highly active antiretroviral treatment on TB incidence among HIV infected children and their clinical profile, retrospective cohort study, Sout West Ethiopia. Scientific Reports 10:21468 https://doiorg/101038/s41598-020-78466-0. 2020.

4. Kebede F, Tarekegn H, Molla M, Jara D, Abate A. Incidence and Predictors of Pulmonary Tuberculosis among Children Who Received Antiretroviral Therapy (ART), Northwest Ethiopia: A Multicenter Historical Cohorts Study 2009-2019. Journal of tropical medicine. 2022;2022:9925693.

5. Firew Tiruneh* YDaDA. tuberculosis-incidence-among-hiv-infected-children-on-haart-and-their-clinical-profile-retrospective-cohort-study-south-. Journal of AIDS & Clinical Research Research Article Volume 11:3,2020 DOI: 1037421/jar202011808. 2020.

6. Aklilu Endalamaw* EHEaNT. Incidence of tuberculosis in children on antiretroviral therapy: a retrospective cohort study. BMC Res Notes 11:745 https://doiorg/101186/s13104-018-3846-z. 2018.

7. Masino Tessu Beshir1 Aklil Hailu Beyene2 Kenean Getaneh Tlaye3 TMD. Incidence and predictors of tuberculosis among HIV- positive children at Adama Referral Hospital and Medical College, Oromia, Ethiopia: a retrospective follow-up study. epihorg 2017; 41, Article ID: e2019028, 8 ( https://doi.org/10.4178/epih.e2019028).

8. Ayalaw SG, Alene KA, Adane AA. Incidence and Predictors of Tuberculosis among HIV Positive Children at University of Gondar Referral Hospital, Northwest Ethiopia: A Retrospective Follow-Up Study. International scholarly research notices. 2015;2015:307810.

9. Melkamu MW, Gebeyehu MT, Afenigus AD, Hibstie YT, Temesgen B, Petrucka P, et al. Incidence of common opportunistic infections among HIV-infected children on ART at Debre Markos referral hospital, Northwest Ethiopia: a retrospective cohort study. BMC infectious diseases. 2020;20(1):50.

10. Chanie ES, Bayih WA, Birhan BM, Belay DM, Asmare G, Tiruneh T, et al. Incidence of advanced opportunistic infection and its predictors among HIV infected children at Debre Tabor referral Hospital and University of Gondar Compressive specialized hospitals, Northwest Ethiopia, 2020: A multicenter retrospective follow-up study. Heliyon. 2021;7(4):e06745.

11. Mequanente DA, Srinivasan P, Mallika G, Ansari PMT, Wale M. Incidence of Opportunistic Infections among HIV-infected Children on ART at Gondar University Specialized Hospital, Ethiopia. Indian Journal Of Science And Technology. 2022;15(34):1675-82.

12. Endalk B ESC, Fisha A.G, Gebeyaw Biset et.al Incidence and predictors of tuberculosis among children on antiretroviral therapy at northeast Ethiopia comprehensive specialized hospitals, 2022; A multicenter retrospective follow-up study>. Heliyon 8 (2022) e12001. 2022;https://doi.org/10.1016/j.heliyon.2022.e12001.

13. Emil Westerlund DJ, Zewdie Mulissa Inger Hallström and Bernt Lindtjørn5. Pre-ART retention in care and prevalence of tuberculosis among HIV-infected children at a district hospital in southern Ethiopia. BMC pediatrics. 2014;14:250 <http://www.biomedcentral.com/1471-2431/14/250>.
