# Supplementary material for: Proportion of active tuberculosis among HIV-infected children after antiretroviral therapy in Ethiopia: A systematic review and meta-analysis
Source: PLOS Glob Public Health. 2024 Aug 2;4(8):e0003528. doi: 10.1371/journal.pgph.0003528 (PMC11296650; doi:10.1371/journal.pgph.0003528)
Supplement: Attachment — Submitted filename: PGPH-D-23-02361_R1_Reviewed NAD.pdf [file pgph.0003528.s006.pdf]

PLOS Global Public Health

Meta-Analysis of Active Tuberculosis Occurrence among Children Living with HIV Post Anti-Retroviral Therapy Initiated in Ethiopia

--Manuscript Draft--

**Manuscript Number:**	PGPH-D-23-02361R1
**Article Type:**	Research Article
**Full Title:**	Meta-Analysis of Active Tuberculosis Occurrence among Children Living with HIV Post Anti-Retroviral Therapy Initiated in Ethiopia
**Short Title:**	Meta-Analysis of Active Tuberculosis Occurrence among Children Living with
**Corresponding Author:**	Fassikaw Kebede, BSC, MPH<br>Woldia University<br>Woldia, Amhara ETHIOPIA
**Order of Authors:**	Fassikaw Kebede Bizuneh, BSc, MPH , PHD Felloe
	Dejen Tsegaye
	Tsehay Kebede
	Belete Negese
**Keywords:**	HIV; children; Ethiopia; Prevalence; Tuberculosis
**Abstract:**	Abstract<br>Background: Despite the effectiveness of antiretroviral treatment in reducing morbidity and mortality from opportunistic infections among children living with HIV (CLHIV), tuberculosis (TB) remains a significant cause of mortality, accounting for one in every three deaths. However, in Ethiopia, there is a lack of aggregated data on the pooled prevalence of TB and HIV co-infection for children living with HIV. Therefore, this meta-analysis aims to estimate the prevalence of active TB in HIV-infected children nationwide after initiating ART between December 30, 2012, and January 1, 2023.<br>Methods; A comprehensive review and analysis were conducted from December 30, 2012, to January 1, 2023. A total of 1235 articles were extracted from five databases. The study followed PRISMA guidelines. Pooled estimates were obtained using a weighted inverse variance random-effects model in STATA version 17. Heterogeneity was evaluated using Cochran's Q test and I2 statistic. Subgroup analysis, publication bias, and sensitivity analysis were also performed. This systemic review nd met-analysis report is registered in Prospero with CRD42024502038<br>Results; In the final meta-analysis, 13 individual studies were included. The mean age ~~of participants' children~~ was 8.2 (±3.6) years. During the screening of active TB among 5,834 ~~HIV infected participants~~ children, 834 cases of TB with HIV co-infection were reported. The overall prevalence of tuberculosis was 12.07% (95%CI: 10.71-13.41; I2=63.4%, P=0.001). In the final subgroup analysis, TB prevalence was higher in health centers (14.1%, 95% CI: 11.74-16.33) compared to hospitals (11.05%, 95% CI: 9.4-12.3). Similarly, in terms of study regions, Oromia had the highest TB prevalence at 15.6% (95% CI: 10.2-20.6), followed by southern nation nationality regions (SNNR) at 12.8% (95% CI: 10.03-15.67). In the final random-effects meta-regression, advanced WHO clinical stages III and IV (AOR = 2.27, 95% CI: 1.81-2.83), missed cotrimoxazole preventive therapy (CPT) (AOR = 3.26, 95% CI: 1.57-5.28), baseline hemoglobin levels ≤10mg/dl (AOR = 4.26, 95% CI: 3.47-5.24, I2=43.3, P=0.001), and missed isoniazid preventive therapy (IPT) (AOR = 2.28, 95% CI: 1.99-3.08) were predictive factors for the occurrence of active TB.<br>Conclusion: More than one in ten HIV-infected children in Ethiopia develop active TB. Risk factors include advanced WHO clinical stages IV and III, as well as low hemoglobin levels. Effective management involves concurrent and continuous use of Cotrimoxazole preventive therapy (CPT), Isoniazid preventive therapy (IPT), and antiretroviral therapy (ART) for children living with HIV.
**Additional Information:**	
**Question**	**Response**

Powered by Editorial Manager® and ProduXion Manager® from Aries Systems Corporation

**Financial Disclosure**<br><br>Enter a financial disclosure statement that describes the sources of funding for the work included in this submission and the role the funder(s) played. This includes grants and any commercial funding of the work or authors.<br><br>This statement will be typeset if the manuscript is accepted for publication.<br><br>*Please review the submission guidelines and the instructions link below for detailed requirements and guidance.*	The authors received no specific funding for this work.
**Competing Interests**<br><br>On behalf of all authors, disclose any competing interests that could be perceived to bias this work.<br><br>This statement will be typeset if the manuscript is accepted for publication.<br><br>*Please review the instructions link below and PLOS Global Public Health's competing interests policy to determine what information must be disclosed at submission.*	The authors have declared that no competing interests exist.
**Data Availability**<br><br>Before publication, Authors are required to make fully available and without restriction all data underlying their findings. Please see our PLOS Data Policy page for detailed information on this policy.<br><br>A **Data Availability Statement**, detailing where the data can be accessed, is required at first submission. Insert your	All relevant data are within the paper and its Supporting Information files

Data Availability Statement in the box below.<br><br>Please see the data reporting section of our submission guidelines for instructions on what you need to include in your Data Availability Statement.<br><br>This statement will be typeset if the manuscript is accepted for publication.<br><br>*PLOS allows rare exemptions to address legal and ethical concerns. If you have legal or ethical restrictions, please use the box below to detail these in full sentences for the Journal team to consider.*	

Powered by Editorial Manager® and ProduXion Manager® from Aries Systems Corporation

Manuscript
Click here to access/download;Manuscript;Clean Manuscript 2122.docx

Meta-Analysis of Active Tuberculosis Occurrence among Children Living with HIV Post Anti-Retroviral Therapy Initiated in Ethiopia

Fassikaw Kebede[1*], Dejen Tsegaye[2], Tsehay Kebede[3], Belete Negese[4] ,

[1]College of Health Sciences, Woldia University, Woldia, Ethiopia

[2] College of Health Sciences, Debre Markos University, Ethiopia

[3] College of Social Science Bahir Dare University, Bahir Dare, Ethiopia

[4] College of Health Sciences, Debre Berhan University, Ethiopia

Corresponding Author (FK) =

= fassikaw123@gmail.com

List of Emails

1. fassikaw123@gmail.com
2. dejenetsegaye8@gmail.com
3. yabebalij@gmail.com
4. tsehaynolawit@gmail.com
5. tesfayeshumet@gmail.com

Abstract

Background: Despite the effectiveness of antiretroviral treatment in reducing morbidity and mortality from opportunistic infections among children living with HIV (CLHIV), tuberculosis (TB) remains a significant cause of mortality, accounting for one in every three deaths. However, in Ethiopia, there is a lack of aggregated data on the pooled prevalence of TB and HIV co-infection for children living with HIV. Therefore, this meta-analysis aims to estimate the prevalence of active TB in HIV-infected children nationwide after initiating ART between December 30, 2012, and January 1, 2023.

Methods; A comprehensive review and analysis were conducted from December 30, 2012, to January 1, 2023. A total of 1235 articles were extracted from five databases. The study followed PRISMA guidelines. Pooled estimates were obtained using a weighted inverse variance random-effects model in STATA version 17. Heterogeneity was evaluated using Cochran's Q test and I2 statistic. Subgroup analysis, publication bias, and sensitivity analysis were also performed. This systemic review nd met-analysis report is registered in Prospero with CRD42024502038

Results; In the final meta-analysis, 13 individual studies were included. The mean age of participants' children was 8.2 (±3.6) years. During the screening of active TB among 5,834 HIV-infected participants children, 834 cases of TB with HIV co-infection were reported. The overall prevalence of tuberculosis was 12.07% (95%CI: 10.71-13.41; I2=63.4%, P=0.001). In the final subgroup analysis, TB prevalence was higher in health centers (14.1%, 95% CI: 11.74-16.33) compared to hospitals (11.05%, 95% CI: 9.4-12.3). Similarly, in terms of study regions, Oromia had the highest TB prevalence at 15.6% (95% CI: 10.2-20.6), followed by southern nation nationality regions (SNNR) at 12.8% (95% CI: 10.03-15.67). In the final random-effects meta-regression, advanced WHO clinical stages III and IV (AOR = 2.27, 95% CI: 1.81-2.83), missed cotrimoxazole preventive therapy (CPT) (AOR = 3.26, 95% CI: 1.57-5.28), baseline hemoglobin levels ≤10mg/dl (AOR = 4.26, 95% CI: 3.47-5.24, I2=43.3, P=0.001), and missed isoniazid preventive therapy (IPT) (AOR = 2.28, 95% CI: 1.99-3.08) were predictive factors for the occurrence of active TB.

Conclusion: More than one in ten HIV-infected children in Ethiopia develop active TB. Risk factors include advanced WHO clinical stages IV and III, as well as low hemoglobin levels. Effective management involves concurrent and continuous use of Cotrimoxazole preventive

therapy (CPT), Isoniazid preventive therapy (IPT), and antiretroviral therapy (ART) for children living with HIV.

Prospero **registration (CRD42024502038)** https://www.crd.york.ac.uk/prospero/#recordDetails

Keywords: HIV, Children, prevalence, Tuberculosis, Ethiopia

Introduction

People living with Human Immune deficiency virus (PLHIV) are more susceptible to tuberculosis (TB), which is a leading cause of mortality[1, 2]. There is a strong synergy between HIV infection and TB, while PLHIV are at high risk of dying from TB and HIV infection is the biggest risk factor for active TB incidence through target reduction of CD4 count and cellular immuni[3, 4]. The waning of the immune system increases the endogenous reactivation of dormant TB bacilli in the lung[5]. Despite breakthroughs in diagnosis and the widespread availability of chemotherapy, TB-related mortality in children accounts for 40% of global TB deaths, 25% of in-hospital deaths, and 18% of inpatient hospitalizations [6-9].

Globally in 2020, there were 1.2 million TB deaths from HIV-negative people, and 208,000 in HIV-positive individuals[10], and remains the leading cause of morbidity and mortality for PLHIV worldwide [11]. In Sub-Saharan Africa (SSA) countries, TB-associated new incidence of cases and new mortality were reported as 2,017 cases/100,000 patients per year [12] and 25 cases /100 patients per year, respectively [6, 7, 13]. According to the 2022 Global TB report for African continents, accounted for 1.5 million TB deaths, of which 14.30% of cases were co-infected with HIV [1]. In Sub-Saharan African countries, 10%–15% of the population suffered from the twine epidemic [14, 15]. The risk of developing TB disease is 21 times higher for people living with HIV and 51% lifetime risk of TB than those without HIV patients (1, 2).

Previous systematic review findings indicated TB is associated with 37% of deaths for PLHIV [11, 16] and 51% lifetime risk [1, 2]. Similarly, previous primary studies' findings reported twine infection of TB and HIV[12, 13, 17-21], having body mass index (BMI ≤18.5 kg/m2), missing CPT, poor ART adherence, and history of alcohol use were predictors for TB-associated death. Particularly declining CD4 count were a proxy indicator for premature death during co-infection treatment.

In Ethiopia in 2016, an estimated 710,000 people were living with HIV, and 62,000 of them were children living with HIV [22, 23]. Extra-pulmonary Tuberculosis (EPTB) is the most common kind of tuberculosis (TB) diagnosed and treated in seropositive children, and TB is responsible for one out of every four deaths in resource-limited settings [24, 25]. According to national profiles of TB and HIV patients, 11% of TB cases had HIV, while 9.1% of HIV patients had active TB [26-28]. Several small-scale studies in Ethiopia found TB incidence ranging from 7.2% in Amhara [29] to 23.6% in the south [30] regions. Despite concomitant administration of ART with IPT demoting> 80% of active TB[5], due to their age and immune-suppressing of HIV, children experienced active TB incidence [31].

In Ethiopia, deaths with those twine epidemics of TB and HIV are varied across each region with 23.01 cases per 100 person-years in Tigray [32] for adults and 17.15 cases per 100 years for children in the SNNR [33]. Children living with HIV face disproportionate loss of life from the co-infections since their young age category and immature immunity make them exposed to premature death [4, 25]. By administering IPT concurrently with HAART, after ruling out active TB symptoms, the incidence of new TB cases in children living with HI was reduced by over 90%[3, 34, 35]. Despite this evidence of HAART for reducing TB-associated morbidity for seropositive (children living with HIV), however, there is a lack of comprehensive and aggregated data during the co-infection disease courses of TB and HIV infection in Ethiopia

Objectives

- Determining pooled Tuberculosis-incidence in HIV-positive children in Ethiopia
- Identifying Predictors for TB Incidence among Children Living with HIV in Ethiopia

Data Searching Strategy

We conducted a systematic review of published and unpublished articles from multiple databases, including PubMed, HINARY, WEB OF SCIENCE, Africa Journals Online, and Google Scholar. The search focused on English language articles published between December 30, 2012, and January 1, 2023. We employed controlled vocabulary terms (MeSH) and free text to extract relevant articles. The search ~~encompassed various databases such as PubMed, MEDLINE, HINARY, Africa Journals Online, and Google Scholar, and~~ included topics such as

active tuberculosis, pulmonary TB, extrapulmonary TB, HIV infection, individuals, children, pediatrics, neonates, lymphadenitis, disseminated TB, and Ethiopia. The search terms used to identify relevant studies included "Epidemiology" OR "Incidence" OR "Case fatality" "Tuberculosis" OR "Pulmonary Tuberculosis" OR "Disseminated Tuberculosis" OR "Lymphadenitis" AND "HIV" OR "AIDS" AND "Children" OR "Pediatrics" OR "Infant" AND "Ethiopia". We considered articles published between December 30, 2012, and January 1, 2023, and utilized cross-sectional, retrospective, and prospective cohort study designs in the search process. The selection criteria were predefined to identify the most relevant studies for the review.

Inclusion criteria:

We included scientific papers that reported the following criteria for the final analysis

1. We included studies that reported TB and HIV co-infections in HIV-positive children in Ethiopia
2. We only considered cross-sectional and/or cohort studies as observational studies.
3. Articles containing prevalence or incidence reports of TB in HIV-positive children
4. A scientific paper published before January 1st, 2023with the study subjects only on children ≤15 years

Exclusion criteria

Articles without a journal name and /or author, conference proceedings, presentations, and reviews were excluded from the final meta-analysis.

Outcome ascertainment

The outcome variable for this meta-analysis report was the TB occurrence among HIV-positive children on Anti-retroviral therapy.

Quality Assessment and Appraisal Procedures

Four Authors (FK, BN, DT, and TK) independently extracted the data and evaluated the quality of each study by determining the eligibility of the titles and abstracts of the studies after removing duplicates. The discussion was used to settle any disagreement or uncertainty that arose during the article extraction process. These reviewers assessed the full-text articles; if one or more of them believed an article could be significant, it qualified after the article was carefully examined for its titles, abstracts, and full text among authors (FK, TK, DT, and BK) used a

Microsoft Excel spreadsheet to extract the specifics of each article. During data extraction using the critical appraisal process using Preferred Reporting Items for Systematic Reviews and Meta-Analysis (PRISMA-2020)[36] **(S1= PRISMA Checklist 2020).**

Three independent reviewers assessed each included article's quality using the Newcastle-Ottawa Scale standards (good, fair, and poor) given for all articles **[37, 38].** All eligible studies were approved by all author's agreements about principal investigators, year of publication, study period, study setting, study population, and sample size retrieved from the identified articles. In each of the included studies, the risk of bias was assessed by all listed authors (FK, and TK), evaluated, and screened. The Joanna Briggs Institute of Critical Appraisal (JBI) checklist was used to evaluate the papers' quality, and the results were incorporated into the final meta-analysis (**S2).** Any disagreements among reviewers regarding the critical appraisal were settled through discussion and consensus-building

Data Synthesis and Analysis Procedures

Using End-Note Aversion 8.1, all detected and potentially suitable published article citations were exported and gathered; duplications were eliminated during the selection and screening processes. Two independent reviewers (FK, and TK) first reviewed the abstracts of the publications before moving on to the full-text articles, which they then evaluated following the particular standards for ultimate inclusion and modifying the data on a Microsoft Excel spreadsheet, we employed the STATA version 17 for additional analysis. Descriptive statistics, fixed effects, and random-effect model regression were used to present the review's results[39] to estimate TB-associated death of PLHIV post-antiretroviral therapy in Ethiopia[39]. The eligible published data files were extracted using Meta-XL Excel version 5.3[40] sheet and exported to STATA version 17 to estimate the pooled TB-associated mortality of people living with HIV/AIDS in Ethiopia. The estimated risk factors from each study were combined and determined as a single estimation using a random effect regression model in the final meta-analysis[39]. The Higgs I^2 statistics were also utilized to detect heterogeneity. Heterogeneity between studies was elaborated using Cochran's Q test and quantified with the I^2 statistics[41]. The degrees of statistical heterogeneity between the studies were assessed using I2 statistics; values of 25%, 50%, and 75% were thought to indicate modest, medium, and high levels of heterogeneity, respectively [38]. The source heterogeneity among the included studies was

further examined using the subgroup and sensitivity analyses[39]. For further clarification on the source of heterogeneity, the random effect meta-regression was reported on the study setting, regions, and study population done by subgroup analysis. In the final meta-analysis review, the estimated risk factors obtained from each study were pooled and determined as a single estimate with its corresponding 95% confidence interval. The random effect regression model was used for the data-identified heterogeneous analysis [39].

Publication of Biases and Sensitivity Analysis

The publication biases were assessed by visual inspection of funnel plots of the graph and quantitative using Egger's weighted regression at p <0.1[42, 43]. In addition, we performed a leave-one-out sensitivity analysis to confirm a study with a biased direction of pooled estimates of Begs and Eggers tests[39]. The publication biases were assessed using inspection of funnel plots of the shape of the graph, quantitatively using Egger's weighted regression test of the P-value <0.1[42, 43], and using a leave-one-out sensitivity analysis to confirm that there were no studies potentially biased direction using Begs and Eggers test[39].

Result

Eligible studies screening

A total of 1221 primary studies were identified, including 43 from Web of Science, 631 from PubMed, 352 from Medline, 15 from Scopus, and 162 articles from Google Scholar. After removing duplicate articles and screening titles and abstracts, 1208 articles were excluded. Finally, 13 articles met the inclusion criteria and were included in the analysis **(Figure 1).**

Figure 1: A PRISMA flow diagram for describing the flow of the chart of selected articles

Study characteristics

Thirteen (N=13) individual studies were included final analysis [4, 25, 29, 30, 44-52]. Seven of those articles were from Amhara [29, 44-46, 51, 52], three were from SNNR [30, 47, 49], two were from Benishangul Gumuz [4, 25], and one from Oromia[50] regions respectively. A total sample size of 5834 participants' children was included in the final analysis. The mean (±SD age of the participants was estimated at 8.2(±3.6) years. More than half (N=9/13, 69.2%) of studies

recruited cohort design [25, 30, 45, 47, 49, 51, 52], and 38.8% of the included [4, 29, 44, 48, 51] papers used ≥ 10 years of follow-up (**Tabel 1).**

Table 1: Characteristics of included articles/studies reporting the prevalence of TB in HIV-positive children in Ethiopia after ART initiation in Ethiopia.

Descriptive of reports of included studies

The majority (7/13= 53.8%) [4, 25, 30, 44-46, 50] of studies reported TB magnitudes, whereas the remaining four [29, 47, 51, 52] included crude incidence. The highest (23.6%) and the lowest (7.2%) TB burden were reported in the southern nations (SNNR) [30] and Amhara [29] regions, respectively. The final meta-analysis report indicated the pooled active TB prevalence among children after ART was estimated at 12.1% (95% CI: 10.7-13.4; I2 = 63.4%, p=0.001) **(Figure 2).**

Figure 2: Forest plot of the pooled prevalence of active TB I HIV-infected children

Sub-group-analysis using selected themes

In our subgroup analysis, we examined TB prevalence among HIV-positive children based on study regions and settings. Among studies conducted in hospital setups, the TB prevalence was slightly lower at 11.05% (95%CI: 9.4-12.3) compared to facility-based studies with a prevalence of 14.1% (95%CI: 11.74-16.33) **(Figure 3).**

Figure 3: Forest plot of subgroup analysis by study setting for Active TB in HIV-infected children

We also conducted a subgroup analysis based on the study regions. The pooled TB prevalence among HIV-positive children after ART initiation was significantly higher in studies conducted in the Oromia region at 15.6% (95%CI: 10.2-20.6) compared to studies conducted in the SNNPR region with a prevalence of 12.8% (95%CI: 10.03-15.67) **(Figure 4).**

Figure 4: Forest plot for subgroup analysis by follow-up time of active TB in HIV-infected children

Similarly, we conducted a subgroup analysis based on different follow-up periods for the sampled population. The pooled TB prevalence among HIV-positive children after ART

initiation with a follow-up period of ≤10 years was significantly higher at 13.67% (95%CI: 11.24 --15.1) compared to a longer follow-up period of >10 years, which had a prevalence of 10.9% (95%CI: 9.1-12.8).

Associated Risk factors for active TB

n this systematic review, we analyzed adjusted odds ratios from primary studies to identify risk factors for the occurrence of active tuberculosis (TB) among HIV-infected children. ~~The themes of missed isoniazid preventive therapy (IPT), missed cotrimoxazole preventive therapy (CPT), low hemoglobin levels (≤10mg/dl), and advanced WHO clinical stages (III&IV) were found to be predictors for the occurrence of active TB in HIV-infected children. Accordingly,~~ studies demonstrated that advanced WHO clinical stages III and IV (AOR = 2.27, 95% CI: 1.81-2.83), missed cotrimoxazole preventive therapy (CPT) (AOR = 3.26, 95% CI: 1.57-5.28), baseline hemoglobin levels ≤10mg/dl (AOR = 4.26, 95% CI: 3.47-5.24, I2=43.3, P=0.001), and missed isoniazid preventive therapy (IPT) (AOR = 2.28, 95% CI: 1.99-3.08) were predictive factors for the occurrence of active TB.

Effects of missed IPT on TB occurrence for children living with HIV

We found four published articles that examined the relationship between missed isoniazid preventive therapy (IPT) and the occurrence of active TB. The final meta-regression analysis showed that missed IPT increased the risk of active TB by 2.28 times compared to the control groups (AOR = 2.28, 95% CI: 1.99-3.08) (I2 = 82.8%, P = 0.03) **(Figure 5).**

Figure 5. Forest ~~plotted~~ for impact of missed IPT among active TB in HIV-infected children.

Effects of missed CPT on TB incidence

We identified four published articles that investigated the association between missed cotrimoxazole preventive therapy (CPT) and the occurrence of active tuberculosis (TB). The final meta-regression analysis revealed that the odds of developing active TB were 3.26 times higher in HIV-infected children who missed cotrimoxazole preventive therapy (AOR = 3.26, 95% CI: 1.57-5.28) (**Figure 6).**

Figure 6. Forest plotted for the impact of missed CPT among active TB in HIV-infected children.

Impacts of advanced WHO clinical stages III &IV on TB occurrence

Accordingly, in a meta-analysis of four studies, the odds of tuberculosis occurrence among HIV-infected children in WHO clinical stages III and IV were 2.27 times higher compared to those in WHO clinical stages I&II (AOR = 2.27, 95% CI: 1.81-2.83).

Impacts of declining haemoglobin level on TB occurrence

HIV-positive children with baseline hemoglobin levels ≤10mg/dl had 4.26 times higher odds of developing tuberculosis (TB) compared to those with baseline hemoglobin levels >10mg/dl. This conclusion is based on data from six studies involving 2,371 participants (AOR = 4.26, 95% CI: 3.47-5.24, I2=43.3, P=0.001).

Random effect Meta-Regression

In the final report of this meta-analysis, a random-effects meta-regression analysis was conducted, considering covariates such as sample size and follow-up period. The analysis revealed that neither the sample size (P= 0.14) nor the follow-up periods (P= 0.42) significantly influenced heterogeneity, as shown in the analysis **(Tables 2).**

Sensitivity Analysis

The sensitivity analysis showed that all studies were within the confidence interval bounds of the meta-regression. Egger's (P=0.01) and Begg's tests (P=0.01) indicated no significant publication biases.

Publication bias assessment

The publication bias was checked based on the symmetry of the funnel plots and quantitatively using Begg's and Egger's tests. We carried out Egger's regression and the report indicated the absence of publication bias with P=0.064**(Figure 7).**

Figure 7: Funnel plot for active TB for HIV-infected children

Discussion

In this meta-analysis, 13 individual studies with a total of 5834 participants were included. Among them, 834 HIV-positive children were found to have active tuberculosis (TB). The pooled prevalence of active TB in HIV-co-infected children, estimated during the meta-analysis, was 12.07% (95%CI: 10.73 - 13.4, I2 = 63.4%; P = 0.001). This finding is higher than previously reported in 0.78% in Ethiopia[53],43% in SSA countries [54], and 1.03 % in Portugal[55]. It is lower than the previous meta-analysis reported 15% in middle-income countries [56, 57]. The variation in pooled TB prevalence in Ethiopia at the national level may be attributed to differences in study time and variations among the included studies[58]. Conversely, the finding of this finding at 2016 , which is 32% in Nigeria [59]. The lower TB prevalence in this Ethiopian meta-analysis compared to the 2016 study in Nigeria can be attributed to differences in study populations, settings, TB control measures, and various factors such as study methodology, sample size, healthcare infrastructure, access to diagnostics, treatment practices, and regional differences. The Ethiopian health ministry's efforts to improve TB control are likely a contributing factor. It is important to consider specific context and population characteristics when interpreting and comparing prevalence findings.

In this meta-analysis report, there is high heterogeneity among studies was observed and, a subgroup analysis was conducted by study regions and follow-up periods, revealing a significant variation in the pooled TB prevalence among HIV-positive children. The active TB prevalence in the Oromia region was estimated at 15.6% (95%CI: 10.2-20.6), compared to 12.8% (95%CI: 10.03-15.67) in the SNNR region. Additionally, the pooled TB prevalence with ≤10 years of follow-up period was higher at 13.67% (95%CI: 11.24-15.1) compared to studies with >10 years of follow-up, which had a prevalence of 10.9% (95%CI: 9.1-12.8). The possible reason for the variations in pooled TB prevalence can be influenced by factors such as population density, socioeconomic conditions, healthcare infrastructure, access to healthcare services, transmission dynamics, co-infections, diagnostic practices, and duration of follow-up[58].

The risk of active TB occurrence among HIV-infected individuals was determined using predictors through a random-effects meta-regression analysis. It was found that studies including children in advanced clinical stages (III&IV) had a 2.27 times higher risk of developing active

TB, as indicated by a meta-analysis of four studies. This finding is consistent with several meta-analyses reported in Ethiopia[60-63]. This might be because the advanced clinical stage of III&IV will have a low CD4 count and finally be unable to define the lethal opportunistic infection incidence during successive follow-ups up including TB [64].

The risk of developing active TB was 4.26 times higher for HIV-infected children with Hgb ≤10mg/dl compared to those with Hgb >10mg/dl, as indicated by the six meta-analysis studies included. This is consistent with the previous study findings [9, 18, 65]. This might be due to that anemia leads to the development of infections including TB by impairing the function of hemoglobin levels and exposure indirectly to the incidence of opportunistic infection.

The final report of this meta-analysis indicated children living with HIV/AIDS who missed or did not receive CPT had a four-fold higher risk of developing active tuberculosis (TB) as compared with counter groups. This is consistent with previously reported mat-analysis in Ethiopia [9, 16, 66]. This might be due to cotrimoxazole, which is prescribed as preventive therapy for HIV-infected children to counteract weakened immunity and reduce lethal opportunistic infections including Pneumocystis jirovecii pneumonia (PCP), toxoplasmosis, and TB and playing a crucial role in improving health outcomes and lowering morbidity and mortality associated with HIV/AIDS. In summary, prescribing cotrimoxazole aims to enhance immune function, diminish the risk of opportunistic infections, and improve overall health outcomes in the context of compromised immunity due to HIV.

The final report of this meta-analysis indicated that children living with HIV/AIDS who missed or did not receive cotrimoxazole preventive therapy (CPT) had a four-fold higher risk of developing active tuberculosis (TB) compared to the control groups. This finding is consistent with previously reported meta-analyses in Ethiopia [9, 16, 66] and CPT is recommended for HIV-positive children to prevent the progression of latent TB infection to active TB disease. It significantly reduces the risk of developing active TB in this population. Implementing IPT can help reduce the burden of TB and improve health outcomes for HIV-positive children.

Consistent with previous studies[25, 45, 46, 51, 67-71], concurrent administering of IPT after ruled-out of active TB symptoms with HAART demoted more than 90% of TB-associated incidence of morbidity and mortality [3, 34, 35]. This might be because IPT with HAART administration is effective in reducing the reactivation of latent mycobacteria in the lungs, thereby reducing the incidence of tuberculosis-associated morbidity and mortality in children. However, some challenges need to be addressed however completion rate is low~~er~~ by patients [25, 72].

In contrast to previous systematic review findings[9, 66] and primary studies reported [25, 45, 46, 51, 67-71]this meta-regression found no significant association between declined CD4 count (≤200 cells/~~ml~~), age of patients, duration of follow-up, comorbidity status, and functional status with the risk developing active TB in HIV co-infection children. This might be related to the methodological differences, heterogeneity of included study populations, sample size limitations, publication bias, and unaccounted factors, and further experimental studies are highly needed to better understand this relationship

Strengths and Limitations of the Study

The strengths of this study include an extensive search strategy, clear inclusion criteria, and the involvement of three independent authors in the quality assessment. However, there are several methodological limitations to consider. Firstly, there is a scarcity of well-described TB reports among studies in HIV-positive children compared to similar studies in adults. Furthermore, limitations such as reliance on clinical stratification or non-laboratory-supported staging, sub-standard diagnostic capacities in health facilities, a small number of included studies, and the use of retrospective data may potentially impact the validity of the results.

Conclusion and recommendation

More than one in ten HIV-infected children in Ethiopia develop active TB. Risk factors include advanced WHO clinical stages IV and III, as well as low hemoglobin levels. Effective management involves concurrent and continuous use of CPT, IPT, and ART for children living with HIV. Adherence to screening and management guidelines, including isoniazid and co-trimoxazole prevention, is crucial for healthcare professionals. Policymakers should enhance TB prevention strategies for at-risk children, and parents should prioritize feeding, adherence, and nutritional support for better outcome

Abbreviation

TB: Tuberculosis, WHO; Whorl Health Organization, FOM; Federal Ministry of Health, HIV; human immune deficiency varies, HAAR: highly active antiretroviral therapy, WHO; Whorl Health Organization, IPT, isoniazid preventive Therapy CPT, co-trimoxazole preventive therapy OIS; opportunistic infection

Acknowledgment

We would like to thank Woldia University College of Health Science Research and Community Service for their support of met analysis software training

Authors contribution

FK designed the study, conducted part of the search strategy and study selection, conducted the meta-analysis and data synthesis, and wrote the manuscript. FK and BK have contributed to the search strategy, article selection, report writing, and final revised full manuscript. Finally, all authors read, commence, and approve the final revised submissions.

Funding

The authors received no specific funding for this work.

Declaration

Ethics approval and consent to participate

Not applicable to this report

Consent for publication

Not applicable to this report

Computing interest

The authors have declared that no competing interests exist.

Data availability

All relevant data are within the paper and its Supporting Information files.

Supporting files

S1= PRISMA 2020 (DOCX)

S2= All relevant data are within the paper and its Supporting Information files **(DOCX)**

Reference

[1] WHO, Global Tuberculosis reporT 2022, https://iris.who.int/bitstream/handle/10665/363752/9789240061729-eng.pdf?sequence=1 (2022).

[2] WHO, Global tuberculosis report 2023, https://www.who.int/teams/global-tuberculosis-programme/tb-reports (2023).

[3] F. Kebede, B. Kebede, T. Kebede, M. Agmasu, Effect of Isoniazid Preventive Therapy on the Incidence of Tuberculosis among Seropositive Children Attending HIV/AIDS Care in Two General Hospitals, Northwest Ethiopia, 2021, Journal of tropical medicine 2021 (2021) 9996953.

[4] F. Kebede, H. Tarekegn, M. Molla, D. Jara, A. Abate, Incidence and Predictors of Pulmonary Tuberculosis among Children Who Received Antiretroviral Therapy (ART), Northwest Ethiopia: A Multicenter Historical Cohorts Study 2009-2019, Journal of tropical medicine 2022 (2022) 9925693.

[5] F. Kebede, B. Kebede, T. Kebede, M. Agmasu, Effect of Isoniazid Preventive Therapy on the Incidence of Tuberculosis among Seropositive Children Attending HIV/AIDS Care in Two General Hospitals, Northwest Ethiopia, 2021, Journal of tropical medicine 2021 (2021) 9996953.

[6] C. Schutz, D. Barr, B.B. Andrade, M. Shey, A. Ward, S. Janssen, R. Burton, K.A. Wilkinson, B. Sossen, K.F. Fukutani, M. Nicol, G. Maartens, R.J. Wilkinson, G. Meintjes, Clinical, microbiologic, and immunologic determinants of mortality in hospitalized patients with HIV-associated tuberculosis: A prospective cohort study, PLoS medicine 16(7) (2019) e1002840.

[7] G. Marley, X. Zou, J. Nie, W. Cheng, Y. Xie, H. Liao, Y. Wang, Y. Tao, J.D. Tucker, S. Sylvia, R. Chou, D. Wu, J. Ong, W. Tang, Improving cascade outcomes for active TB: A global systematic review and meta-analysis of TB interventions, PLoS medicine 20(1) (2023) e1004091.

[8] M.K. Jemberu Nigussie1Jemberu Nigussie1, Goitom Halefom3 Haven Hadush2 Mekuria Kassa, Goitom Halefom, Haven Hadush,, B.G.y.a.B. Grima1, Predictors of Mortality Among Children Co-Infected with Tuberculosis and Human Immunodeficiency Virus in Region, North Ethiopia, Retrospective Follow-Up Study, med J Sci & Tech Res | BJSTR. MS.ID.006252. ISSN: 2574 -1241(DOI: 10.26717/BJSTR.2021.39.006252) (2021).

[9] G.M. Belay, C.A. Wubneh, Childhood tuberculosis treatment outcome and its association with HIV co-infection in Ethiopia: a systematic review and meta-analysis, Tropical Medicine and Health 48(1) (2020) 7.

[10] E.S. Chanie, G.A. Gelaye, T.Y. Tadesse, D.G. feleke, W.T. Admas, E. Molla Alemu, M.M. Azanaw, S.A. Tiruneh, A.D. Gebremariam, B.M. Birhane, W.A. Bayih, G. Aragie, Estimation of lifetime survival and predictors of mortality among TB with HIV co-infected children after test and treat strategies launched in Northwest, Ethiopia, 2021; a multicentre historical follow-up study, PLOS ONE 16(12) (2021) e0258964.

[11] N. Ford, A. Matteelli, Z. Shubber, S. Hermans, G. Meintjes, B. Grinsztejn, G. Waldrop, K. Kranzer, M. Doherty, H. Getahun, TB as a cause of hospitalization and in-hospital mortality among people living with

HIV worldwide: a systematic review and meta-analysis, Journal of the International AIDS Society 19(1) (2016) 20714.

[12] T. Sime, L. Oljira, A. Diriba, G. Firdisa, W. Gezimu, Effect of active tuberculosis on the survival of HIV-infected adult patients who initiated antiretroviral therapy at public hospitals of Eastern Ethiopia: A retrospective cohort study, PLoS One 17(10) (2022) e0277021.

[13] T.G. Wondmeneh, A.T. Mekonnen, The incidence rate of tuberculosis and its associated factors among HIV-positive persons in Sub-Saharan Africa: a systematic review and meta-analysis, BMC infectious diseases 23(1) (2023) 613.

[14] A.M.H.T. Mohammedaman Mama, Hawa Mohammed and Endeshaw Erbo, Prevalence of Pulmonary Tuberculosis and Associated Factors Among HIV Positive Patients Attending Antiretroviral Therapy Clinic at Arba Minch General Hospital, Southern Ethiopia, The Open Microbiology Journal, 12, 163-171 12, 163-171(DOI: 10.2174/1874285801812010163) (2018).

[15] T. Kiros, E. Dejen, M. Tiruneh, T. Tiruneh, T. Eyayu, S. Damtie, K. Amogne, Magnitude and Associated Factors of Pulmonary Tuberculosis Among HIV/AIDS Patients Attending Antiretroviral Therapy Clinic at Debre Tabor Specialized Hospital, Northwest Ethiopia, 2019, HIV AIDS (Auckl) 12 (2020) 849-858.

[16] A. Kassa, A. Teka, A. Shewaamare, D. Jerene, Incidence of tuberculosis and early mortality in a large cohort of HIV infected patients receiving antiretroviral therapy in a tertiary hospital in Addis Ababa, Ethiopia, Transactions of the Royal Society of Tropical Medicine and Hygiene 106(6) (2012) 363-370.

[17] M.M. Azanaw, N.M. Derseh, G.S. Yetemegn, D.A. Angaw, Incidence and predictors of tuberculosis among HIV patients after initiation of antiretroviral treatment in Ethiopia: a systematic review and meta-analysis, Trop Med Health 49(1) (2021) 18.

[18] B. Tesfaye, A. Alebel, A. Gebrie, A. Zegeye, C. Tesema, B. Kassie, The twin epidemics: Prevalence of TB/HIV co-infection and its associated factors in Ethiopia; A systematic review and meta-analysis, PLoS One 13(10) (2018) e0203986.

[19] D. Gebreyes, A survival analysis of prognostic determinant factors of time-to-death of HIV/TB co-infected patients under HAART followed-up in a public hospital in Ethiopia, HIV & AIDS Review. International Journal of HIV-Related Problems 22(2) (2023) 110-130.

[20] M.E. Lelisho, T.W. Wotale, S.A. Tareke, B.D. Alemu, S.S. Hassen, D.M. Yemane, B.B. Korsa, N.G. Bedaso, Survival rate and predictors of mortality among TB/HIV co-infected adult patients: retrospective cohort study, Scientific reports 12(1) (2022) 18360.

[21] E. Seyoum, M. Demissie, A. Worku, A. Mulu, Y. Berhane, A. Abdissa, Increased Mortality in HIV Infected Individuals with Tuberculosis: A Retrospective Cohort Study, Addis Ababa, Ethiopia, HIV/AIDS (Auckland, N.Z.) 14 (2022) 143-154.

[22] M. Abdu, Y. Ali, S. Anteneh, M. Yesuf, A. Birhanu, S. Mohamed, A. Hussien, Determinant factors for the occurrence of tuberculosis after initiation of antiretroviral treatment among adult patients living with HIV at Dessie Referral Hospital, South Wollo, Northeast Ethiopia, 2020. A case-control study, PLoS One 16(3) (2021) e0248490.

[23] J.U.N.P.o.H.A. (UNAIDS, UNAIDS Global AIDS Update 2022, UNAIDS GLOBAL AIDS UPDATE (2022).

[24] Y.Z. Weiwei Mua, Xin Suna, Ye Maa, Lan Yua, Xia Liua, Decai Zhaoa, Zhihui Doua, Hua, F.F. Zhangab, Incidence and associated factors of pulmonary tuberculosis in HIV-infected children after highly active antiretroviral therapy (HAART) in China: a retrospective study, AIDS Care: Psychological and Socio-medical Aspects of

AIDS/HIV Psychological and Socio-medical Aspects of AIDS/HIV, 26:9,

1127-1135, DOI: 10.1080/09540121.2014.882492 (2015,).

[25] F. Kebede, T. Kebede, B. Kebede, A. Abate, D. Jara, B. Negese, T. Shaweno, Time to Develop and Predictors for Incidence of Tuberculosis among Children Receiving Antiretroviral Therapy, Tuberc Res Treat 2021 (2021) 6686019.

[26] Mehretu Belayneha, Kalayta Gidayb, Hailemariam Lemmac, Treatment outcome of human immunodeficiency virus and tuberculosis co-infected patients in public hospitals of eastern and southern zone of

Tigray region, Ethiopia, b r a z j i n f e c t d i s . 2 0 1 5;1 9(1):47–51 (http://dx.doi.org/10.1016/j.bjid.2014.09.00) (2014).

[27] D.S.a.A. Worku2, Tuberculosis treatment survival of HIV positive TB patients on directly observed treatment short-course in Southern Ethiopia: A retrospective

cohort study, BMC Research Notes 2012, 5:682 http://www.biomedcentral.com/1756-0500/5/682 (2012).

[28] FMOH, NATIONALCONSOLIDATED GUIDELINES FOR COMPREHENSIVE HIV PREVENTION, CARE AND TREATMENT, Manuel Accesed septemebr 2021 (2018).

[29] E.S. Chanie, W.A. Bayih, B.M. Birhan, D.M. Belay, G. Asmare, T. Tiruneh, Y.A.A. Aynalem, B.B. Abat, S. Asnakew, M. Mekie, G.Y. Yitbarek, F.A. GebreEyesus, Incidence of advanced opportunistic infection and its predictors among HIV infected children at Debre Tabor referral Hospital and University of Gondar Compressive specialized hospitals, Northwest Ethiopia, 2020: A multicenter retrospective follow-up study, Heliyon 7(4) (2021) e06745.

[30] F.T.Y. Deyas, Effect of highly active antiretroviral treatment on TB incidence among HIV infected children and their clinical profile, retrospective cohort study, Sout West Ethiopia, Scientific Reports 10:21468 https://doi.org/10.1038/s41598-020-78466-0 (2020).

[31] D. Tsegaye, S. Wude, T. Kebede, S. Adane, T. Shumet, F. Kebede, Epidemiological survival pattern, risk factors, and estimated time to develop tuberculosis after test and treat strategies declared for children living with human immune deficiency virus, Indian Journal of Tuberculosis (2023).

[32] H.T.A. Kebede Embaye Gezae1 Kidu Gebremeskel2, Letekirstos Gebre-Egziabher Gebretsadik1and Abrha, Predictors of Accelerated Mortality of Tb/Hiv Co- Infected Patients on Art in Mekelle, Ethiopia: an 8 Years Retrospective Follow- Up Study, Annals of Biostatistics & Biometric Applications SN: 2641-6336 (DOI: 10.33552/ABBA.2019.03.000572) (2019).

[33] Z. Dawit, S. Abebe, S. Dessu, M. Mesele, S. Sahile, D. Ajema, Incidence and predictors of mortality among children co-infected with tuberculosis and human immunodeficiency virus at public hospitals in Southern Ethiopia, PLoS One 16(6) (2021) e0253449.

[34] Temesgen Getaneh a, Ayenew Negesse b, Getenet Dessie c, Melaku Desta d, The impact of tuberculosis co-infection on virological failure among adults living with HIV in Ethiopia: A systematic review and meta-analysis, Journal of Clinical Tuberculosis and Other Mycobacterial Diseases 27 (2022) 100310 (2022).

[35] D.G. Assefa, E.D. Zeleke, D. Bekele, D.A. Ejigu, W. Molla, T.T. Woldesenbet, A. Aynalem, M. Abebe, A. Mebratu, T. Manyazewal, Isoniazid Preventive Therapy for Prevention of Tuberculosis among People Living with HIV in Ethiopia: A Systematic Review of Implementation and Impacts, International journal of environmental research and public health 20(1) (2022).

[36] P. checklist, PRISMA_2020_expanded_checklist for systemic reviwe and meta-analysis studies reporting BMJ 2021;372:n16 BMJ 2021;372:n71. doi: 10.1136/bmj.n71 (2021).

[37] Professor GA Wells, NEWCASTLE-OTTAWA QUALITY ASSESSMENT SCALE COHORT STUDIESf>, J Educ Eval Health Prof 2016; 13: 40 • https://doi.org/10.3352/jeehp.2016.13.40 (2016).

[38] J.B. Institute, JBI_Critical_Appraisal-Checklist_for_Analytical_Cross_Sectional_Studies, Checklist for Analytical Cross Sectional Studies, http://joannabriggs.org/research/critical-appraisal-tools.html www.joannabriggs.org (2017).

[39] M. Egger, G. Davey Smith, M. Schneider, C. Minder, Bias in meta-analysis detected by a simple, graphical test, BMJ 315(7109) (1997) 629-634.

[40] J.J.B.S.A. Doi, MetaXL User Guide Version 5.3, Sunrise Beach, Queensland, Australia, 2011-2016 Version. 2016;4:2011–6.(file:///C:/Users/exite/OneDrive/Desktop/MetaXL%20User%20Guide.pdf) (2016).

[41] S.G. Thompson, J.P. Higgins, How should meta-regression analyses be undertaken and interpreted?, Statistics in medicine 21(11) (2002) 1559-73.

[42] M. Borenstein, L.V. Hedges, J.P. Higgins, H.R. Rothstein, A basic introduction to fixed-effect and random-effects models for meta-analysis, Research synthesis methods 1(2) (2010) 97-111.

[43] S. Duval, R. Tweedie, A Nonparametric "Trim and Fill" Method of Accounting for Publication Bias in Meta-Analysis, Journal of the American Statistical Association 95(449) (2000) 89-98.

[44] E.H.E.a.N.T. Aklilu Endalamaw*, Incidence of tuberculosis in children on antiretroviral therapy: a retrospective cohort study, BMC Res Notes 11:745 https://doi.org/10.1186/s13104-018-3846-z (2018).

[45] Y.M. Alemu, G. Andargie, E. Gebeye, High Incidence of Tuberculosis in the Absence of Isoniazid and Cotrimoxazole Preventive Therapy in Children Living with HIV in Northern Ethiopia: A Retrospective Follow-Up Study, PLoS One 11(4) (2016) e0152941.

[46] S.G. Ayalaw, K.A. Alene, A.A. Adane, Incidence and Predictors of Tuberculosis among HIV Positive Children at University of Gondar Referral Hospital, Northwest Ethiopia: A Retrospective Follow-Up Study, Int Sch Res Notices 2015 (2015) 307810.

[47] D.J. Emil Westerlund, Zewdie Mulissa Inger Hallström and Bernt Lindtjørn5, Pre-ART retention in care and prevalence of tuberculosis among HIV-infected children at a district hospital in southern Ethiopia, BMC pediatrics 14:250 http://www.biomedcentral.com/1471-2431/14/250 (2014).

[48] E.S.C. Endalk B, Fisha A.G, Gebeyaw Biset et.al Incidence and predictors of tuberculosis among children on antiretroviral therapy at northeast Ethiopia comprehensive specialized hospitals, 2022; A multicenter retrospective follow-up study>, Heliyon 8 (2022) e12001 https://doi.org/10.1016/j.heliyon.2022.e12001 (2022).

[49] Y.D.a.D.A. Firew Tiruneh*, tuberculosis-incidence-among-hiv-infected-children-on-haart-and-their-clinical-profile-retrospective-cohort-study-south-, Journal of AIDS & Clinical Research Research Article Volume 11:3,2020 DOI: 10.37421/jar.2020.11.808 (2020).

[50] T.M.D. Masino Tessu Beshir1 Aklil Hailu Beyene2 Kenean Getaneh Tlaye3, Incidence and predictors of tuberculosis among HIV- positive children at Adama Referral Hospital and Medical College, Oromia, Ethiopia: a retrospective follow-up study, epih.org 41, Article ID: e2019028, 8 (https://doi.org/10.4178/epih.e2019028) (2017).

[51] M.W. Melkamu, M.T. Gebeyehu, A.D. Afenigus, Y.T. Hibstie, B. Temesgen, P. Petrucka, A. Alebel, Incidence of common opportunistic infections among HIV-infected children on ART at Debre Markos referral hospital, Northwest Ethiopia: a retrospective cohort study, BMC infectious diseases 20(1) (2020) 50.

[52] D.A. Mequanente, P. Srinivasan, G. Mallika, P.M.T. Ansari, M. Wale, Incidence of Opportunistic Infections among HIV-infected Children on ART at Gondar University Specialized Hospital, Ethiopia, Indian Journal Of Science And Technology 15(34) (2022) 1675-1682.

[53] B. Solomon M. Abay, MSc, PhD1, Kebede Deribe, BSc, MPH2,3,, B. Ayalu A. Reda, MSc4, Sibhatu Biadgilign, BSc, MPH5, The Effect of Early Initiation of Antiretroviral Therapy in TB/HIV-Coinfected Patients: A Systematic Review and Meta-Analysis, Journal of the International Association of Providers of AIDS Care 2015, Vol. 14(6) 560–570 DOI: 10.1177/2325957415599210 (2015).

[54] G. Diriba, A. Alemu, K. Eshetu, B. Yenew, D.F. Gamtesa, H.H. Tola, Bacteriologically confirmed extrapulmonary tuberculosis and the associated risk factors among extrapulmonary tuberculosis suspected patients in Ethiopia: A systematic review and meta-analysis, PloS one 17(11) (2022) e0276701.

[55] I.F. Ana Maria Tavares*, Isabel Couto, Diana Machado, Miguel Viveiros, AnaB. Abecasis, So, n. Dias, HIV and tuberculosis co-infection among migrants in Europe: A systematic review on the prevalence, incidence and mortalit, PLoS ONE 12(9):e0185526. https://doi.org/10.1371/journal.pone.0185526 (2017).

[56] L. Pimpin, L.N. Drumright, M.E. Kruijshaar, I. Abubakar, B. Rice, V. Delpech, V. Hollo, A. Amato-Gauci, D. Manissero, C. Ködmön, Tuberculosis and HIV co-infection in European Union and European Economic Area countries, European Respiratory Journal 38(6) (2011) 1382.

[57] M.-R. B-Lajoie, O. Drouin, G. Bartlett, Q. Nguyen, A. Low, G. Gavriilidis, P. Easterbrook, L. Muhe, Incidence and Prevalence of Opportunistic and Other Infections and the Impact of Antiretroviral Therapy Among HIV-infected Children in Low- and Middle-income Countries: A Systematic Review and Meta-analysis, Clinical Infectious Diseases 62(12) (2016) 1586-1594.

[58] I. Law, K. Floyd, T.B.P.S.G. the African, National tuberculosis prevalence surveys in Africa, 2008–2016: an overview of results and lessons learned, Tropical Medicine & International Health 25(11) (2020) 1308-1327.

[59] Augustine O. Ebonyi1, A.S. Sagay7, E.U.E. , P.O.U. , P.I.O. , Prevalence of and risk factors for pulmonary tuberculosis among newly diagnosed HIV-1 infected Nigerian children, GERMS 6(1) • March 2016 • page 21 (2016).

[60] Animut Alebel1, Daniel Demant2,3, Pammla Petrucka4,5 and David Sibbritt2, Effects of undernutrition on mortality and morbidity among adults living with HIV in sub-Saharan Africa: a systematic review and meta-analysis, BMC Infectious Diseases (2021) 21:1 https://doi.org/10.1186/s12879-020-05706-z (2021).

[61] N.M.D. Melkalem Mamuye Azanaw1, Gebeyehu Shumuye Yetemegn2 and, D.A. Angaw3*, Incidence and predictors of tuberculosis among HIV patients after initiation of antiretroviral treatment in Ethiopia: a systematic review and meta-analysis, Tropical Medicine and Health 49:18(https://doi.org/10.1186/s41182-021-00306-2) (2021).

[62] M. Straetemans, P. Glaziou, A.L. Bierrenbach, C. Sismanidis, M.J. van der Werf, Assessing tuberculosis case fatality ratio: a meta-analysis, PLoS One 6(6) (2011) e20755.

[63] A.G.-W. Marriott Nliwasa Peter MacPherson4, 3, Mphatso Mwapasa1, Katherine Horton3,, Jon Ø Odland5, Clare Flach7 and Elizabeth L. Corbett1,, High HIV and active tuberculosis prevalence and increased mortality risk in adults with symptoms of TB: a systematic review and meta-analyses, Journal of the International AIDS Society 2018, 21:e25162 https://doi.org/10.1002/jia2.25162 (2018).

[64] S. Wude, D. Tsegaye, S. Adane, T. Kebede, F. Kebede, T. Shumet, Epidemiological Survival Pattern, Risk Factors, And Estimated Time To Develop Tuberculosis After Test And Treat Strategies Declared For Children Living With Human Immune Deficiency Virus, Indian Journal of Tuberculosis (2023).

[65] N.M.D. Melkalem Mamuye Azanaw1, Gebeyehu Shumuye Yetemegn2 and Dessie Abebaw Angaw, Incidence and predictors of tuberculosis among HIV patients after initiation of antiretroviral treatment in Ethiopia: a systematic review and meta-analysis, Tropical medicine and health 49:18(https://doi.org/10.1186/s41182-021-00306-2) (2021).

[66] M.M. Azanaw, N.M. Derseh, G.S. Yetemegn, D.A. Angaw, Incidence and predictors of tuberculosis among HIV patients after initiation of antiretroviral treatment in Ethiopia: a systematic review and meta-analysis, Tropical Medicine and Health 49(1) (2021) 18.

[67] M.T. Beshir, A.H. Beyene, K.G. Tlaye, T.M. Demelew, Incidence and predictors of tuberculosis among HIV-positive children at Adama Referral Hospital and Medical College, Oromia, Ethiopia: a retrospective follow-up study, Epidemiol Health 41 (2019) e2019028.

[68] F.T.Y. Deyas, Effect of highly active antiretroviral treatment on TB incidence among HIV infected children and their clinical profile, retrospective cohort study, South West Ethiopia, Scientific Reports 10:21468 https://doi.org/10.1038/s41598-020-78466-0 (2020).

[69] A. Endalamaw, E.H. Engeda, N. Tezera, Incidence of tuberculosis in children on antiretroviral therapy: a retrospective cohort study, BMC Research Notes 11(1) (2018) 745.

[70] Endalk Birrie Wondifraw a, Ermias Sisay Chanie b, FishaAlebel Gebreeyesus c, Gebeyaw Biset a, Birhanu Desu Tefera d, Mulusew Zeleke e, Incidence and predictors of tuberculosis among children on antiretroviral therapy at northeast Ethiopia comprehensive specialized hospitals, 2022; A multicenter retrospective follow-up study, Heliyon 8 (2022) e12001 Heliyon 8 (2022) e12001 (2022).

[71] E. Westerlund, D. Jerene, Z. Mulissa, I. Hallström, B. Lindtjørn, Pre-ART retention in care and prevalence of tuberculosis among HIV-infected children at a district hospital in southern Ethiopia, BMC pediatrics 14 (2014) 250.

[72] A.A. Kumar, A. De Costa, A. Das, G.A. Srinivasa, G. D'Souza, R. Rodrigues, Mobile Health for Tuberculosis Management in South India: Is Video-Based Directly Observed Treatment an Acceptable Alternative?, JMIR mHealth and uHealth 7(4) (2019) e11687.

Legendary for description of given figures

Figure 1: A PRISMA flow diagram for describing the flow of the chart of selected articles

Figure 2: Forest plot of the pooled prevalence of active TB I HIV-infected children

Figure 3: Forest plot of subgroup analysis by study setting for Active TB in HIV-infected children

Figure 4: Forest plot for subgroup analysis by follow-up time of active TB in HIV-infected children

Figure 5. Forest plotted for impact of missed IPT among active TB in HIV-infected children.

Figure 6. Forest plotted for the impact of missed CPT among active TB in HIV-infected children.

Figure 7: Funnel plot for active TB incidence among HIV-infected children

Legendary for description of given Tables

Table 1: Characteristics of included articles/studies reporting the prevalence of TB in HIV-positive children in Ethiopia after ART initiation

Table 2: Meta-regression analysis to identify sources of heterogeneity.

Supporting Information

S1= PRISMA 2020 (Preferred Reporting Items for Systematic Reviews and Meta-Analysis)

S2= "All relevant data are within the paper and its Supporting Information files" (DOCX

Click here to access/download;Figure;Figure =1.tif

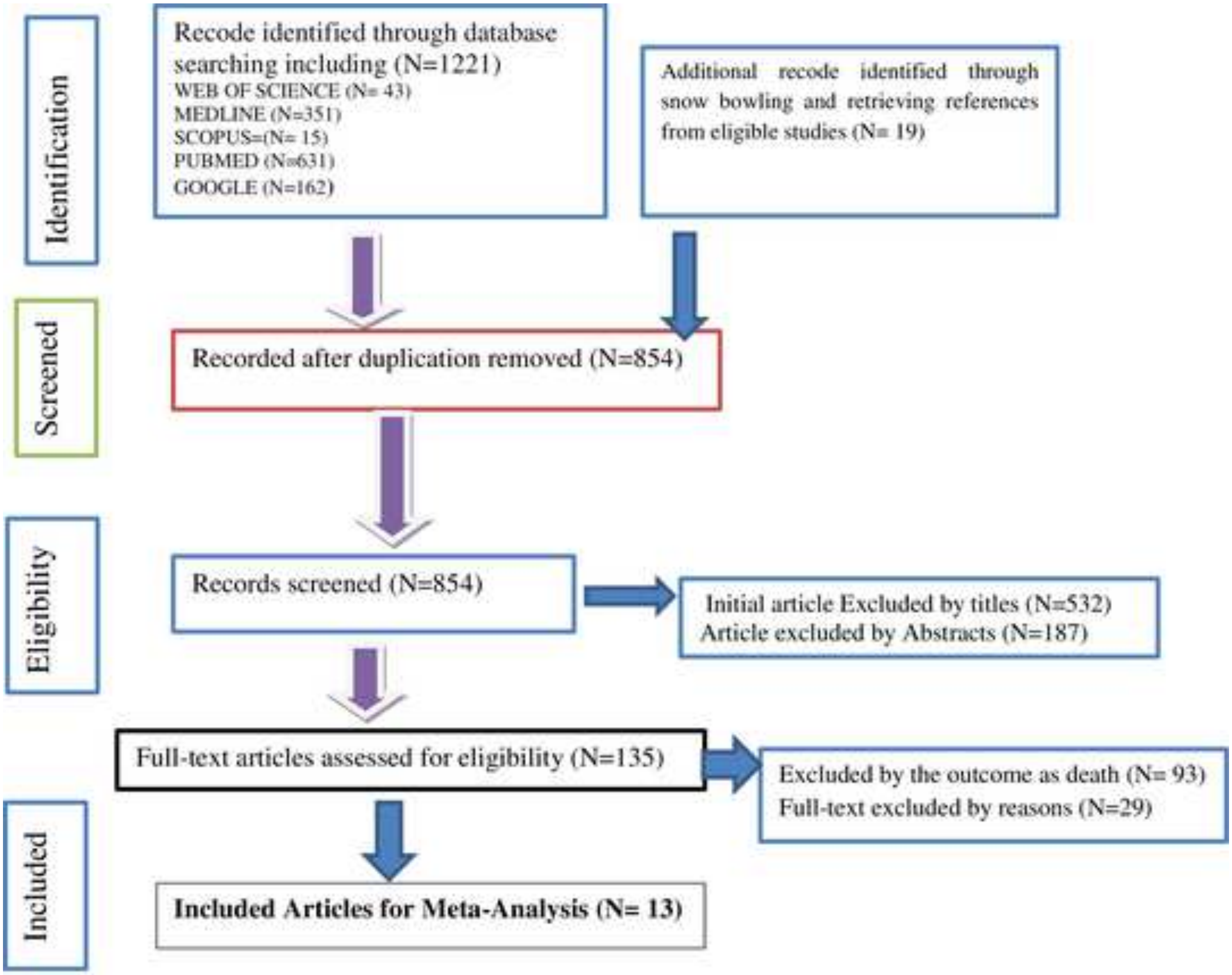

Figure 1: A PRISMA flow diagram for describing the flow chart of selected articles eligible for reviewing

F=3

Click here to access/download;Figure;Figure =3.tif

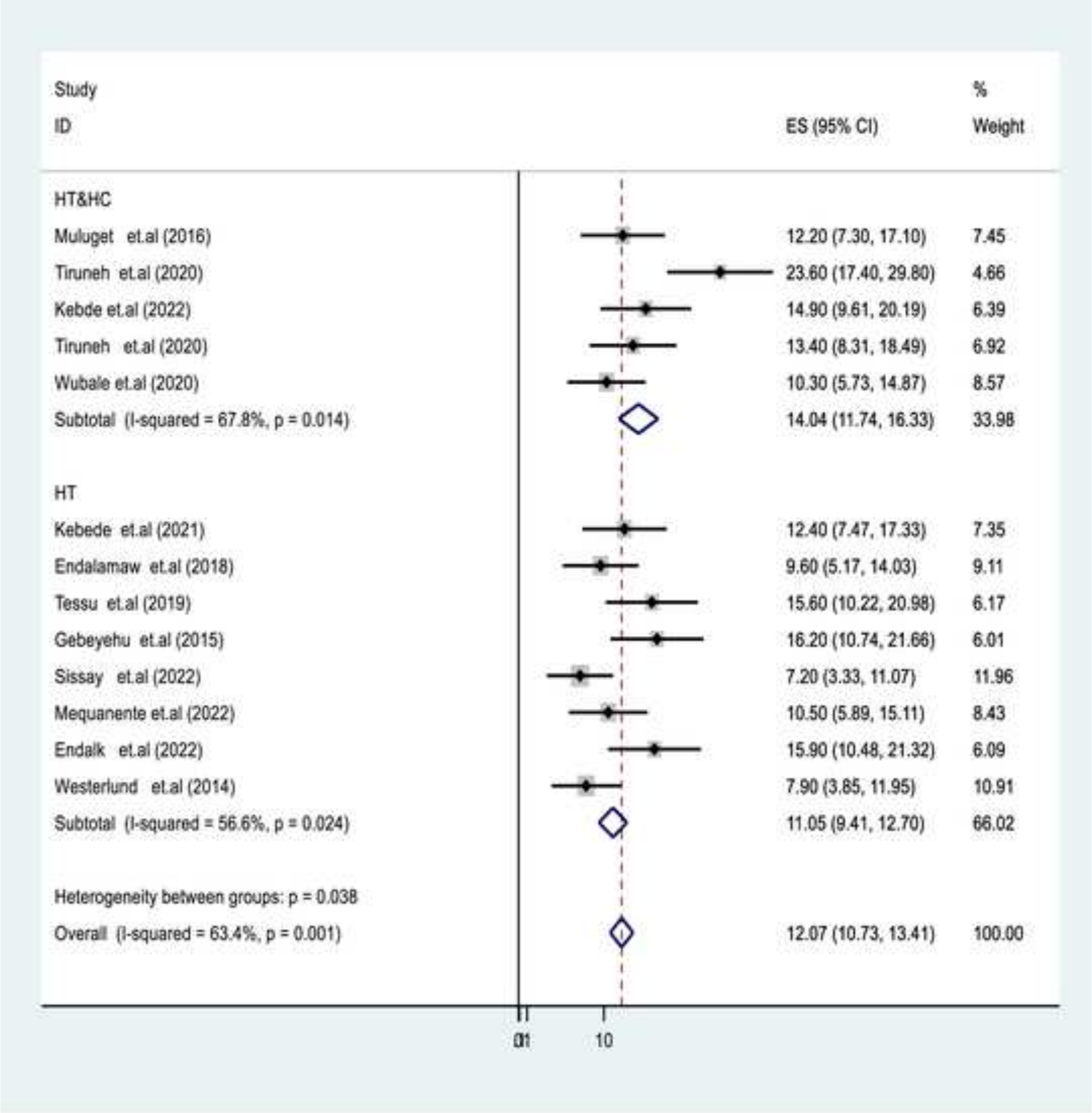

*HC= health center; HT=Hospital

Figure 3. Forest plot for subgroup by study setting for active TB among HIV-infected children

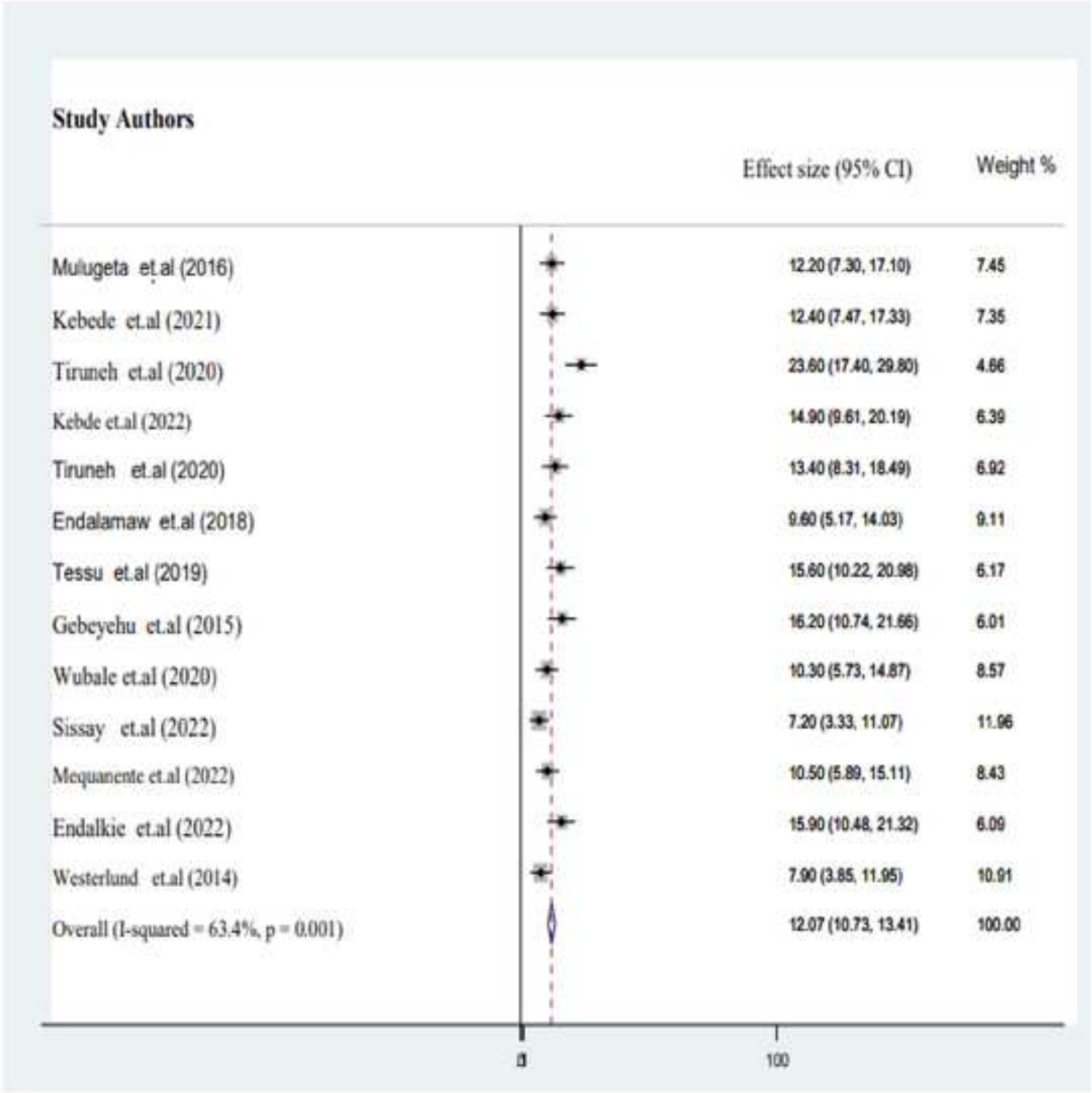

Figure 2. Forest plot of the pooled prevalence of active TB in HIV- infected children in Ethiopia

Click here to access/download;Figure;Figure =4.tif

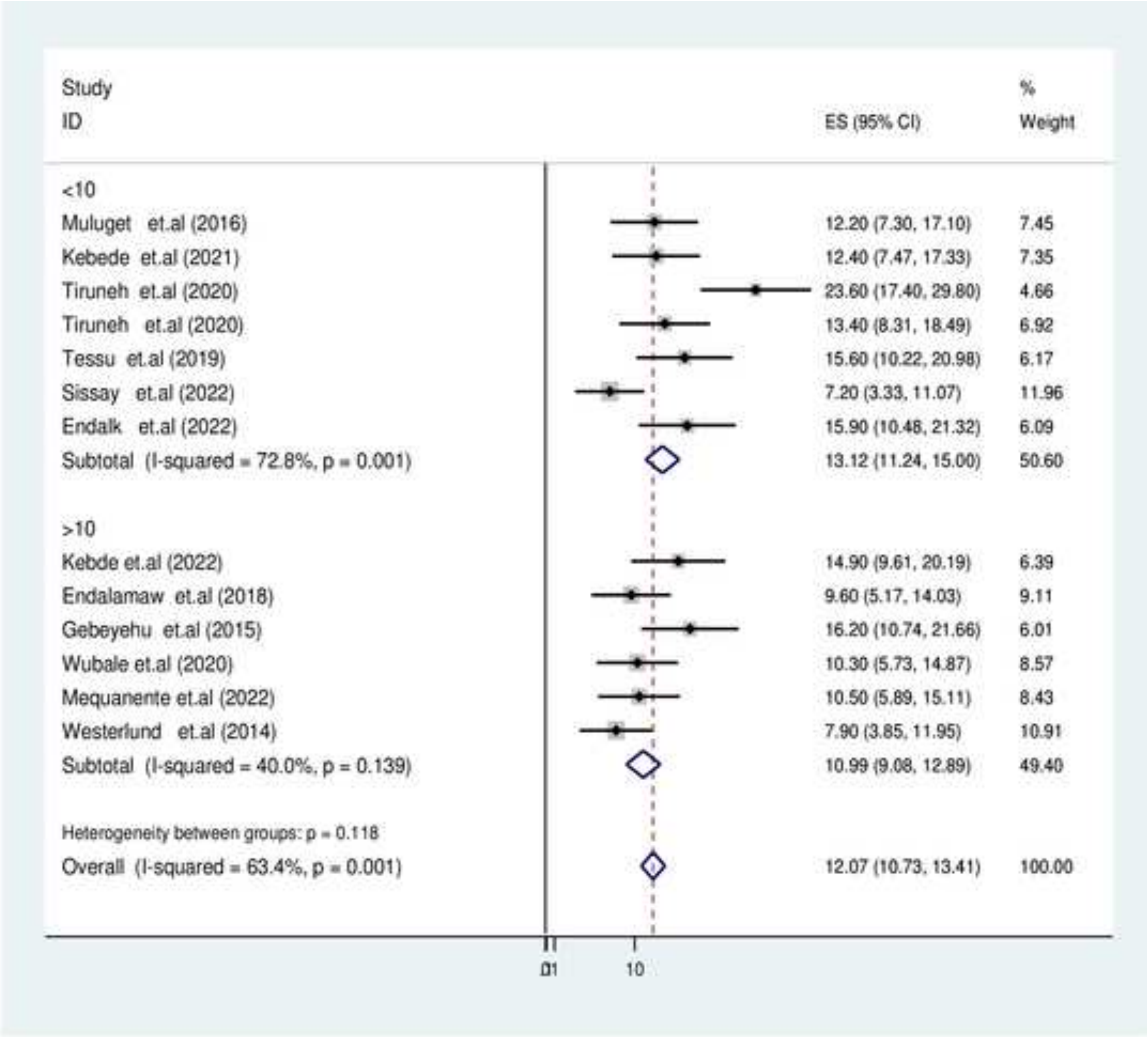

Figure 4: **Forest plot for subgroup by follow-up periods for active TB among HIV-infected children**

Click here to access/download;Figure;Figure=5.tif

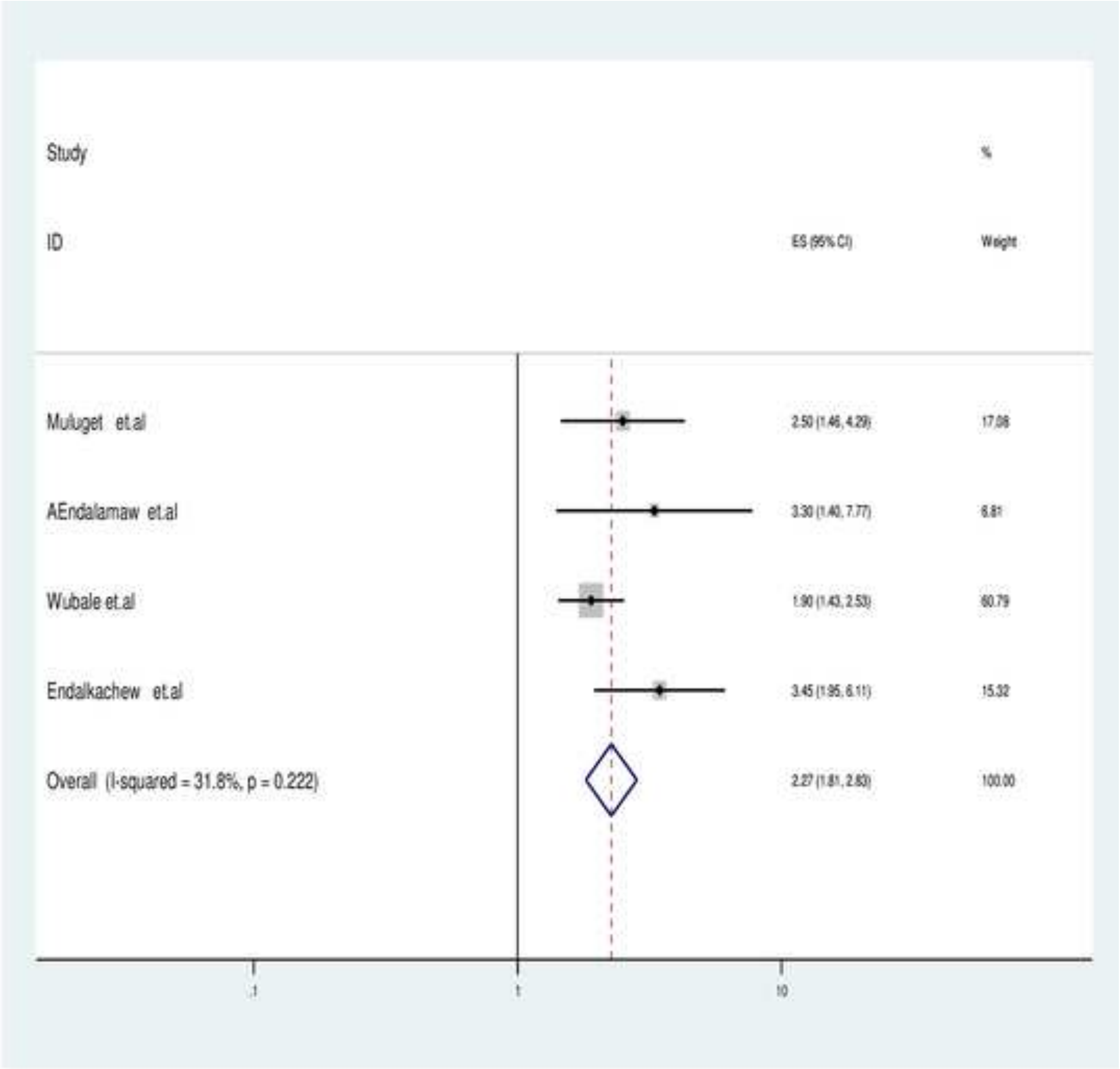

Fig 5. Forest plotted for impact of missed IPT among active TB in HIV-infected children.

F=6
Click here to access/download;Figure;Figure=6.tif

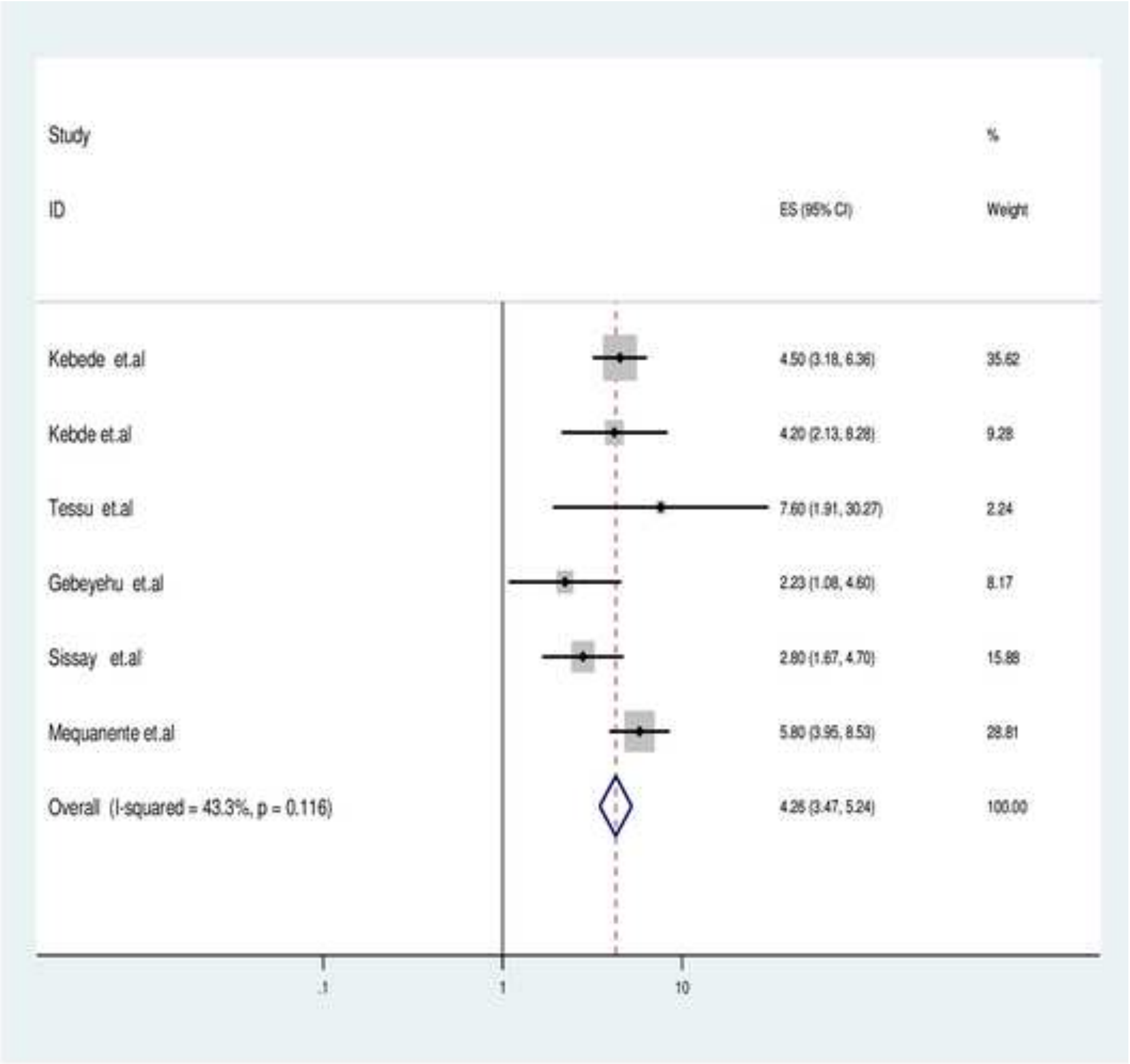

Fig6. Forest plotted for impact of missed CPT among active TB in HIV-infected children.

Click here to access/download;Figure;Figure=7.tif

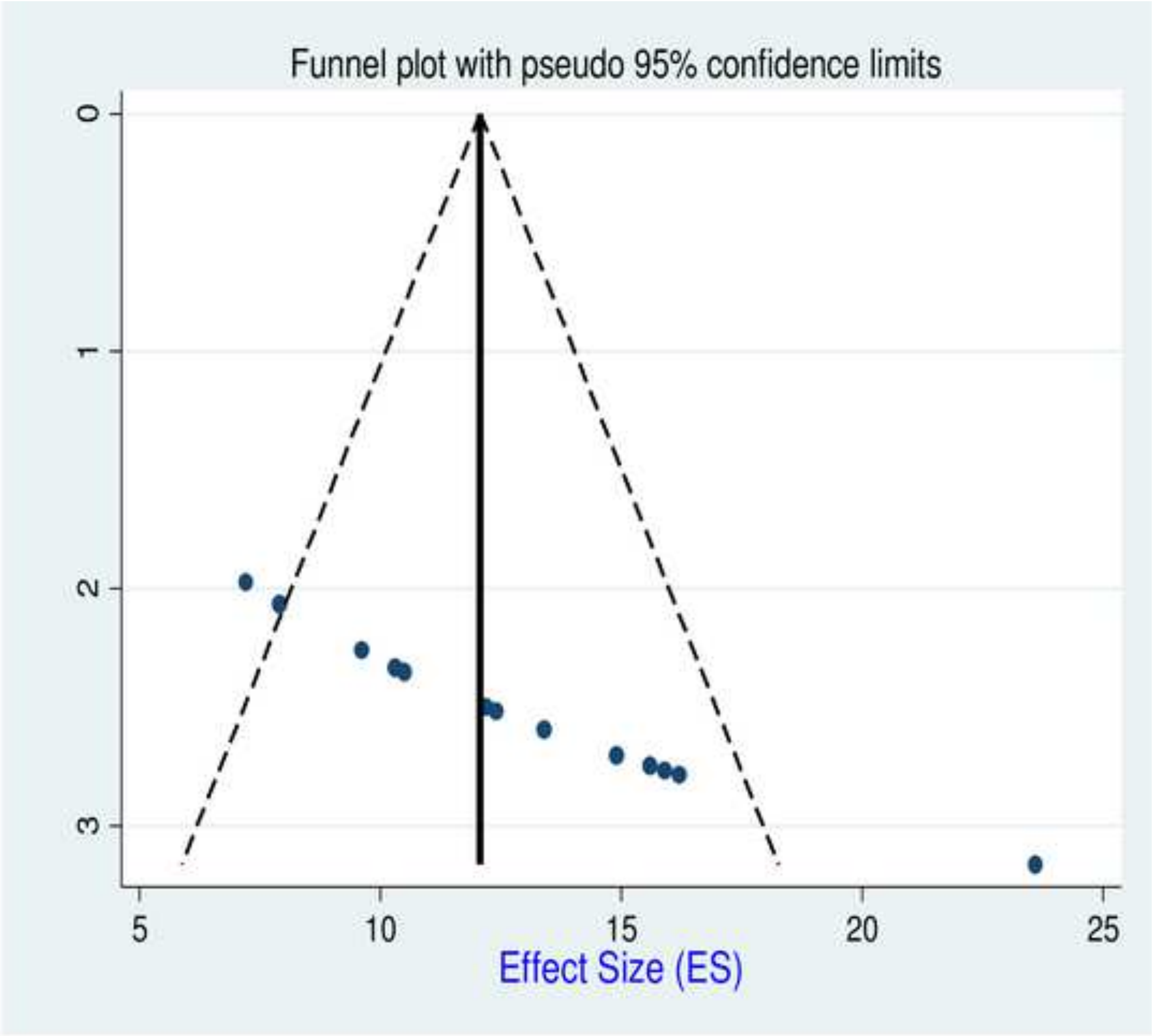

Fig 7: Funnel plot for active TB in HIV-infected children

s1= PRISMA check list

Click here to access/download

Supporting Information

S1= PRISMA cheklists 2020).doc

List of Tables

Click here to access/download
Supporting Information
List of Tabels.docx

S2=All relavant Data set are with in the paper

Click here to access/download
Supporting Information
S2=All relevant data are within the paper.xlsx

Meta-Analysis of Active Tuberculosis Occurrence among Children Living with HIV Post Anti-Retroviral Therapy Initiated in Ethiopia

~~ACTIVE TUBERCULOSIS PREVALENCE AFTER ANTI-RETROVIRAL THERAPY AMONG SEROPOSITIVE CHILDREN LIVING IN ETHIOPIA: A SYSTEMATIC REVIEW AND META-ANALYSIS~~

Fassikaw Kebede[1*], Dejen Tsegaye[2], Tsehay Kebede[3], Belete Negese[4] , Tesfaye Shumet[1]

~~Fassikaw Kebede[1*], Dejen Tsegaye[2], Tsehay Kebede[3], Belete Negese[4]~~

[1]College of Health Sciences, Woldia University, Woldia, Ethiopia

[2] College of Health Sciences, Debre Markos University, Ethiopia

[3] College of Social Science Bahir Dare University, Bahir Dare, Ethiopia

[4] College of Health Sciences, Debre Berhan University, Ethiopia

*Corresponding Author

Fassikaw Kebede

Email = fassikaw123@gmail.com

Abstract
Background:
Despite the effectiveness of antiretroviral treatment in reducing morbidty and mortality from opportunistic infections among children living living with HIV (CLHIV), tuberculosis (TB) remains a significant cause of mortality,accounting one in every three deaths. However, in Ethiopia, there is a lack of aggregated data on pooled prevalence of TB and HIV co-infection for children living with HIV. Therefore, this meta-analysis aims to estimate the prevalence of active TB in HIV-infected children nationwide after initiating ART between December 30, 2012, and January 1, 2023.

Despite antiretroviral treatment has shown a marked reduction in new tuberculosis (TB) incidence, due to their age category and immune-suppressing effects of HIV, children were more vulnerable to experiencing active TB. Although several small-scale studies have reported the co-infection rate of TB and HIV, there is no aggregated estimation after antiretroviral therapy among seropositive children. Thus, this systematic review and meta-analysis report aimed to estimate the pooled prevalence of active tuberculosis in HIV-infected children at national levels after ART started from December 30/2012 to January 1st 2023.

Methods;
A systematic review of observational studies was used in published articles from December 30/2012 to January 1st / 2023. PubMed, MEDLINE, WEB OF SCIENCE, Scopus, and Google Scholar were used to extract 1235 articles. PRISMA guideline was used for the final study. The extraction of data was summarized using descriptive analysis. We pooled TB prevalence and odds ratio (OR) estimates for risk factors after checking heterogeneity and publication biases. The heterogeneity across the studies was assessed using Cochran's Q test and I^2 statistic.

Results: -

A total of 13 studies were included for meta-analysis. The mean age of participants (N= 5834) was estimated at 8.2(±3.6) years. The overall pooled prevalence of tuberculosis was determined as 12.07 %(95%CI; 10.71 - 13.41; I^2 = 63.4%, P= 0.001). In the subgroup analysis, the estimated pooled TB prevalent showed a significant difference between hospital set-up and facility-based

studies, which was11.05% (95%CI: 9.4- 12.3) &14.1%; 95%CI: 11.74- 16.33), respectively. Significantly pooled estimated risk factors of TB occurrence by a meta-analysis were being advanced clinical stage III&IV (OR= 2.27: 95%CI: 1.81--- 2.83), and Hgb ≤10mg/dl (OR=4.26: 95CI: 3.4---5.24) were all predictors for active TB.

Conclusion;

Overestimation of active TB cases among HIV-positive children in Ethiopia was observed, with approximately one in ten affected. Risk factors for active TB included WHO clinical stages IV and III, as well as low hemoglobin levels. Effective management involves providing concurrent and continuous CPT (Cotrimoxazole preventive therapy), IPT (Isoniazid preventive therapy), and ART (antiretroviral therapy) for children living with HIV.

~~A significant number of HIV-positive children suffered from twine (TB and HIV) infection in Ethiopia. The risks of TB were significantly higher with studies including WHO clinical stage III &IV, and having Hgb ≤10mg/dl during metanalysis. Thus, HIV is a potential risk factor for pediatric TB and efforts should be strengthened for early diagnosing and treatment.~~

~~**Keywords:** HIV, Children, prevalence, Tuberculosis, Ethiopia~~

Introduction

Around the world, TB is the most common opportunistic infection and cause of morbidity and mortality for both adults and children living with HIV [1, 2]. The synergy between TB and HIV infection is strong; TB is the leading cause of death and HIV infection is the strongest risk factor for active TB occurrence through target reduction of CD4 count and cellular immunity [3, 4]. The waning of the immune system increases the endogenous reactivation of dormant TB bacilli in the lung[5]

Globally, in 2017, the twins epidemic of TB and HIV infection was responsible for 0.3 million deaths for children living with HIV [6-8] and had a risk of 15–22 times more than children without[9]. Despite breakthroughs in diagnosis and the widespread availability of chemotherapy, TB-related mortality in children accounts for 40% of global TB deaths, 25% of in-hospital deaths, and 18% of inpatient hospitalizations [8, 10-12].

In 2019, 1.7 million TB deaths were reported globally, making TB the largest cause of mortality from a single infectious agent (ranked higher than HIV/AIDS)[12, 13]. Africa is the second TB

burden region (25%) next to Southeast Asia (44%) [13, 14], and in which–a third of TB/HIV-associated deaths were reported from children living with HIV [12, 15, 16]. As well, in 2018, there were about 251,000 deaths from TB among PLWHIV, which accounts for 33% of the total deaths associated with HIV, which is much higher than the case fatality rate expected ≤ 5 % by WHO [17, 18].

In Sub-Saharan African countries, 10%–15% of the population suffered from the twine epidemic [19, 20]. The risk of developing TB disease is 21 times higher for people living with HIV and 51% lifetime risk of TB than those without HIV patients (1, 2). In Ethiopia in 2016, an estimated 710,000 people were living with HIV, and 62,000 of them were children living with HIV [21, 22]. Extra-pulmonary Tuberculosis (EPTB) is the most common kind of tuberculosis (TB) diagnosed and treated in seropositive children, and TB is responsible for one out of every four deaths in resource-limited settings [9, 23]. According to national profiles of TB and HIV patients, 11% of TB cases had HIV, while 9.1% of HIV patients had active TB [17, 24, 25]. Several small-scale studies in Ethiopia found TB incidence ranging from 7.2% in Amhara [26] to 23.6% in the south [27] regions. Despite concomitant administration of ART with IPT demoting> 80% of active TB[5], due to their age and immune-suppressing of HIV, children experienced active TB incidence [28]. There have been numerous small-scale studies on the rate of TB/HIV co-infection [1, 29-32], but there is no pooled TB estimate reported in Ethiopia. Thus, this systematic review and meta-analysis report aimed to estimate the pooled prevalence of active tuberculosis in HIV-infected children at national levels after ART started from December 30/2012 to January 1st, 2023

~~**Review question**~~

~~1. What are the pooled predictors of active tuberculosis incidence after ART was commenced in Ethiopian children living with HIV/AIDS from December 30, 2012, to January 1, 2023?~~

~~2. What is the estimated pooled prevalence of active tuberculosis after ART among HIV-infected children in Ethiopia from December 30, 2012, to January 1, 2023?~~

Objectives of the study

Objectives

- Determining pooled Tuberculosis-incidence in HIV-positive children in Ethiopia
- Identifying Predictors for TB Incidence among Children Living with HIV in Ethiopia

~~The main objective of our systematic review and meta-analysis is to estimate the pooled prevalence of active TB among HIV infected children at national levels after ART started from December 30/2012 to January 1st, 2023~~

We conducted a systematic review of published and unpublished articles from multiple databases, including PubMed, HINARY, WEB OF SCIENCE, Africa Journals Online, and Google Scholar. The search focused on English language articles published between December 30, 2012, and January 1, 2023. We employed controlled vocabulary terms (MeSH) and free text to extract relevant articles. The search encompassed various databases such as PubMed, MEDLINE, HINARY, Africa Journals Online, and Google Scholar, and included topics such as active tuberculosis, pulmonary TB, extrapulmonary TB, HIV infection, individuals, children, pediatrics, neonates, lymphadenitis, disseminated TB, and Ethiopia. The search terms used to identify relevant studies included "Epidemiology" OR "Incidence" OR "Case fatality" AND "Tuberculosis" OR "Pulmonary Tuberculosis" OR "Disseminated Tuberculosis" OR "Lymphadenitis" AND "HIV" OR "AIDS" AND "Children" OR "Pediatrics" OR "Infant" AND "Ethiopia". We considered articles published between December 30, 2012, and January 1, 2023, and utilized cross-sectional, retrospective, and prospective cohort study designs in the search process. The selection criteria were predefined to identify the most relevant studies for the review.

~~**Methods**~~

~~Study Design~~

~~This systematic review and meta-analysis are designed to estimate the pooled burden of active TB among HIV-positive children living with HIV/AIDS in Ethiopia. Accordingly, we systematically reviewed published and unpublished articles from different databases. English language written articles were searched from five electronic databases which are published from December 30, 2012, to January 1st, 2023. We used PubMed, HINARY, WEB OF SCIENCE, Africa Journals Online, and Google Scholar databases for searching for articles using free text and controlled vocabulary terms (MeSH) to extract articles. The final PubMed search strategy included the following vocabulary terms (MeSH) to extract articles as shown in PubMed Article searching.~~

Study Design and Quality Assessment

Included articles in this study had cross-sectional, retrospective, and prospective cohort study designs published from December 30/2012 to January 1st, 2023**.** The PRISMA-2020(preferred Reporting Item for systematic reviews and Meta-Analysis standard was used to perform the frame of the whole review process for this study (**Supporting file =S1).** The quality of the included articles was assessed by three independent reviewers (FK, MM&TS) using Joana Brigg's Institute (JBI) critical appraisal checklist [33, 34]. We employed multiple checklists depending on the type of study, design of published publications, and the criteria to develop the final report into three categories: good, bad, and fair quality reviews. Any critical appraisal discrepancies among reviewers were resolved through discussion with the third-person reviewer (TK) ~~**(supporting file= S2).**~~

We used the preferred Reporting Item for systematic reviews and Meta-Analysis Protocols (PRISMA-2020) for this study[35]. Three independent reviewers assessed each included article's quality using the Newcastle-Ottawa Scale, and AHRQ standards (good, fair, and poor), and scales were given for all articles ~~**(supporting file =S2)**~~ [36, 37].

Inclusion criteria:

We included scientific papers that reported the following criteria for the final analysis

1. We included studies that reported TB and HIV co-infections among seropositive children in Ethiopia

2. We only considered cross-sectional and/or cohort studies as observational studies.

3. Articles containing prevalence or incidence reports of TB in HIV-positive children

4. A scientific paper published before January 1st, 2023with the study subjects only on children ≤15 years

Exclusion criteria

Articles without a journal name and /or author, conference proceedings, presentations, and reviews were excluded from the final meta-analysis.

Outcome ascertainment

The outcome variable for this review was the TB occurrence in HIV-positive children. We searched articles that measure this outcome. Before analysis, the log transformation of the odds

ratio was performed. A forest plot was generated to show the individual and pooled mortality proportions at 95%CI of the respective authors and years of publication.

Operational definition

Seroprevalence: All children ≤15 years having human immune deficiency virus through their blood started chronic HIV/AIDS care [38].

Prevalence: The number of new cases of TB divided by risk HIV-positive children after highly active antiretroviral therapy-initiated time with 100%.

Data synthesis and extraction

All citations identified by our search strategy, which were potentially eligible, were exported to End-Note-7 and removed the duplications. Titles and abstracts of articles were screened by three independent reviewers (FK, DT, and TK). The disagreement during the screening was resolved by 2/3 reviewer consensus. The full texts were then assessed by the same reviewers according to the specific criteria for final inclusion.

QUALITY ASSESSMENT AND APPRAISAL PROCEDURES

The data extraction and appraisal of each study quality were independently conducted by two authors (FK, and TK) for checked study titles and abstracts for eligibility after delating duplication. Any discrepancy or ambiguity during article extraction process was resolved by discussion. The full text articles were evaluated by same those reviewers if at least one of them thought an article has potentially importance, it was eligible. Two Authors (FK, and TK).) extracted details of each article information by using Microsoft-Excels spreadsheet after the paper were scrutinized for their titles, abstracts, and entire text. All studies approved by both authors agreements and any difference were worked out through, discussion to reached censuses.

Following the agreement, the information about principal investigators, years of publication, study periods, study setting, study population, and sample size was retrieved from the identified articles. Each of the included studies' risk of bias was assessed by all listed authors (FK, DT, and BN) investigators. The quality of the included articles was assessed using the New-Ottowa quality evaluation checklists was used to assessed the quality of the studies included in the final

meta-analysis. We employed multiple checklists depending on the type of study design of published paperes, and the criteria to develop the final report into three categories: good, bad, and fair quality result, and any critical appraisal discrepancies among reviewers were resolved through discussion with consusnce. Studies particiapnts, and setting, research design , recruiting technique, response rate, sample representativeness, valid measuring convention, measurement reliability, and proper statistical analysis are all included in the quality evaluation checklist.

DATA SYNTHESIS AND ANALYSIS PROCEDURES

All the identified and potentially eligible published articles citations were exported and collected using End- Note X8.1 and removed the duplications. After the titles abstracts of articles were screened by two independent reviewers (FK, and TK) and the full texts were then assessed by the same reviewers according to the specific criteria for final inclusion. We used STATA version `11 for further data analysis after the collected data were cleaned and modified on Microsoft-Excels spreadsheet. The outcome of the review was displayed using descriptive statistics. We used a random- effect model to assess the overall epidemiological survival patters and proportions of COVD-19 recover post admission as inpatients in Ethiopia, as well as statistically significant variable with their 95% CI's[39]. Meta-prop package was implemented to estimate the pooled proportion of recovery of COVDI-19 post hospitalized of cases in Ethiopia, and the pooled hazard ration of the associated predictors for time to recovery for COVDID-19 were evaluated from each article. The Higgs I^2 statistics was also utilized to detect heterogeneity. Heterogeneity between studies was elaborated using Cochran's Q test and quantified with the I^2 statistics. The magnitudes of statistical heterogeneity between studies were assessed using I^2 statistics and values of 25%, 50%, and 75% were considered to represent low, medium, and high respectively.

The subgroup analysis and sensitivity analyses were also used to investigation the source heterogeneity among the studies included. A P-value of ≥ 0.1 was considered to suggest statistically significant heterogeneity, considering the small number of studies. For further clarification on the source of heterogeneity, the random effect meta-regression was reported on the study setting, regions, and study population were done by subgroup analysis.

Heterogeneity and publication Biases assessment

Heterogeneity between studies was elaborated using Cochran's Q test and quantified with the I^2 statistics[40]. The magnitudes of statistical heterogeneity between studies were assessed using I^2

statistics and values of 25%, 50%, and 75% were considered to represent low, medium, and high respectively. A P-value of ≥ 0.1 was considered to suggest statistically significant heterogeneity, considering the small number of studies and their heterogeneity in the design[39]. For further clarification on the source of heterogeneity, the random effect meta-regression was reported on the study setting, regions, and study population by subgroup analysis. The publication biases were assessed by visual inspection of funnel plots and the graph suggested the absence of publication biases. For asymmetrical graphs during meta-analysis, we quantitatively assessed Egger's weighted regression at p <0.1[41, 42]. In addition, we performed a leave-one-out sensitivity analysis to confirm a study with a biased direction of pooled estimates of Begs and Eggers tests[39] as shown in supplementary file (S3).

Result

Included Studies screening

Overall, 1221 primary studies were identified, 43 from Web of Science, 631 from PubMed, 352 from Medline, 15 from Scopus, and the remaining 162 articles were extracted from Google Scholar. After the screening of duplication, 1208 articles were excluded through reading their titles and abstracts. The remaining 13 articles met the final inclusion criteria and included for reports (**Fig 1).** Thirteen (N=13) individual studies were included final analysis [1, 4, 23, 26, 27, 29, 43-49]. Seven of those articles were from Amhara [1, 26, 29, 43, 48, 49], three were from SNNR [27, 44, 46], two were from Benishangul Gumuz [4, 23], and one from Oromia[47] regions respectively. A total sample size of 5834 participants' children was included in the final analysis. The mean (±SD age of the participants was estimated at 8.2(±3.6) years. More than half (N=9/13, 69.2%) of studies recruited cohort design [23, 27, 29, 44, 46, 48, 49], and 38.8% of the included [4, 26, 43, 45, 48] papers used ≥ 10 years of follow-up (**Tabel 1).**

Descriptive of included studies

The majority (7/13= 53.8%) [1, 4, 23, 27, 29, 43, 47] of studies reported TB magnitudes, whereas the remaining four [26, 44, 48, 49] included crude incidence. The highest (23.6%) and the lowest (7.2%) TB burden were reported in the southern nations (SNNR) [27] and Amhara [26] regions, respectively. The final meta-analysis report indicated the pooled active TB

prevalence among children after ART was estimated to be 12.1% (95% CI: 10.7 - 13.4; I2 = 63.4%, p=0.001**Meta-analysis**

The majority (7/13= 53.8%) [1, 4, 23, 27, 29, 43, 47] of studies reported TB magnitudes, whereas the remaining four [26, 44, 48, 49] included crude incidence. The highest (23.6%) and the lowest (7.2%) TB burden were reported in the southern nations (SNNR) [27] and Amhara [26] regions, respectively. The final meta-analysis report indicated the pooled active TB prevalence among children after ART was estimated to be 12.1% (95% CI: 10.7 - 13.4; I2 = 63.4%, p=0.001) **(Fig 1).**

Sub-group-analysis

We conducted a subgroup analysis based on study regions and study settings to determine TB prevalence. In our subgroup analysis, TB prevalence among HIV-positive children in studies in Hospital setup was slightly lower than facility-based studies at 11.05% (95%CI: 9.4- 12.3 Vs. 14.1%; 95%CI: 11.74- 16.33). We also carried out a subgroup analysis based on the study regions of sampled pollution. Accordingly, the pooled TB prevalent in HIV-positive children initiated after ART in studies conducted in the Oromia region was significantly higher than studies conducted in 15.6% (95%CI:10.2- 20.6) as compared with studies conducted in the SNNPR region 12.8%(95%CI: 10.03- 15.67) **(Fig 2 and 3).**

Likewise, we conducted a subgroup analysis based on follow-up periods for the sampled population. The pooled TB prevalent in HIV-positive children after ART initiated with ≤10(120) years of the follow-up period were significantly higher than 13.67%(95%CI: 11.24 - 15.1) compared with a longer period' of follow-up time >10 years 10.9%(95%CI;9.1- 12.8) **(Fig4& 5)**.

Associated Risk factors

Adjusted odds ratios from primary studies were organized into three themes to identify risk factors for TB occurrence. Advanced clinical stage (WHO), baseline hemoglobin level, and isoniazid preventive therapy (IPT) were assessed. Because of that, advanced clinical stages and IPT were found to be statistically significant. Accordingly, the odds of tuberculosis occurrence among HIV-infected children who were in WHO clinical stages III and IV was 2.27 times more likely higher than those in WHO clinical stages I&II in a meta-analysis of four studies (OR= 2.27: 95%CI: 1.81-- 2.83, I^2 = 31.5%, P-value < 0.001) **(Figure 5 &6).**

The odds of developing TB among HIV-positive children for those who had baseline Hgb levels ≤10mg/dl was 4.26 times more likely than those having baseline Hgb >10mg/dl using 2371 participants from six studies (OR=4.26: 95CI: 3.47—5.24, I^2=43.3, P=0.001)

Sensitivity analysis

We performed a leave-one-out sensitivity analysis for the sake of potential sources of heterogeneity in the included studies for pooled TB prevalent as shown **in supporting file (S3).**

Publication bias

The publication bias was checked based on the symmetry of the funnel plots and quantitatively using Begg's and Egger's tests. We carried out Egger's regression and revealed the absence of publication bias with P=0.064. Nevertheless, checking out the funnel plot is slightly as symmetrical as shown in **(Fig 7).**

Discussion

In this meta-analysis, 13 individual studies with a total of 5834 participants were included. Among them, 834 HIV-positive children were found to have active tuberculosis (TB). The pooled prevalence of active TB in HIV-co-infected children, estimated during the meta-analysis, was 12.07% (95%CI: 10.73 - 13.4, I2 = 63.4%; P = 0.001).This finding is higher than previously reported in 0.78% in Ethiopia[50],43% in SSA countries [51], and 1.03 % in Portugal[52]. It is lower than the previous meta-analysis reported 15% in middle-income countries [53, 54]. The variation in pooled TB prevalence in Ethiopia at the national level may be attributed to differences in study time and variations among the included studies[55].

Conversely, the finding of this metanalysis report is lower than 2016 studies reported in 32% in Nigeria [56]. The lower TB prevalence in this Ethiopian meta-analysis compared to the 2016 study in Nigeria can be attributed to differences in study populations, settings, and TB control measures. The Ethiopian health ministry's efforts to improve TB control have likely contributed. Variations in study methodology, sample size, healthcare infrastructure, access to diagnostics, treatment practices, and regional differences also influence prevalence rates. Considering specific context and population characteristics is crucial when interpreting and comparing prevalence findings.

Discussion

This meta-analysis includes 5834 research participants from 13 included studies, including 834 reported active TB cases. During meta-analysis, the pooled TB prevalence in HIV-positive children was estimated at 12.07 % (95%CI; 10.73 - 13.4, I^2 =63.4%; P = 0.001). This finding is less comparable to the prior 0.78% finding in Ethiopia. [50],43% in SSA countries [51], and 1.03 % in Portugal[52]. It is lower than the previous meta-analysis reported 15% in middle-income countries [53, 54]. The possible reason for the variation could be attributed to study time, including participants, and notably diet-related factors. Subgroup analysis by study regions of the current study showed that the pooled TB prevalent among studies conducted in Oromia and SNNR regions has a significant variation. Which were 15.6%(95%CI:10.2- 20.6) Vs.12.8% (95%CI: 10.03- 15.67), respectively. This finding is lower than 2016 studies reported 32% in Nigeria [56]. This might be due to similar socioeconomic and using similar treatment and diagnosing guidelines for HIV-positive children. Whereas this is, this result is consistent with the 14.5% study reported in USEon 2023[57]. Likewise, we conducted a subgroup analysis based on follow-up periods for the sampled population. The pooled TB prevalent in HIV-positive children after ART initiated with ≤10(120 months) years of follow-up period were significantly higher than 13.67%(95%CI: 11.24 - 15.1) compared with a long period of follow-up time >10 years 10.9%(95%CI;9.1- 12.8).

Concerning the associated risk factors of TB, the final report of this meta-analysis showed that children in advanced clinical stages (III&IV) were at 2.27 times the increased risk of developing active TB in a meta-analysis of four studies (OR= 2.27: 95%CI: 1.81-- 2.83, I^2 = 31.5%, P= 0.001). This was supported by several meta-analyses reported in Ethiopia [58-61]. This might be because the advanced clinical stage of III&IV will have a low CD4 count and finally be unable to be defined with lethal opportunistic infection incidence during successive follow-up of care including TB [62].

This study findings also revealed that the risk of developing active TB was 4.26 (OR=4.26: 95CI: 3.47-- 5.25, I2=43.3, P=0.001) times greater in HIV-positive children with Hgb ≤10mg/dl than in those with Hgb >10mg/dl in the six included meta-analysis studies. This is consistent with the previous study reported [12, 63, 64]. This might be due to that anemia leads to the

development of infections including TB by impairing the function of hemoglobin levels and exposure indirectly to the incidence of opportunistic infection. Finally, efforts should be made to obtain a microbiologically confirmed TB case from suspected children to highlight their survival quality. Compared to microscopy, the Xpert MTB/RIF assay has better sensitivity for early diagnosing active TB in children, and its scale-up will improve access to tuberculosis diagnostics for children, as HIV-infected children are double risky for active TB.

Strengths and Limitations of the Study

The use of an extensive search strategy and clear inclusion criteria for the study population and the three independent authors' involvement in the quality assessment were among the top three strengths of this study. However, there are several methodological limitations to this analysis. First and most, there was a paucity of well-described TB reports among studies in HIV-positive children relative to the comparable findings in adult studies.

Conclusion and recommendation

Overestimation of active TB cases among HIV-positive children in Ethiopia was observed, with approximately one in ten affected. Risk factors for active TB included WHO clinical stages IV and III, as well as low hemoglobin levels. Effective management involves providing concurrent and continuous CPT (Cotrimoxazole preventive therapy), IPT (Isoniazid preventive therapy), and ART (antiretroviral therapy) for children living with HIV. Health professionals should adhere to screening and management guidelines, including isoniazid and co-trimoxazole prevention. Policymakers should enhance TB prevention strategies for at-risk children, and parents should prioritize feeding, adherence, and nutritional support for better outcomes.

~~Conclusions~~

~~A significant number of HIV-positive children suffered from twine (TB and HIV) infection in Ethiopia. During meta-analysis, the odds of tuberculosis were considerably greater in studies that included WHO clinical stages III&IV and Hgb 10mg/dl. The magnitudes of tuberculosis vary significantly across the study regions and follow-up times. As a result, strengthening laboratory services for early detection and treatment should be an absolute priority.~~

Recommendation

Special attention and efforts to reduce the burden of TB among HIV-positive children should be applied in Ethiopia. Health professionals working with HIV-positive children should routinely

screen and manage opportunistic infections based on the recommended guideline and address isoniazid and co-trometamol preventive therapy during consecutive follow-ups. Moreover, policymakers should incorporate early prevention strategies for TB by enhancing screening and treatment principles for risky children. Parents of HIV-positive children should improve their feeding, drug adherence, and nutritional support to prolong survival outcomes

Abbreviation

TB: Tuberculosis, WHO; whorl health organization, FOM; federal ministry of Health, HIV; human immune deficiency varies, HAAR: highly active antiretroviral therapy, WHO; Whorl Health Organization, IPT, isoniazid preventive Therapy CPT, co-trimoxazole preventive therapy OIS; opportunistic infection

Acknowledgment

We would like to thank Woldia University College of Health Science Research and Community Service for their support of met analysis software training

Authors contribution

FK designed the study, conducted part of the search strategy and study selection, conducted the meta-analysis and data synthesis, and wrote the manuscript. MM, TK, and TS have contributed to the search strategy, article selection, and report writing and final revised full manuscript. Finally, all authors read, commence, and approve for the final revised submissions.

Funding

There is no specific funding for this research

Declaration

Ethics approval and consent to participate

Not applicable for this report

Consent for publication

Not applicable for this report

Computing interest

The authors declared that they have no conflict of interest

Data availability

Not applicable

Supplementary information

S1= PRISMA checklist 2020 (DOC)

S2=JBI supporting files

Reference

[1] S.G. Ayalaw, K.A. Alene, A.A. Adane, Incidence and Predictors of Tuberculosis among HIV Positive Children at University of Gondar Referral Hospital, Northwest Ethiopia: A Retrospective Follow-Up Study, Int Sch Res Notices 2015 (2015) 307810.
[2] A. Alemu, A. Yesuf, E. Gebrehanna, B. Zerihun, M. Getu, T. Worku, Z.W. Bitew, Incidence and predictors of extrapulmonary tuberculosis among people living with Human Immunodeficiency Virus in Addis Ababa, Ethiopia: A retrospective cohort study, PLoS One 15(5) (2020) e0232426.
[3] F. Kebede, B. Kebede, T. Kebede, M. Agmasu, Effect of Isoniazid Preventive Therapy on the Incidence of Tuberculosis among Seropositive Children Attending HIV/AIDS Care in Two General Hospitals, Northwest Ethiopia, 2021, Journal of Tropical Medicine 2021 (2021) 9996953.
[4] F. Kebede, H. Tarekegn, M. Molla, D. Jara, A. Abate, Incidence and Predictors of Pulmonary Tuberculosis among Children Who Received Antiretroviral Therapy (ART), Northwest Ethiopia: A Multicenter Historical Cohorts Study 2009-2019, Journal of tropical medicine 2022 (2022) 9925693.
[5] F. Kebede, B. Kebede, T. Kebede, M. Agmasu, Effect of Isoniazid Preventive Therapy on the Incidence of Tuberculosis among Seropositive Children Attending HIV/AIDS Care in Two General Hospitals, Northwest Ethiopia, 2021, Journal of tropical medicine 2021 (2021) 9996953.
[6] W.W. 1, L. Dube2, T.K. 2, Factors Affecting Survival Rates Among Adult TB/ HIV Co-Infected Patients in Mizan Tepi University Teaching Hospital, South West Ethiopia, HIV/AIDS - Research and Palliative Care 2020:12 157–164 (2020).
[7] Animut Alebel1, Eshetu Haileselassie Engeda3, Mengistu Mekonnen Kelkay3, Pammla Petrucka4,5,, G.D. Kibretett.al, Mortality rate among HIV-positive children on ART in Northwest Ethiopia: a historical cohort study, BMC Public Health 20:1303 https://doi.org/10.1186/s12889-020-09418-6 (2020).
[8] M.K. Jemberu Nigussie1Jemberu Nigussie1, Goitom Halefom3 Haven Hadush2 Mekuria Kassa, Goitom Halefom, Haven Hadush,, B.G.y.a.B. Grima1, Predictors of Mortality Among Children Co-Infected with Tuberculosis and Human Immunodeficiency Virus in Region, North Ethiopia, Retrospective Follow-Up Study, med J Sci & Tech Res | BJSTR. MS.ID.006252. ISSN: 2574 -1241(DOI: 10.26717/BJSTR.2021.39.006252) (2021).
[9] Y.Z. Weiwei Mua, Xin Suna, Ye Maa, Lan Yua, Xia Liua, Decai Zhaoa, Zhihui Doua, Hua, F.F. Zhangab, Incidence and associated factors of pulmonary tuberculosis in HIV-infected children after highly active

antiretroviral therapy (HAART) in China: a retrospective study, AIDS Care: Psychological and Socio-medical Aspects of

AIDS/HIV Psychological and Socio-medical Aspects of AIDS/HIV, 26:9,

1127-1135, DOI: 10.1080/09540121.2014.882492 (2015,).
[10] C. Schutz, D. Barr, B.B. Andrade, M. Shey, A. Ward, S. Janssen, R. Burton, K.A. Wilkinson, B. Sossen, K.F. Fukutani, M. Nicol, G. Maartens, R.J. Wilkinson, G. Meintjes, Clinical, microbiologic, and immunologic determinants of mortality in hospitalized patients with HIV-associated tuberculosis: A prospective cohort study, PLoS medicine 16(7) (2019) e1002840.
[11] G. Marley, X. Zou, J. Nie, W. Cheng, Y. Xie, H. Liao, Y. Wang, Y. Tao, J.D. Tucker, S. Sylvia, R. Chou, D. Wu, J. Ong, W. Tang, Improving cascade outcomes for active TB: A global systematic review and meta-analysis of TB interventions, PLoS medicine 20(1) (2023) e1004091.
[12] G.M. Belay, C.A. Wubneh, Childhood tuberculosis treatment outcome and its association with HIV co-infection in Ethiopia: a systematic review and meta-analysis, Tropical Medicine and Health 48(1) (2020) 7.
[13] WHO, GLOBAL TUBERCULOSIS REPORT 2020, Report accesed 10 september (2020).
[14] C. Marotta, M. Abdu, Y. Ali, S. Anteneh, M. Yesuf, A. Birhanu, S. Mohamed, A. Hussien, Determinant factors for the occurrence of tuberculosis after initiation of antiretroviral treatment among adult patients living with HIV at Dessie Referral Hospital, South Wollo, Northeast Ethiopia, 2020. A case-control study, Plos One 16(3) (2021) e0248490.
[15] WHO, GLOBAL TUBERCULOSIS REPORT 2019, ACCESED SEPTEMEBR 10 ISBN 978-92-4-156571-4 (2019).
[16] S.A. Zinabu DawitID1*, Samuel DessuID3, Molalegn Meseleet.al, Incidence and predictors of mortality among children co-infected with tuberculosis and human immunodeficiency virus at publichospitals in Southern Ethiopia, PloS ONE: e0253449. https://doi.org/10.1371/journal.pone.0253449 (2021).
[17] FMOH, NATIONALCONSOLIDATED GUIDELINES FOR COMPREHENSIVE HIV PREVENTION, CARE AND TREATMENT, Manuel Accesed septemebr 2021 (2018).
[18] FMOH, Ethiopia-National guidelines for TB, DR-TB and Leprosy in Ethiopia - Sixth Edition, Manuale ACCESSED 2021 (2017).
[19] A.M.H.T. Mohammedaman Mama, Hawa Mohammed and Endeshaw Erbo, Prevalence of Pulmonary Tuberculosis and Associated Factors Among HIV Positive Patients Attending Antiretroviral Therapy Clinic at Arba Minch General Hospital, Southern Ethiopia, The Open Microbiology Journal, 12, 163-171 12, 163-171(DOI: 10.2174/1874285801812010163) (2018).
[20] T. Kiros, E. Dejen, M. Tiruneh, T. Tiruneh, T. Eyayu, S. Damtie, K. Amogne, Magnitude and Associated Factors of Pulmonary Tuberculosis Among HIV/AIDS Patients Attending Antiretroviral Therapy Clinic at Debre Tabor Specialized Hospital, Northwest Ethiopia, 2019, HIV AIDS (Auckl) 12 (2020) 849-858.
[21] M. Abdu, Y. Ali, S. Anteneh, M. Yesuf, A. Birhanu, S. Mohamed, A. Hussien, Determinant factors for the occurrence of tuberculosis after initiation of antiretroviral treatment among adult patients living with HIV at Dessie Referral Hospital, South Wollo, Northeast Ethiopia, 2020. A case-control study, PLoS One 16(3) (2021) e0248490.
[22] J.U.N.P.o.H.A. (UNAIDS, UNAIDS Global AIDS Update 2022, UNAIDS GLOBAL AIDS UPDATE (2022).
[23] F. Kebede, T. Kebede, B. Kebede, A. Abate, D. Jara, B. Negese, T. Shaweno, Time to Develop and Predictors for Incidence of Tuberculosis among Children Receiving Antiretroviral Therapy, Tuberc Res Treat 2021 (2021) 6686019.

[24] Mehretu Belayneha, Kalayta Gidayb, Hailemariam Lemmac, Treatment outcome of human immunodeficiency virus and tuberculosis co-infected patients in public hospitals of eastern and southern zone of

Tigray region, Ethiopia, b r a z j i n f e c t d i s . 2 0 1 5;1 9(1):47–51 (http://dx.doi.org/10.1016/j.bjid.2014.09.00) (2014).
[25] D.S.a.A. Worku2, Tuberculosis treatment survival of HIV positive TB patients on directly observed treatment short-course in Southern Ethiopia: A retrospective

cohort study, BMC Research Notes 2012, 5:682 http://www.biomedcentral.com/1756-0500/5/682 (2012).
[26] E.S. Chanie, W.A. Bayih, B.M. Birhan, D.M. Belay, G. Asmare, T. Tiruneh, Y.A.A. Aynalem, B.B. Abat, S. Asnakew, M. Mekie, G.Y. Yitbarek, F.A. GebreEyesus, Incidence of advanced opportunistic infection and its predictors among HIV infected children at Debre Tabor referral Hospital and University of Gondar Compressive specialized hospitals, Northwest Ethiopia, 2020: A multicenter retrospective follow-up study, Heliyon 7(4) (2021) e06745.
[27] F.T.Y. Deyas, Effect of highly active antiretroviral treatment on TB incidence among HIV infected children and their clinical profile, retrospective cohort study, Sout West Ethiopia, Scientific Reports 10:21468 https://doi.org/10.1038/s41598-020-78466-0 (2020).
[28] D. Tsegaye, S. Wude, T. Kebede, S. Adane, T. Shumet, F. Kebede, Epidemiological survival pattern, risk factors, and estimated time to develop tuberculosis after test and treat strategies declared for children living with human immune deficiency virus, Indian Journal of Tuberculosis (2023).
[29] Y.M. Alemu, G. Andargie, E. Gebeye, High Incidence of Tuberculosis in the Absence of Isoniazid and Cotrimoxazole Preventive Therapy in Children Living with HIV in Northern Ethiopia: A Retrospective Follow-Up Study, PLoS One 11(4) (2016) e0152941.
[30] M.T. Beshir, A.H. Beyene, K.G. Tlaye, T.M. Demelew, Incidence and predictors of tuberculosis among HIV-positive children at Adama Referral Hospital and Medical College, Oromia, Ethiopia: a retrospective follow-up study, Epidemiol Health 41 (2019) e2019028.
[31] F.T.Y. Deyas, Effect of highly active antiretroviral treatment on TB incidence among HIV infected children and their clinical profile, retrospective cohort study, South West Ethiopia, Scientific Reports 10:21468 https://doi.org/10.1038/s41598-020-78466-0 (2020).
[32] Endalk Birrie Wondifraw a, Ermias Sisay Chanie b, FishaAlebel Gebreeyesus c, Gebeyaw Biset a, Birhanu Desu Tefera d, Mulusew Zeleke e, Incidence and predictors of tuberculosis among children on antiretroviral therapy at northeast Ethiopia comprehensive specialized hospitals, 2022; A multicenter retrospective follow-up study, Heliyon 8 (2022) e12001 Heliyon 8 (2022) e12001 (2022).
[33] JBI, JBI_Critical_Appraisal-Checklist_for_Analytical_Cross_Sectional_Studies http://joannabriggs.org/research/critical-appraisal-tools.html www.joannabriggs.org (2017).
[34] JNI, The Joanna Briggs Institute Critical Appraisal tools for use in JBI Systematic Reviews, http://joannabriggs.org/research/critical-appraisal-tools.html www.joannabriggs.org (2017).
[35] P. checklist, PRISMA_2020_expanded_checklist for systemic reviwe and meta-analysis studies reporting BMJ 2021;372:n16 BMJ 2021;372:n71. doi: 10.1136/bmj.n71 (2021).
[36] Professor GA Wells, NEWCASTLE-OTTAWA QUALITY ASSESSMENT SCALE COHORT STUDIESf>, J Educ Eval Health Prof 2016; 13: 40 • https://doi.org/10.3352/jeehp.2016.13.40 (2016).
[37] J.B. Institute, JBI_Critical_Appraisal-Checklist_for_Analytical_Cross_Sectional_Studies, Checklist for Analytical Cross Sectional Studies, http://joannabriggs.org/research/critical-appraisal-tools.html www.joannabriggs.org (2017).

Field Code Changed

Field Code Changed

Field Code Changed

[38] FMOH, Guidelines_for_Clinical_and_Programmatic_Management_of_TB_TBHIV., Guideline (7th edition) (2021).
[39] M. Egger, G. Davey Smith, M. Schneider, C. Minder, Bias in meta-analysis detected by a simple, graphical test, BMJ 315(7109) (1997) 629-634.
[40] S.G. Thompson, J.P. Higgins, How should meta-regression analyses be undertaken and interpreted?, Statistics in medicine 21(11) (2002) 1559-73.
[41] M. Borenstein, L.V. Hedges, J.P. Higgins, H.R. Rothstein, A basic introduction to fixed-effect and random-effects models for meta-analysis, Research synthesis methods 1(2) (2010) 97-111.
[42] S. Duval, R. Tweedie, A Nonparametric "Trim and Fill" Method of Accounting for Publication Bias in Meta-Analysis, Journal of the American Statistical Association 95(449) (2000) 89-98.
[43] E.H.E.a.N.T. Aklilu Endalamaw*, Incidence of tuberculosis in children on antiretroviral therapy: a retrospective cohort study, BMC Res Notes 11:745 https://doi.org/10.1186/s13104-018-3846-z (2018).
[44] D.J. Emil Westerlund, Zewdie Mulissa Inger Hallström and Bernt Lindtjørn5, Pre-ART retention in care and prevalence of tuberculosis among HIV-infected children at a district hospital in southern Ethiopia, BMC pediatrics 14:250 http://www.biomedcentral.com/1471-2431/14/250 (2014).
[45] E.S.C. Endalk B, Fisha A.G, Gebeyaw Biset et.al Incidence and predictors of tuberculosis among children on antiretroviral therapy at northeast Ethiopia comprehensive specialized hospitals, 2022; A multicenter retrospective follow-up study>, Heliyon 8 (2022) e12001 https://doi.org/10.1016/j.heliyon.2022.e12001 (2022).
[46] Y.D.a.D.A. Firew Tiruneh*, tuberculosis-incidence-among-hiv-infected-children-on-haart-and-their-clinical-profile-retrospective-cohort-study-south-, Journal of AIDS & Clinical Research Research Article Volume 11:3,2020 DOI: 10.37421/jar.2020.11.808 (2020).
[47] T.M.D. Masino Tessu Beshir1 Aklil Hailu Beyene2 Kenean Getaneh Tlaye3, Incidence and predictors of tuberculosis among HIV- positive children at Adama Referral Hospital and Medical College, Oromia, Ethiopia: a retrospective follow-up study, epih.org 41, Article ID: e2019028, 8 (https://doi.org/10.4178/epih.e2019028) (2017).
[48] M.W. Melkamu, M.T. Gebeyehu, A.D. Afenigus, Y.T. Hibstie, B. Temesgen, P. Petrucka, A. Alebel, Incidence of common opportunistic infections among HIV-infected children on ART at Debre Markos referral hospital, Northwest Ethiopia: a retrospective cohort study, BMC infectious diseases 20(1) (2020) 50.
[49] D.A. Mequanente, P. Srinivasan, G. Mallika, P.M.T. Ansari, M. Wale, Incidence of Opportunistic Infections among HIV-infected Children on ART at Gondar University Specialized Hospital, Ethiopia, Indian Journal Of Science And Technology 15(34) (2022) 1675-1682.
[50] B. Solomon M. Abay, MSc, PhD1, Kebede Deribe, BSc, MPH2,3,, B. Ayalu A. Reda, MSc4, Sibhatu Biadgilign, BSc, MPH5, The Effect of Early Initiation of Antiretroviral Therapy in TB/HIV-Coinfected Patients: A Systematic Review and Meta-Analysis, Journal of the International Association of Providers of AIDS Care 2015, Vol. 14(6) 560–570 DOI: 10.1177/2325957415599210 (2015).
[51] G. Diriba, A. Alemu, K. Eshetu, B. Yenew, D.F. Gamtesa, H.H. Tola, Bacteriologically confirmed extrapulmonary tuberculosis and the associated risk factors among extrapulmonary tuberculosis suspected patients in Ethiopia: A systematic review and meta-analysis, PloS one 17(11) (2022) e0276701.
[52] I.F. Ana Maria Tavares*, Isabel Couto, Diana Machado, Miguel Viveiros, AnaB. Abecasis, So, n. Dias, HIV and tuberculosis co-infection among migrants in Europe: A systematic review on the prevalence, incidence and mortalit, PLoS ONE 12(9):e0185526. https://doi.org/10.1371/journal.pone.0185526 (2017).

[53] L. Pimpin, L.N. Drumright, M.E. Kruijshaar, I. Abubakar, B. Rice, V. Delpech, V. Hollo, A. Amato-Gauci, D. Manissero, C. Ködmön, Tuberculosis and HIV co-infection in European Union and European Economic Area countries, European Respiratory Journal 38(6) (2011) 1382.
[54] M.-R. B-Lajoie, O. Drouin, G. Bartlett, Q. Nguyen, A. Low, G. Gavriilidis, P. Easterbrook, L. Muhe, Incidence and Prevalence of Opportunistic and Other Infections and the Impact of Antiretroviral Therapy Among HIV-infected Children in Low- and Middle-income Countries: A Systematic Review and Meta-analysis, Clinical Infectious Diseases 62(12) (2016) 1586-1594.
[55] I. Law, K. Floyd, T.B.P.S.G. the African, National tuberculosis prevalence surveys in Africa, 2008–2016: an overview of results and lessons learned, Tropical Medicine & International Health 25(11) (2020) 1308-1327.
[56] Augustine O. Ebonyi1, A.S. Sagay7, E.U.E. , P.O.U. , P.I.O. , Prevalence of and risk factors for pulmonary tuberculosis among newly diagnosed HIV-1 infected Nigerian children, GERMS 6(1) • March 2016 • page 21 (2016).
[57] L. Powell, L. Denoeud-Ndam, N. Herrera, R. Masaba, B. Tchounga, S. Siamba, M. Ouma, S.J. Petnga, R. Machekano, B. Pamen, G. Okomo, L. Simo, M. Casenghi, N. Rakhmanina, A. Tiam, HIV matters when diagnosing TB in young children: an ancillary analysis in children enrolled in the INPUT stepped wedge cluster randomized study, BMC infectious diseases 23(1) (2023) 234.
[58] Animut Alebel1, Daniel Demant2,3, Pammla Petrucka4,5 and David Sibbritt2, Effects of undernutrition on mortality and morbidity among adults living with HIV in sub-Saharan Africa: a systematic review and meta-analysis, BMC Infectious Diseases (2021) 21:1 https://doi.org/10.1186/s12879-020-05706-z (2021).
[59] N.M.D. Melkalem Mamuye Azanaw1, Gebeyehu Shumuye Yetemegn2 and, D.A. Angaw3*, Incidence and predictors of tuberculosis among HIV patients after initiation of antiretroviral treatment in Ethiopia: a systematic review and meta-analysis, Tropical Medicine and Health 49:18(https://doi.org/10.1186/s41182-021-00306-2) (2021).
[60] M. Straetemans, P. Glaziou, A.L. Bierrenbach, C. Sismanidis, M.J. van der Werf, Assessing tuberculosis case fatality ratio: a meta-analysis, PLoS One 6(6) (2011) e20755.
[61] A.G.-W. Marriott Nliwasa Peter MacPherson4, 3, Mphatso Mwapasa1, Katherine Horton3,, Jon Ø Odland5, Clare Flach7 and Elizabeth L. Corbett1,, High HIV and active tuberculosis prevalence and increased mortality risk in adults with symptoms of TB: a systematic review and meta-analyses, Journal of the International AIDS Society 2018, 21:e25162 https://doi.org/10.1002/jia2.25162 (2018).
[62] S. Wude, D. Tsegaye, S. Adane, T. Kebede, F. Kebede, T. Shumet, Epidemiological Survival Pattern, Risk Factors, And Estimated Time To Develop Tuberculosis After Test And Treat Strategies Declared For Children Living With Human Immune Deficiency Virus, Indian Journal of Tuberculosis (2023).
[63] B. Tesfaye, A. Alebel, A. Gebrie, A. Zegeye, C. Tesema, B. Kassie, The twin epidemics: Prevalence of TB/HIV co-infection and its associated factors in Ethiopia; A systematic review and meta-analysis, PLoS One 13(10) (2018) e0203986.
[64] N.M.D. Melkalem Mamuye Azanaw1, Gebeyehu Shumuye Yetemegn2 and Dessie Abebaw Angaw, Incidence and predictors of tuberculosis among HIV patients after initiation of antiretroviral treatment in Ethiopia: a systematic review and meta-analysis, Tropical medicine and health 49:18(https://doi.org/10.1186/s41182-021-00306-2) (2021).

Table 1: General characteristics of the included articles/studies with finally describing reports of the prevalence of TB in HIV-positive children in Ethiopia after ART was started

Author	Year	Region	Design	Sample size	Mean age	Events	Prevalence	Incidence/100 PPY	Follow up time	Study setting	Quality
**Muluget et.al [29]**	2016	Amhara	Follow up	647	6	79	12.2	4.2	5	HT&HC	3
**Kebede et . al [23]**	2021	Benishangul	Cohort	421	8	52	12.4	5.9	5	HT	3
**Tiruneh et.al [27]**	2020	SNNRS	cohort	800	9	189	23.6	7.9	5	HT&HC	3
**Kebede et.al [4]**	2021	Benishangul	Cohort	428	10.3	64	14.9	5.78	10	HT&HC	3
**Tiruneh et.al [46]**	2020	SNNRS	Cohort	844	9	113	13.4	3.36	5	HT&HC	3
**Endalamaw et.al[43]**	2018	Amhara	Cohort	352	6.7	34	9.6	2.63	13	HT	3
**Tessu et.al [47]**	2019	Oromia	Cohort	428	6	67	15.6	6.03	5	HT	3
**Gebeyehu et.al [1]**	2015	Amhara	Follow up	271	9.8	44	16.2	4.9	6	HT	3
**Wubale et.al [48]**	2020	Amhara	Cohort	408	6.3	42	10.3	------	15	HT&HC	3
**Sissay et.al [26]**	2022	Amhara	Follow up	349	7.3	25	7.2	-------	11	HT	3
**Mequanente et.al [49]**	2022	Amhara	Cohort	389	7.9	57	10.5	--------	6	HT	3
**Endalk et.al [45]**	2022	Amhara	Follow up	358	8.3	57	15.9	2.0	14	HT	3
**Westerlund et.al [44]**	2014	Arba Minch	Cohort	139	5.9	11	7.9	-------	6	HT	2

* HC= Health center, HT= hospital

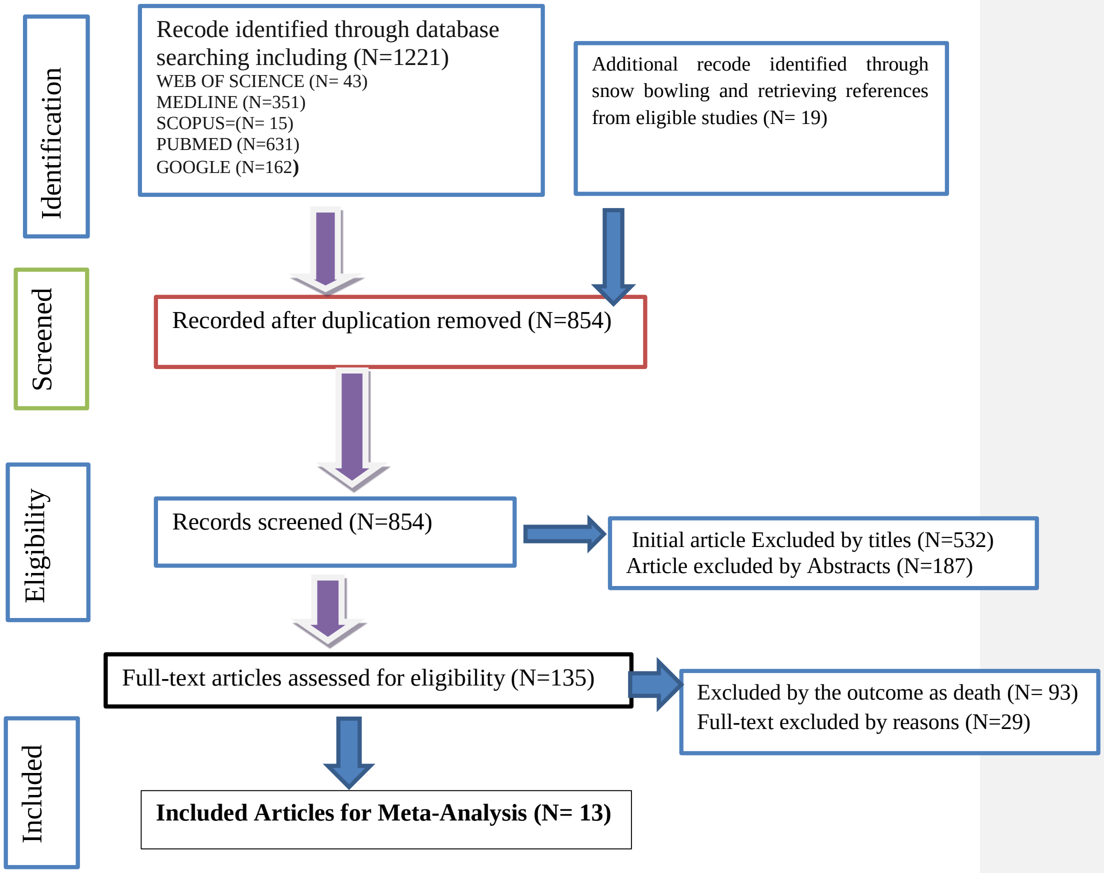

Figure 1: A PRISMA flow diagram for describing the flow chart of selected articles eligible for reviewing

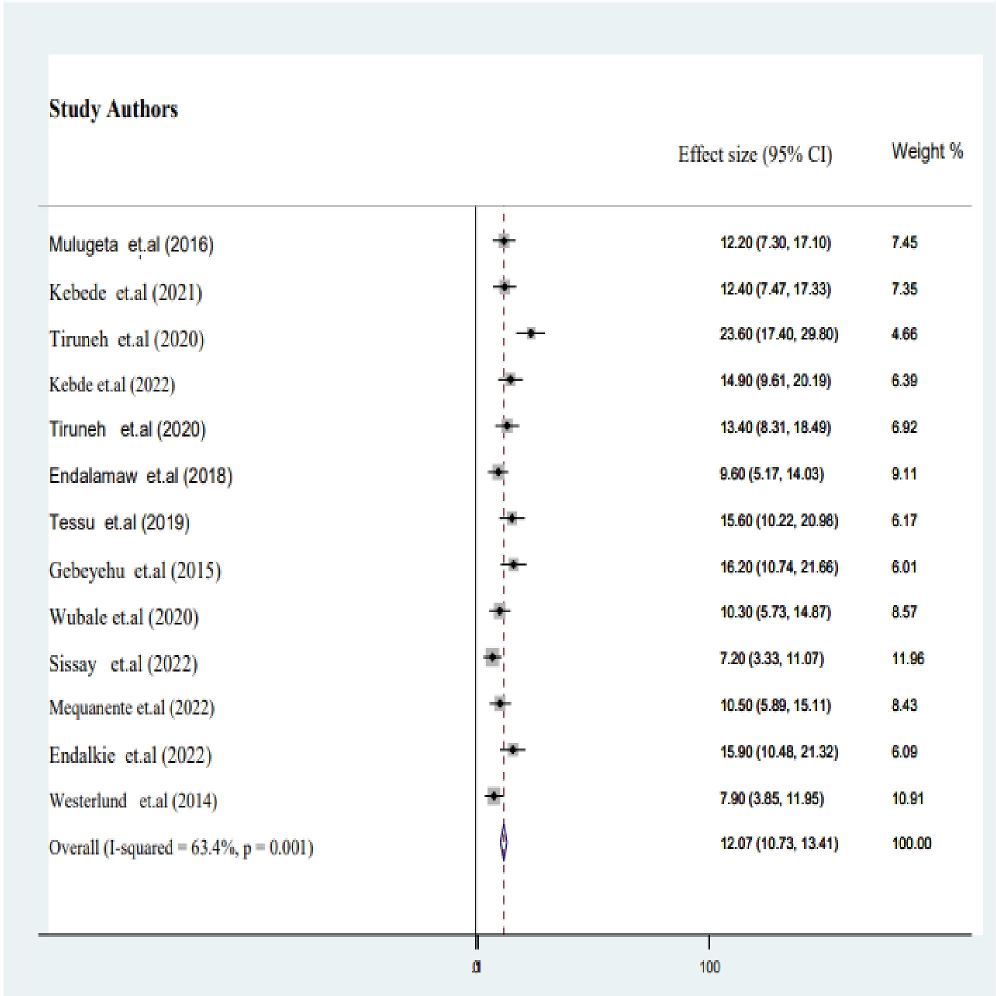

Fig2. Forest plot of the pooled prevalence of active TB in HIV- infected children in Ethiopia

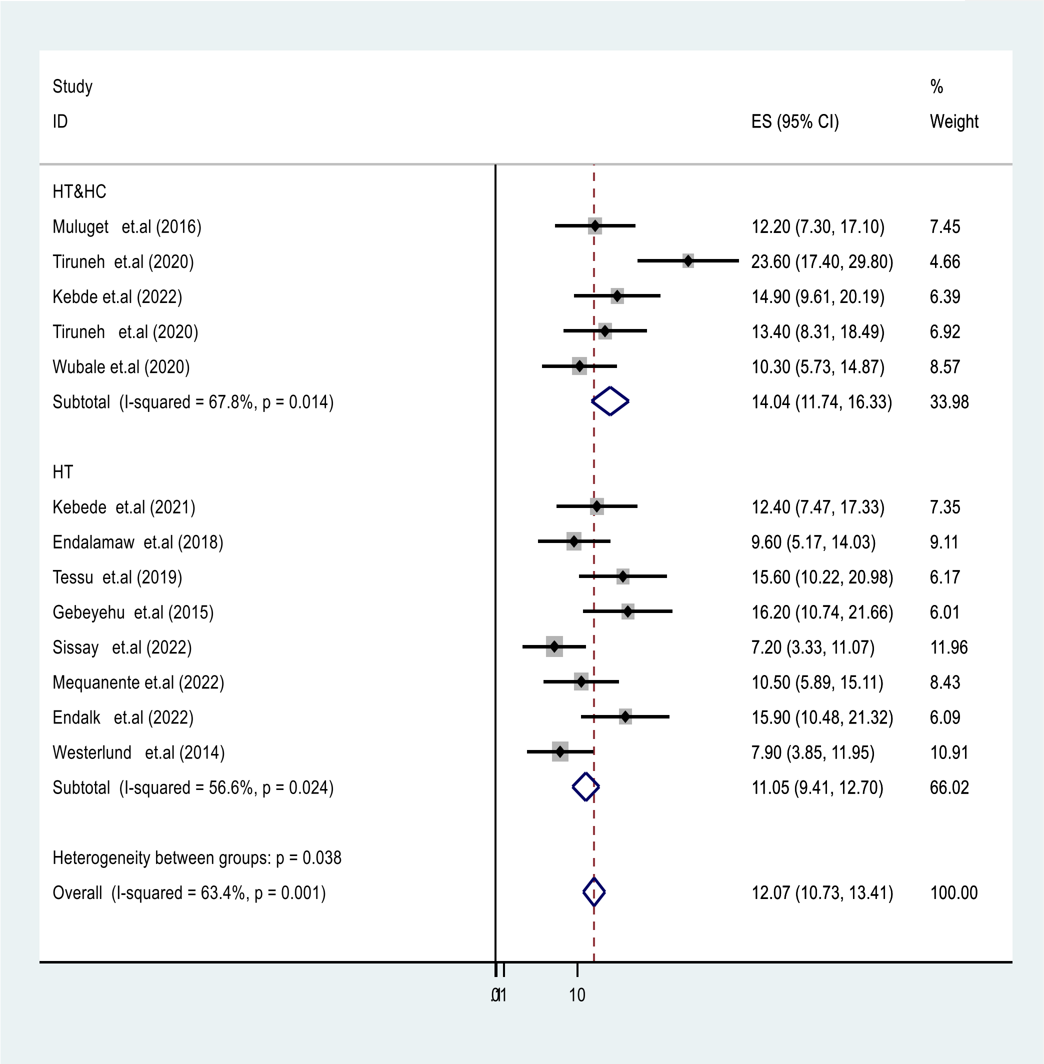

***HC**= health center; HT=Hospital

Fig 3. **Forest plot for subgroup analysis of active TB prevalence among HIV infected children stratified by study setting**

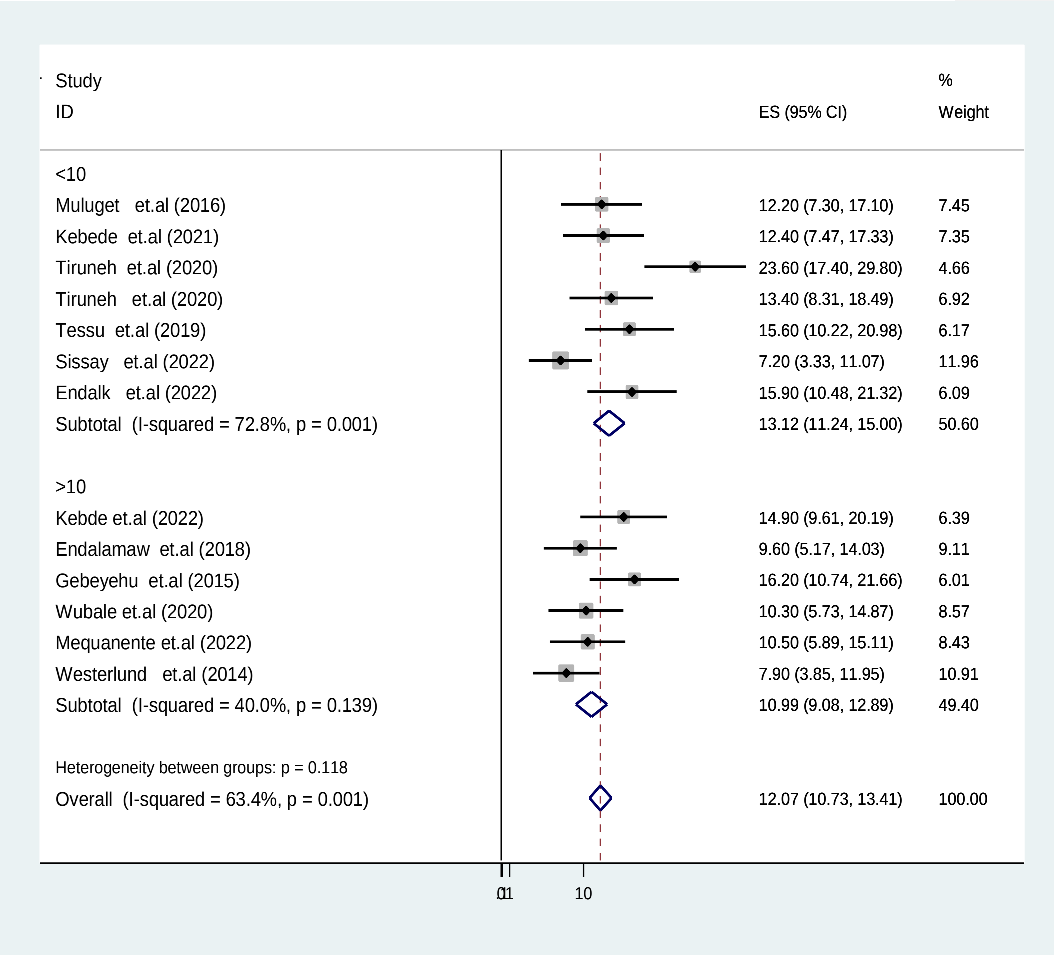

Fig4.**Forest plot for subgroup analysis of active TB prevalence in HIV-infected children stratified by follow periods**

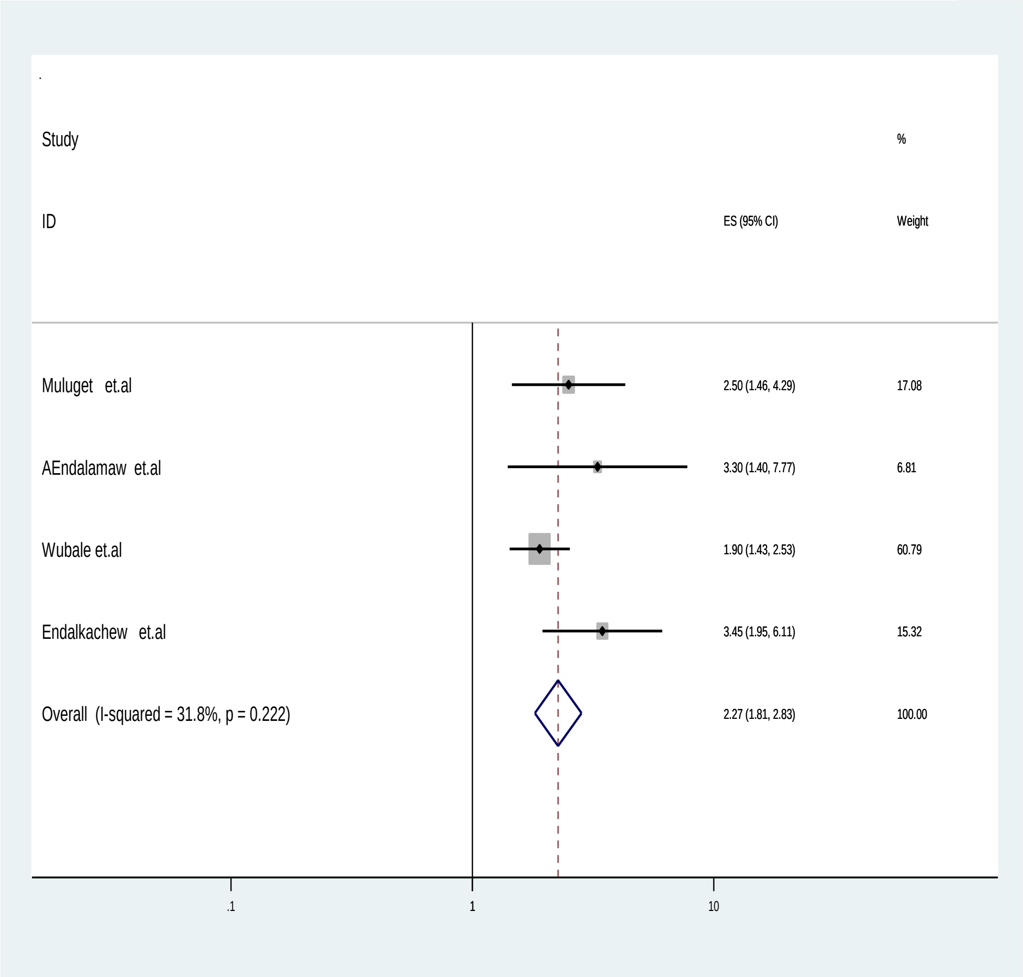

Fig 5.The pooled prevalence of active TB in HIV-infected children among studies identified by isoniazid

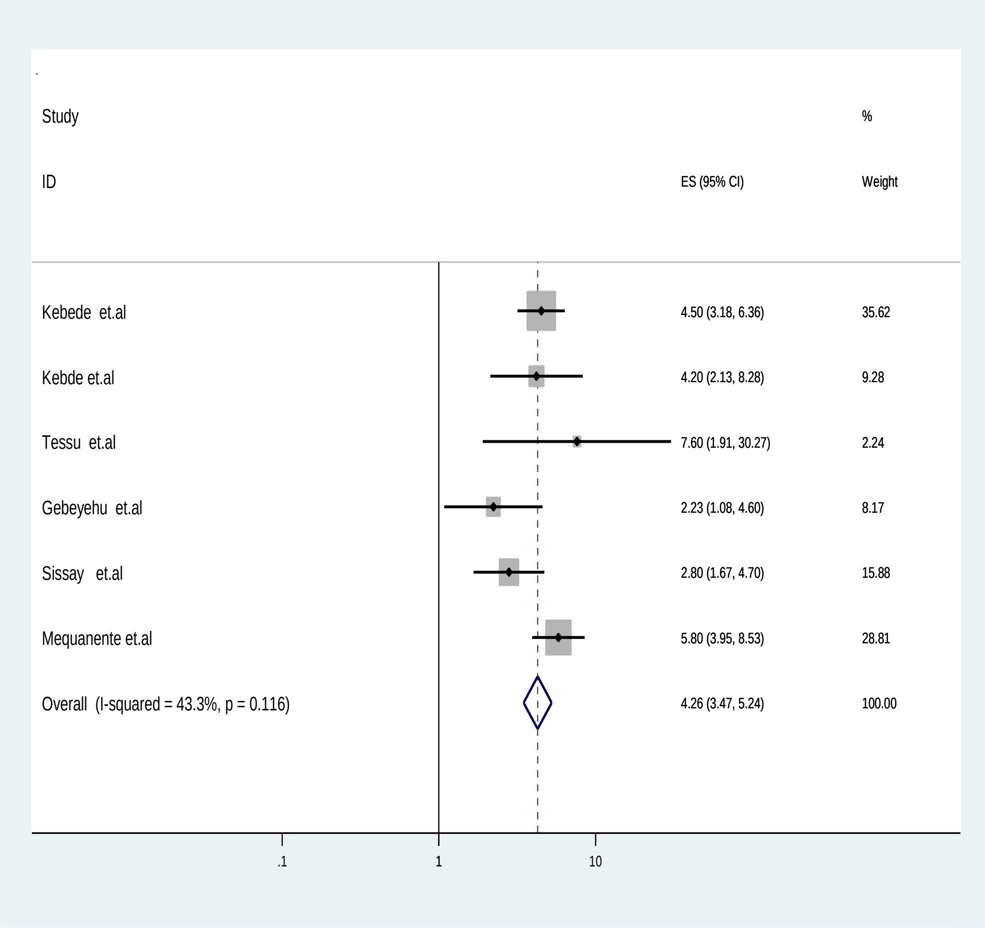

Fig6. The pooled prevalence of active TB in HIV-infected children among studies identified by Co-trimoxazole

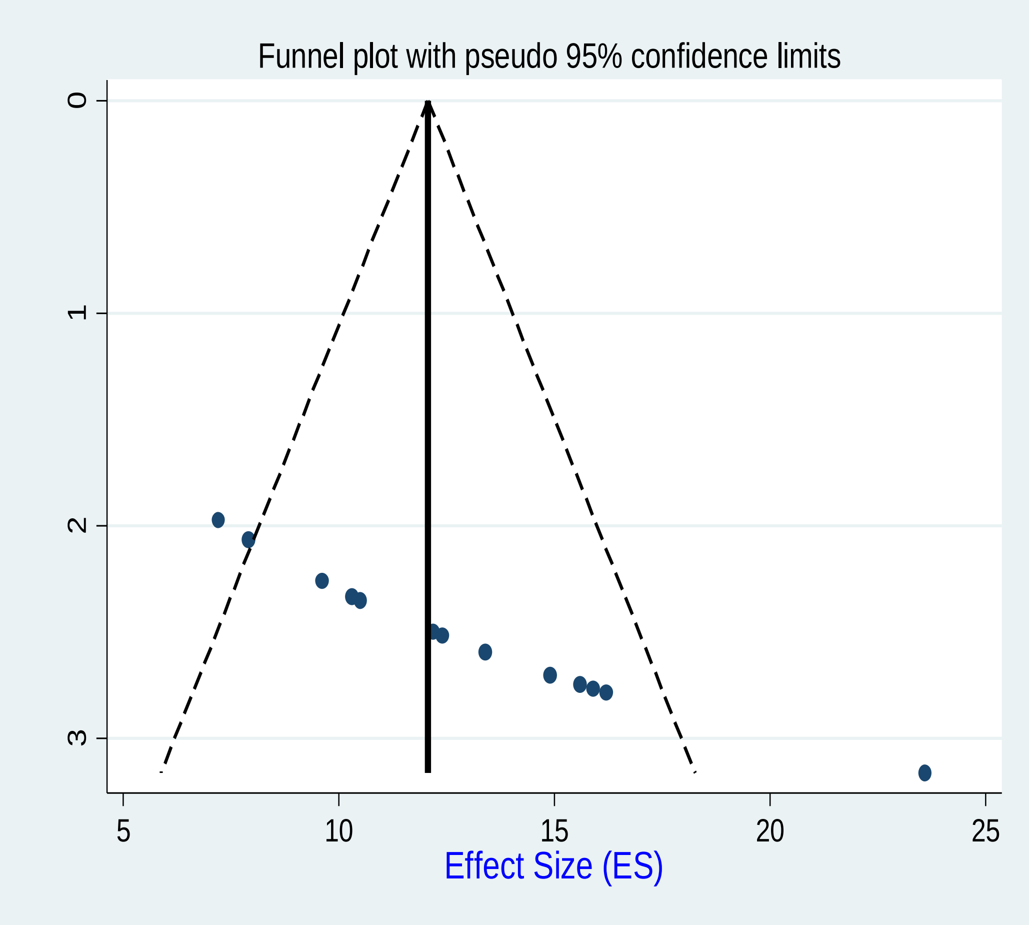

Fig 7: Funnel plot for the pooled prevalence of TB cases amongst all studies identified for review in Ethiopia

Authors response for reviewers' comments

Version =2
Number of authors= 5
Revised tittles:

Meta-Analysis of Active Tuberculosis Occurrence among Children Living with HIV Post Anti-Retroviral Therapy Initiated in Ethiopia

Editor's comment

#1. Please provide a complete some minor occurrence of overlapping text with the following previous publication(s), which needs to be addressed: https://doi.org/10.1155/2022/9925693

Response: Thanks for your comments and we clarified all text overlap with previous manuscript and clear, edited, and the introduction of this manuscript cleared it read it again

#2 Please provide separate figure files in .tif or .eps format only and remove any figures embedded in your manuscript file. Please also ensure all files are under our size limit of 10MB. Based on PLOS Global public Health Journals manuscript Figure presentation Guideline

Responses: Thanks for your comment we have amended and wrong reference styles and make as if the PLOS Global journals submission guide, we had submitted as if it. please read it :

Reviewer #1:

1. Reviewer #1: All comments given in attachment. Q1 and Q2 - main concerns - there may be one duplicate study population - researchers need to verify that this is not the case. Incidence data analysis incomplete. The rest of the analyses is done fairly comprehensively and appropriately. Q3. I could not find the corresponding data file in supplementary files - although the pdf manuscript states that the data was provided.

# Responses; Thanks for your comments and feedback on our manuscript titled "Meta-Analysis of Active Tuberculosis Occurrence among Children Living with HIV Post Anti-Retroviral Therapy Initiated in Ethiopia." We have addressed your concerns regarding duplicate study population and incomplete incidence data analysis by carefully reviewing our data and methods. We have verified the uniqueness of each participant included in the meta-analysis and provided additional comprehensive details on the incidence data analysis in the revised manuscript. We apologize for the missing data file in the supplementary files and have rectified this issue by including the complete and relevant data file for

transparency and reproducibility. Thank you for bringing this to our attention, and we assure you that the data file is now available for review.

For your question of Please find our responses to your comments for

Q1 and Q2 question how could you managed duplication and missing data

We acknowledge your concerns regarding the possibility of a duplicate study population and the incomplete analysis of incidence data. We have carefully reviewed our data and methods to address these issues. Firstly, we have thoroughly checked our study population to ensure there is no overlap or duplication of data. We have verified that each participant included in the meta-analysis is unique and belongs to a distinct study. Additionally, we have revised the manuscript to provide more comprehensive details on the incidence data analysis. We have included additional information on the methods used, variables considered, and any limitations associated with the analysis. We believe these revisions address your concerns and enhance the robustness of our findings.

Q3: Missing Data File in Supplementary Files:

We apologize for the oversight regarding the missing corresponding data file in the supplementary files. And we added necessary supporting files at end of manuscript We appreciate your observation and have taken immediate action to rectify this issue. We have now included the complete and relevant data file in the supplementary materials as stated in the manuscript. The data file contains all the necessary information for transparency and reproducibility of our study. We thank you for bringing this to our attention, and we assure you that the data file is now available for reviewers and readers.

Reviewer #2:

#1 Abstract Since you have a relatively well articulated burden of the problem in the introduction better to reflect some summary figures in the background of the abstract for better conveyance of your studies relevance

#Responses; Thank you for your feedback on our manuscript. We have revised the abstract to include visually represented summary figures that highlight key statistics and trends related to active tuberculosis occurrence among children living with HIV post antiretroviral therapy in Ethiopia. These additions aim to enhance clarity and the overall impact of our study. We appreciate your input and welcome any further suggestions or comments you may have. Regarding the introduction, we have incorporated a discussion on global and national policies and initiatives to emphasize the significance of our study and provide a comprehensive overview. We apologize for any confusion caused by the error or typo in our data synthesis and analysis procedure. We will correct this mistake to ensure the accuracy of our findings and analysis. The revisions made to the introduction and data analysis sections will improve the overall quality of our study.

#2 Discussion; I think you should carefully review your discussion to include strong discussion points about reason you impute for your results, possible implications of the result especially on preventive therapies for seropositive children and future interventions you recommend the criticality of addressing anemia in this group of patients. Moreover, you might also go in depth on similar feature of the studies which might affect the results ...for ex you mentioned diagnostic methods of the studies ....you could further elaborate on that and compare it to global and national recommendations and the possible implication your result might have on diagnostic methods (using more sensitive diagnostic methods for identified at risk groups and so on)

#Responses #2; Thank you for your feedback on the discussion section of our manuscript. We had reviewed the discussed carefully and address based on your comment please see it gain suggestions for improvement. We had provided strong points explaining the reasons behind our results and emphasize the implications for preventive therapies and the importance of addressing anemia in seropositive children. We appreciate your valuable input and thorough review, and these revisions. Thank you for your time and consideration.

#3 Strength and Limitation: Please Given the challenges of Tb diagnosis specially in resource limited set ups like ours i expect you to delve more in to the scenarios surrounding the studies you pooled which might hamper the validity of your results. one such factor would be mostly

clinical stratification of seropositive patients or non-lab-supported staging, mostly, and sub-standard diagnostic capacities of our health facility.

#Responses for #3; The strengths of this study include an extensive search strategy, clear inclusion criteria, and the involvement of three independent authors in the quality assessment. However, there are several methodological limitations to consider. Firstly, there is a scarcity of well-described TB reports among studies in HIV-positive children compared to similar studies in adults. Furthermore, limitations such as reliance on clinical stratification or non-laboratory-supported staging, sub-standard diagnostic capacities in health facilities, a small number of included studies, and the use of retrospective data may potentially impact the validity of the results.

Reviewer #3

#3: Congratulations to the authors for their impactful work in the field of HIV and Tuberculosis, two serious debilitating infections that significantly impact each other as co-infections. The study demonstrates several strengths, including a well-chosen topic for meta-analysis and systematic review, with a large sample size that enhances representativeness. The study also follows a meticulous plan of action, as documented in the study. The strong conclusion emphasizes the need for further research and action, including changes in plans and education for healthcare professionals involved in the care of children living with HIV. Give clarification for Limitations and Suggestions a) Use standard terminologies across the entire study and please abbreviate with explanations the first time.

#Responses for reviewer #3; thanks for your response: we have addressed the limitations of this study. Firstly, we have ensured the use of standardized terminologies consistently throughout the entire report to enhance understanding and comparability. Secondly, we have provided abbreviations with explanations the first time they are used, improving readability and preventing confusion. These improvements have led to enhanced clarity and better understanding for readers.

b) The methodology is repeated about 4 times in the paper and hence becomes repetitive and cumbersome and hence would request you to kindly review and see how it may be framed better.

#Responses;

Thank you for your comment and we have addressed the concerns raised and removed the repetition of the methodology section.

c)Multiple spelling and grammatical errors to be addressed.

#Responses; we had addressed the extensively edited all grammatical errors for this document

d) References have not been done as per the required formatting and hence the data used for study purpose has not been reviewed by me.

#Responses; Thanks, we had addressed the listed reference based on PLOS public health journals

e) Multiple factual errors and statements with no scientific backing have been mentioned, there appears to be gross errors indicative of possible cut and copy approach from a previously done study.

#Responses; thanks, we have extensively edited and revised the suspected documents please accepted the let me invited you to read it again

f) References and data regarding prevalence of HIV, TB etc are all outdated and hence need to be updated accordingly. I am confident of your expertise to be able to re work this paper and make it fit to be published for maximal impact.

#Responses; Thanks for your feed back and comments we have used more updated reference and new published articles. We strongly recommended health care providers to use the mobile phones for supporting health and HIV treatment adherence were acceptable to patients with TB, mHealth interventions should consider language, mode of communication, and preferred timing for communication to improve uptake.
