# Supplementary material for: Proportion of active tuberculosis among HIV-infected children after antiretroviral therapy in Ethiopia: A systematic review and meta-analysis
Source: PLOS Glob Public Health. 2024 Aug 2;4(8):e0003528. doi: 10.1371/journal.pgph.0003528 (PMC11296650; doi:10.1371/journal.pgph.0003528)
Supplement: Attachment — Submitted filename: Reviewer report PGPH-D-23-02361.pdf [file pgph.0003528.s007.pdf]

Review

Title: **ACTIVE TUBERCULOSIS PREVALENCE AFTER ANTI-RETROVIRAL THERAPY AMONG SEROPOSITIVE CHILDREN LIVING IN ETHIOPIA: A SYSTEMATIC REVIEW AND META-ANALYSIS**

Preamble:
The researchers strive to synthesize and describe available evidence on burden of TB among children with HIV across Ethiopia over a 10-year period through systematic review and meta-analysis. HIV and TB remain high prevalence chronic infectious diseases in sub-Saharan Africa. The pooling of evidence on this problem across Ethiopia over a decade, if done well, can provide valuable insight to guide future actions to better address the problem.

Specific comments:

1. Titles:

Both titles should use the term "HIV infected children", or "children living with HIV" rather than "seropositive". Seropositive is a non-specific term and may apply to many different antigens/microbes… This wording should be standardized throughout the manuscript.

*"Active tuberculosis prevalence after anti-retroviral therapy
Among seropositive.."*

Short Title should be re-thought to include the key words children, HIV and Ethiopia.

Abstract:

2. Methods are incomplete – expand to state clearly key aspects of "PICO" in line with international standards for systematic reviews: population of interest, exposures (risk factors of interest), comparison groups where relevant, and clearly defined outcome of interest.

3. Conclusion:
"A significant number of HIV-positive children suffered from twine (TB and HIV) infection in Ethiopia."
Objective was determine "pooled prevalence" – but concludes "significant number". The wording in concluding statements should align to that in the wording in study objectives.
For clarity give more specific numerical values that correspond to the objective.
The word "twine" is strange, and confusing to readers.
A re-worded concluding statement for example could be… "TB occurs in one-tenth of HIV infected children on ART in Ethiopia"…

"the risks of TB were significantly higher with studies including WHO clinical stage III &IV"

This conclusion suggests that studies that included WHO state III & IV data were compared to studies that did not report WHO stage III & IV. Please re-phrase for clarity.

Introduction:

Generally the scope of the introduction gives good background to the topic of research.

4. It is noted that some of the articles that were finally selected for the meta-analysis are quoted in this section. Perhaps the author should omit them from this section – so that all review of the 13 articles is reported in results and discussion.

Some specific clarifications are needed.

5. "The waning of the immune system increases the endogenous reactivation of dormant TB bacilli in the lung[5]"

This statement on pathophysiology does not align to paediatric TB where most cases are young children who are presenting with PRIMARY TB, arising from NEW TB INFECTION, and not reactivation of latent TB. Please re-phrase accordingly…

6. *"TB-related mortality in children accounts for 40% of global TB deaths".*

Epidemiologic data quoted appears inaccurate, the following should be rechecked, and correct global epidemiologic data provided from appropriate literature such as the most current global TB report 2023, or the "Roadmap to ending TB in children and adolescents" 2023 edition.

7. *"25% of in-hospital deaths, and 18% of inpatient hospitalizations [8, 10-12]."*

State more clearly which setting / country / study population these proportions refer to. In current sentence it suggests that these are global figures – but the references appear to be localized to Ethiopian hospitals.

8. *"Extra-pulmonary Tuberculosis (EPTB) is the most common kind of tuberculosis (TB) diagnosed and treated in seropositive children …"*

This finding differs from known literature – confirm the quality of evidence, and reliability for this report that EPTB is more common than pulmonary TB in Ethiopian HIV+ children. There is likely bias or confounding in the quoted study.

9. *"According to national profiles of TB and HIV patients, 11% of TB cases had HIV, while 9.1% of HIV patients had active TB [17, 24, 25]."*

This is very relevant background evidence to this review, but the age-group to which these data applies is unclear. Please specify if this refers to all ages, or children, and

what the age bracket is. If it includes all ages – it will be appropriate to add specific figures for children given that this review focuses on children.

10. *"Several small-scale studies in Ethiopia found TB incidence ranging from 7.2% in Amhara [26] to 23.6% in the south [27] regions."*

Very relevant evidence – is this incidence or prevalence? Correct units for incidence would be no. incident cases per 1,000 population, or similar rate.

10. For the global audience who are unfamiliar with the geography of Ethiopia it will be helpful if terms specify where and what Amhara is – a county? Province? City? In northern, eastern, western, central region of Ethiopia?
e.g. "Amhara, a county in Northern Ethiopia".
Please attempt to provide this general insight either in introduction, or in methods sections.

11. "*Despite concomitant administration of ART with IPT demoting> 80% of active TB[5], due to their age and immune-suppressing of HIV, children experienced active TB incidence [28]*".

"Demoting" – wrong grammar please correct for clarity.
IPT should be written in full.
The point on young age and HIV causing immune-suppression should be expanded to give clarity on why these are risk factors for incident TB in children. This could be mentioned earlier in the paragraph on pathophysiology and risk factors for progression to active primary TB.

12. *"Thus, this systematic review and meta-analysis report aimed to estimate the pooled prevalence of active tuberculosis in HIV-infected children at national levels after ART started from December 30/2012 to January 1st, 2023"*

At end of introduction the study aim is stated, then repeated in the next two sections. Merge these sections so that study aim/objectives are stated once as last statement of the introduction. Ensure full scope is captured e.g. … "to determine the prevalence and predictors of …."
The review questions should be omitted or incorporated in earlier sections of the introduction.

Methods

General comment on methods.

13. Generally the organisation and flow of the methods section is not optimal. There is much repetition, and some key aspects are omitted. There are numerous sub-headings to sub-headings as opposed to clear paragraphs outlining the standard sections of methods.

The authors should review again the standardised approach to methodology for systematic reviews and meta-analyses and re-organise this section appropriately.

The PLOS journal provides useful guidance for authors regarding this. PROSPERO platform for reporting research proposals for systematic reviews and meta-analyses is also a useful guide to the key content sections, and the flow of information on methods.

Please re-organise the methods so that information flows in the chronologic sequence with which the activities are carried out.

Specific suggestions for methods

14. Study design and setting:
Introductory paragraph to the methods mixes study design with search strategy, which is then repeated in subsequent sections. Consider sticking to describing the study design as one sentence (systematic review and meta-analysis)

15. It will be helpful to global readers if you could here briefly describe study setting – a couple of sentences describing Ethiopia's regions. As I read the results I found it difficult to understand if the reported studies represented most of Ethiopia or were clustered in a small proportion of Ethiopia's regions. (this could be covered either here, or in background section).

16. Definitions for PICO (population of interest, intervention/exposure, comparator group/s, outcome) are scattered throughout various sections of the methods.

Please create a couple of paragraphs (not bullet points) to bring all the information on PICO definitions early in the methods (after study design and setting). Please ensure clear definitions are given as follows:

Population of interest – what was accepted as case definition for HIV infection in the papers reviewed? If children < 18 months were included would you require mention of HIV PCR antigen confirmation of diagnosis in the paper?

Intervention / Exposure of interest – appears to be ART?

Comparison group – if appropriate. Were there any comparison populations of interest? Those study participants not yet on ART? Especially from early cohorts prior to universal ART initiation at diagnosis?

Outcome
– Primary outcome (primary objective): prevalence or TB? Incidence of TB? Proportions with TB?
- Secondary outcomes (second objective): Predictors of / risk factors for TB. State key factors that you had most interest in, and define where relevant.

To illustrate this point – there appeared only one brief statement in the methods defining outcome of interest:

"The outcome variable for this review was the TB occurrence in HIV-positive children."

POOR ASCERTAINMENT OF THE OUTCOME OF INTEREST IS A MAJOR OMMISSION OF METHODOLOGY AND MUST BE CLARIFIED TO ENSURE CREDIBILITY OF THE FINDINGS.

17. Eligibility of Studies

Please create this heading as an important sub-section of the methods.
Capture all information on criteria for deciding if a study was eligible or in-eligible under this heading. Much of this is on page 5 of "inclusion criteria" and "exclusion criteria" should be converted from numbered criteria into paragraph format.

If cohort study paper did not give disaggregated information on timing of TB did you still include it?
If the TB outcome for the paper mixed all TB cases regardless of whether it occurred before or at time of start of ART, and failed to give the data of post-ART incident TB separately was the paper excluded?

18. The sections on search strategy, screening, selection of studies, and appraisal of final selected studies are written up in various sections, but the sub-headings are confusing and appear over-lapping….

"Data synthesis and extraction

QUALITY ASSESSMENT AND APPRAISAL PROCEDURES

DATA SYNTHESIS AND ANALYSIS PROCEDURES"

Please re-organise and re-word the sub-headings so that each section covers a different step in the work, and do not over-lap.
Some suggestions for distinct sub-headings and sequence are as follows:

Search strategy
Study screening and selection procedures
Data abstraction, cleaning and quality assessment
Data analysis

19. *"We used a random- effect model to assess the overall epidemiological survival patters and proportions of COVD-19 recover post admission as inpatients in Ethiopia, as well as statistically significant variable with their 95% CI's[39]. Meta-prop package was implemented to estimate the pooled proportion of recovery of COVDI-19 post hospitalized of cases in Ethiopia, and the pooled hazard ration of the associated predictors for time to recovery for COVDID-19 were evaluated from each article."*

[39] Egger M, Davey Smith G, Schneider M, Minder C. Bias in meta-analysis detected by a simple, graphical test. BMJ 1997;315:629-34.

This section on COVID-19 is surprising, no relation to TB mentioned. Careless spelling errors for COVID-19 are noted.
The reference 39 does not seem to correspond to COVID-19.

Please explain and correct this section as appropriate.

RESULTS

General comments on results:

20. Generally the tables and figures give fairly comprehensive results relevant to describing the selected studies, and responding to the study objectives.
The current narrative synthesis is very selective of bits and pieces from the tables and figures, and it does not do justice to the valuable information displayed in the tables and figures.

The authors need to ***enhance the narrative synthesis*** of this systematic review and meta-analysis to provide a more comprehensive synthesis of the information in the tables and figures.

Specific comments on results

21. Study screening
Please state the numbers identified, and screened out, AT ALL STEPS - separate results of screening by title, from screening by abstract.
Revise the PRISMA flow diagram accordingly – with separate box for titles, then next screening box for abstracts.
The arrows showing numbers of articles excluded between one level and the next are incorrectly located, they should be moved and placed between the relevant boxes.

22. Two of the final 13 studies (refs 27 and 46) appear to have either the same study population, or much overlap between the study populations. If this is true, the same study subjects are double-counted / duplicated throughout the synthesized results which is not statistically sound.

- Please explain different author names.
- Please verify if this is duplication of same study population or not.
- If they are different they may be retained. If they are the same participants – one study should be removed from the meta-analysis.

[46] Firew Tiruneh tuberculosis-incidence-among hiv-infected children on-haart-and-theirclinical-profile-retrospective-cohort-study-south-. Journal of AIDS & Clinical Research Research, Article Volume 11:3,2020, DOI: 1037421/jar202011808, 2020.

[27] Deyas FTY. Effect of highly active antiretroviral treatment on TB incidence among HIV infected children and their clinical profile, retrospective cohort study, South West Ethiopia. Scientific Reports 10:21468, https://doiorg/101038/s41598-020-78466-0 2020.

23. Descriptive characteristics of the studies. Table 1.

These are well tabulated in table 1, but only partially described in the introductory paragraph.

- Please create sub-heading "Descriptive characteristics of the studies."
- Please expand the narrative write-up to give the readers a more comprehensive description of the studies to align to the excellent information in table 1.
- It is preferrable to report *study level* descriptive data in this section - study design, region, setting, study demography, minimum-maximum sample size, mean age by study.
- Avoid synthesizing the numbers at this point. E.g. sample size ranged from …(smallest study) to …. (largest study). Mean age ranged from …. (lowest mean age in study x) to …. (highest mean age).
- Please explain the term *"Follow-up"* that is used to describe the study design for some studies. Please confirm if there were any studies of cross-sectional design as table 1 shows none.
- Please explain what HT and HC refer to – and combined HT&HC. This should also be described clearly in the methods section that covers factors of interest as it is analysed later as sub-group analyses.

24. Describing study setting: For global audience that may be unfamiliar with Ethiopia – consider adding a supplemental figure map of Ethiopia that highlights where each study is located within Ethiopia. In narrative brief statement on where these identified regions are – e.g. mainly in central and southern regions of Ethiopia? The selected studies represent what proportion of the countries regions?

25. Table formatting comments:
- Please enhance column headings as follows:
- state the unit for age (years?)
- Events – specify what event (No. children with TB?)
- Prevalence (percent?)
- For all abbreviations in the table please explain them in the table footer.

Results for study objectives

26. Results on objective 1 is reported under a sub-heading "meta-analysis" which is confusing, since all the analyses reported in figures 2 to 6 are meta-analyses. Suggest you re-word the sub-headings using the actual language of the objective, as this makes results sections clear to reader.

Suggested sub-headings for the remaining results:

- Pooled Prevalence of TB in Children with HIV on ART.
- Pooled incidence of TB in Children with HIV on ART
- Factors associated with TB in Children with HIV on ART

Objective 1 results - Figure 2 & table 1.

27. The majority (7/13= 53.8%) [1, 4, 23, 27, 29, 43, 47] of studies reported TB magnitudes, whereas the remaining four [26, 44, 48, 49] included crude incidence.

The challenges of failure to clearly define the outcome of interest are reflected here.

PLEASE DECIDE WHAT YOUR OBJ. 1 OUTCOME MEASURE IS AND ADHERE TO THE SAME WORDING THROUGHOUT THE MANUSCRIPT.
Magnitude needs to be quantified using a specific statistical measure such as Prevalence or proportion? Crude incidence vs incidence?

Here 7 + 4 = 11 studies. Two studies omitted here, explain why they were omitted here.

"The final meta-analysis report indicated the pooled active TB prevalence among children after ART was estimated to be 12.1% (95% CI: 10.7 - 13.4; I2 = 63.4%, p=0.001) ***(Fig 1)."***

Wrong figure listed – I assume this statement reports on Figure 2. Here the researchers do not differentiate cohort and cross-sectional studies – please explain your approach to merge cohort and cross-sectional study to arrive at pooled prevalence for all.

28. I propose that you consider reporting separately the prevalence results for the 7 cross-sectional studies in a distinct forest plot. E.g. Prevalence of TB reported in **seven** cross-sectional studies ranged from …. (lowest prevalence) in study x, to …..(highest prevalence) in study z. Then the meta-analyses pooled prevalence for the 7 cross-sectional studies.

Then report on the 9 cohort study findings – if they provided both proportions of children with TB (surrogate for prevalence?), as well as incidence of TB – these may be reported in separate forest plots.

Cohort study forest plot (9 studies) with proportions (prevalence) of children with TB, and pooled prevalence for the 9 studies
Then a supplemental forest plot can combine the 13 studies.

29. There is need to report separately the **results for TB incidence** across those **nine** cohort studies that reported on it. It would be useful to report the pooled incidence and provide a separate forest plot for this value.

Objective 2 results: Factors associated with TB. Figures 3 - 6

Generally very nice sub-group analyses comparing various sub-groups at geographical level, study setting, and patient-specific risk factors.

30. Two headings "sub-group analyses" and "risk factors analyses" not necessary. Both sections cover objective 2, and both involve sub-group analyses – so the different headings are confusing. Please merge the two sections and use one heading e.g. **"Factors associated with TB in children with HIV on ART"**

31. There is inadequate detail in explaining clearly some of the sub-group variables, and incomplete narrative for each forest plot. The researchers are encouraged to ensure the following:

- That each sub-group is clearly defined where appropriate (e.g. HT vs HT&HC, but plot reports facility and hospital, IPT no definition, is there a non-IPT population, and how are they defined)

- That the risk group and reference group to which it is compared is clearly stated (e.g. IPT and cotrimoxazole meta-analyses, it is unclear who is compared to whom)

- As a rule – indicate the figure immediately after the first sentence that reports data results from the specific figure. This guidance is given in PLOS author guidelines with examples.

32. *"In our subgroup analysis, TB prevalence among HIV-positive children in studies in Hospital setup was slightly lower than facility-based studies at 11.05% (95%CI: 9.4- 12.3 Vs. 14.1%; 95%CI: 11.74- 16.33)."*

A new term appears "facility-based" that was not described in table 1.
For studies in HT versus studies in HT&HC settings was data of those children reported in HC only separated to provide this result on "facility-based" sub-group? Please clarify. If so – a supplemental data table that stratifies data (numerator – events, and denominator number of children) for the studies that enrolled children from both HT and HC settings would be helpful, and would give confidence of validity of this finding.

33. There is no forest plot for the comparison of regions, but there is narrative reporting on the same. Please provide the forest plot.

34. Comments on formatting and legends of the figures:
- If possible add a column of the actual numbers for sample size of each study within the plot against each study, and the pooled sample size.
- In the legend, after the legend title add more information as appropriate. E.g. fig 5 and fig 6. State selection criteria for this sub-set of children that are analysed in the plots.
- Figure 4: State the unit of follow-up period – I assume it is years? Insert it within the forest plot, and also state so in the figure legend.
- The <10 and >10 excludes those with exactly 10 years follow-up. Please revise so that it is clear which group includes those with 10 years. (use ≤ sign)

35. Discussion
Once the suggested improvements are done in the analysis and presentation of results – especially separation of prevalence from incidence results, the discussion should be revised accordingly.

36. Conclusions
May be refined based on comments given for study abstract concluding statement, and enhanced to incorporate conclusions on pooled incidence as well as pooled prevalence.

Final Remarks

Important research area – the evidence is valuable to guide solutions for this problem. Generally the research provides valuable findings, however there is need to enhance and refine aspects of the methods and results to optimise the reporting of this important review and meta-analyses.

The most concerning issues identified in the manuscript are lack of clarity on the statistical measure for the primary outcome "magnitude or burden of TB". Varied outcomes are mentioned – pooled prevalence, incidence data is mentioned. There is a predominance of cohort studies reviewed, but no definitions of how authors determined / defined prevalence, and how they abstracted the prevalence data from cohort study papers. There are results on incidence, but meta-analyses not provided. – which is what one would have expected from cohort studies.

Another important issue is possible duplication of the same study population in two of the papers (refs 27 and 46), which if confirmed means that all meta-analyses must be re-done removing one of the papers.

Major revisions are required.
If the major issues are adequately addressed, this paper may be considered for publication.
