# Supplementary material for: Proportion of active tuberculosis among HIV-infected children after antiretroviral therapy in Ethiopia: A systematic review and meta-analysis
Source: PLOS Glob Public Health. 2024 Aug 2;4(8):e0003528. doi: 10.1371/journal.pgph.0003528 (PMC11296650; doi:10.1371/journal.pgph.0003528)
Supplement: Attachment — Submitted filename: Response to Reviewers.docx [file pgph.0003528.s005.docx]

**Authors response for reviewers’ comments**

Version =2

Number of authors= 5

Revised tittles:

**Meta-Analysis of Active Tuberculosis Occurrence among Children Living with HIV Post Anti-Retroviral Therapy Initiated in Ethiopia**

**Editor’s comment**

#1. Please provide a complete some minor occurrence of overlapping text with the following previous publication(s), which needs to be addressed: <https://doi.org/10.1155/2022/9925693>

\# Response: Thanks for your comments and we clarified all text overlap with previous manuscript and clear, edited, and the introduction of this manuscript cleared it read it again

#2 Please provide separate figure files in .tif or .eps format only and remove any figures embedded in your manuscript file. Please also ensure all files are under our size limit of 10MB. Based on PLOS Global public Health Journals manuscript Figure presentation Guideline

\# Responses: Thanks for your comment we have amended and wrong reference styles and make as if the PLOS Global journals submission guide, we had submitted as if it. please read it :

Reviewer #1:

1. Reviewer #1: All comments given in attachment. Q1 and Q2 - main concerns - there may be one duplicate study population - researchers need to verify that this is not the case. Incidence data analysis incomplete. The rest of the analyses is done fairly comprehensively and appropriately. Q3. I could not find the corresponding data file in supplementary files - although the pdf manuscript states that the data was provided.

\# **Responses; Thanks for your comments** and feedback on our manuscript titled "Meta-Analysis of Active Tuberculosis Occurrence among Children Living with HIV Post Anti-Retroviral Therapy Initiated in Ethiopia." We have addressed your concerns regarding duplicate study population and incomplete incidence data analysis by carefully reviewing our data and methods. We have verified the uniqueness of each participant included in the meta-analysis and provided additional comprehensive details on the incidence data analysis in the revised manuscript. We apologize for the missing data file in the supplementary files and have rectified this issue by including the complete and relevant data file for transparency and reproducibility. Thank you for bringing this to our attention, and we assure you that the data file is now available for review.

For your question of Please find our responses to your comments for

Q1 and Q2 question how could you managed duplication and missing data

We acknowledge your concerns regarding the possibility of a duplicate study population and the incomplete analysis of incidence data. We have carefully reviewed our data and methods to address these issues. Firstly, we have thoroughly checked our study population to ensure there is no overlap or duplication of data. We have verified that each participant included in the meta-analysis is unique and belongs to a distinct study. Additionally, we have revised the manuscript to provide more comprehensive details on the incidence data analysis. We have included additional information on the methods used, variables considered, and any limitations associated with the analysis. We believe these revisions address your concerns and enhance the robustness of our findings.

**Q3: Missing Data File in Supplementary Files:**

We apologize for the oversight regarding the missing corresponding data file in the supplementary files. And we added necessary supporting files at end of manuscript We appreciate your observation and have taken immediate action to rectify this issue. We have now included the complete and relevant data file in the supplementary materials as stated in the manuscript. The data file contains all the necessary information for transparency and reproducibility of our study. We thank you for bringing this to our attention, and we assure you that the data file is now available for reviewers and readers.

**Reviewer #2:**

#1 Abstract Since you have a relatively well articulated burden of the problem in the introduction better to reflect some summary figures in the background of the abstract for better conveyance of your studies relevance

#Responses; Thank you for your feedback on our manuscript. We have revised the abstract to include visually represented summary figures that highlight key statistics and trends related to active tuberculosis occurrence among children living with HIV post antiretroviral therapy in Ethiopia. These additions aim to enhance clarity and the overall impact of our study. We appreciate your input and welcome any further suggestions or comments you may have. Regarding the introduction, we have incorporated a discussion on global and national policies and initiatives to emphasize the significance of our study and provide a comprehensive overview. We apologize for any confusion caused by the error or typo in our data synthesis and analysis procedure. We will correct this mistake to ensure the accuracy of our findings and analysis. The revisions made to the introduction and data analysis sections will improve the overall quality of our study.

**#2 Discussion**; I think you should carefully review your discussion to include strong discussion points about reason you impute for your results, possible implications of the result especially on preventive therapies for seropositive children and future interventions you recommend the criticality of addressing anemia in this group of patients. Moreover, you might also go in depth on similar feature of the studies which might affect the results ...for ex you mentioned diagnostic methods of the studies ....you could further elaborate on that and compare it to global and national recommendations and the possible implication your result might have on diagnostic methods ( using more sensitive diagnostic methods for identified at risk groups and so on)

#Responses #2; Thank you for your feedback on the discussion section of our manuscript. We had reviewed the discussed carefully and address based on your comment please see it gain suggestions for improvement. We had provided strong points explaining the reasons behind our results and emphasize the implications for preventive therapies and the importance of addressing anemia in seropositive children. We appreciate your valuable input and thorough review, and these revisions. Thank you for your time and consideration.

#3 Strength and Limitation: Please Given the challenges of Tb diagnosis specially in resource limited set ups like ours i expect you to delve more in to the scenarios surrounding the studies you pooled which might hamper the validity of your results. one such factor would be mostly clinical stratification of seropositive patients or non-lab-supported staging, mostly, and sub-standard diagnostic capacities of our health facility.

#Responses for #3; The strengths of this study include an extensive search strategy, clear inclusion criteria, and the involvement of three independent authors in the quality assessment. However, there are several methodological limitations to consider. Firstly, there is a scarcity of well-described TB reports among studies in HIV-positive children compared to similar studies in adults. Furthermore, limitations such as reliance on clinical stratification or non-laboratory-supported staging, sub-standard diagnostic capacities in health facilities, a small number of included studies, and the use of retrospective data may potentially impact the validity of the results.

**Reviewer #3**

#3: Congratulations to the authors for their impactful work in the field of HIV and Tuberculosis, two serious debilitating infections that significantly impact each other as co-infections. The study demonstrates several strengths, including a well-chosen topic for meta-analysis and systematic review, with a large sample size that enhances representativeness. The study also follows a meticulous plan of action, as documented in the study. The strong conclusion emphasizes the need for further research and action, including changes in plans and education for healthcare professionals involved in the care of children living with HIV. Give clarification for Limitations and Suggestions a) Use standard terminologies across the entire study and please abbreviate with explanations the first time.

#Responses for reviewer #3; thanks for your response: we have addressed the limitations of this study. Firstly, we have ensured the use of standardized terminologies consistently throughout the entire report to enhance understanding and comparability. Secondly, we have provided abbreviations with explanations the first time they are used, improving readability and preventing confusion. These improvements have led to enhanced clarity and better understanding for readers.

b) The methodology is repeated about 4 times in the paper and hence becomes repetitive and cumbersome and hence would request you to kindly review and see how it may be framed better.

#Responses;

Thank you for your comment and we have addressed the concerns raised and removed the repetition of the methodology section.

c)Multiple spelling and grammatical errors to be addressed.

#Responses; we had addressed the extensively edited all grammatical errors for this document
d) References have not been done as per the required formatting and hence the data used for study purpose has not been reviewed by me.

#Responses; Thanks, we had addressed the listed reference based on PLOS public health journals
e) Multiple factual errors and statements with no scientific backing have been mentioned, there appears to be gross errors indicative of possible cut and copy approach from a previously done study.

#Responses; thanks, we have extensively edited and revised the suspected documents please accepted the let me invited you to read it again

f) References and data regarding prevalence of HIV, TB etc are all outdated and hence need to be updated accordingly. I am confident of your expertise to be able to re work this paper and make it fit to be published for maximal impact.

#Responses; Thanks for your feed back and comments we have used more updated reference and new published articles. We strongly recommended health care providers to use the mobile phones for supporting health and HIV treatment adherence were acceptable to patients with TB, mHealth interventions should consider language, mode of communication, and preferred timing for communication to improve uptake.
