# Supplementary material for: Proportion of active tuberculosis among HIV-infected children after antiretroviral therapy in Ethiopia: A systematic review and meta-analysis
Source: PLOS Glob Public Health. 2024 Aug 2;4(8):e0003528. doi: 10.1371/journal.pgph.0003528 (PMC11296650; doi:10.1371/journal.pgph.0003528)
Supplement: Attachment — Submitted filename: Point by point response.docx [file pgph.0003528.s008.docx]

**Point by point response for editors and reviewers**

**Journal name: PloS One global public health**

**Title: Active Tuberculosis Prevalence in HIV-Infected Children on Antiretroviral Therapy in Ethiopia: A Systematic Review**

**Corresponding author: Fassikaw Kebede**

**Email:** [fassikaw123@gmail.com](mailto:fassikaw123@gmail.com)

Dear Editor	and Reviewer,

Thank you very much for your helpful feedback, suggestions, and questions regarding each subsection. We have carefully addressed each point based on your comments and have made the necessary amendments for the final version of this manuscript.

To ensure the highest quality of the manuscript, we followed a three-step process. First, each individual author extensively edited the manuscript. Next, we utilized online editing tools to further refinee the content of the manuscript to be fascination and for final publication steps. Finally, we sought the guidance of senior researchers, including Dr. Melaku Tegegne (From Wollongong University), who have extensive experience in writing, editing, and re-writing scientific manuscripts. We have incorporated the reviewer's comments into the manuscript by providing a point-by-point response below. Additionally, we have edited the manuscript to align with the journal's standards, as suggested by the Editor and reviewer. Thank you once again for your valuable input particular Reviewer one, which has significantly improved the quality of our manuscript.

**Reviewer 1**

**Comment 1. Titles:**

Both titles should use the term “HIV infected children”, or “children living with HIV” rather than “seropositive”. Seropositive is a non-specific term and may apply to many different antigens/microbes… This wording should be standardized throughout the manuscript. “Active tuberculosis prevalence after anti-retroviral therapy Among seropositive.” Short Title should be re-thought to include the key words children, HIV and Ethiopia.

**Response: We modified the tittle as ‘’Prevalence of Active Tuberculosis in Children Living with HIV on Anti-Retroviral therapy in Ethiopia - A Meta-Analysis’’ and short title as ‘’Meta-analysis of Active Tuberculosis in CLHIV on ART in Ethiopia’’.**

Abstract:

**Comment 2.** Methods are incomplete – expand to state clearly key aspects of “PICO” in line with international standards for systematic reviews: population of interest, exposures (risk factors of interest), comparison groups where relevant, and clearly defined outcome of interest.

**Response:** thanks for your commences and suggestions to be aligned with your suggestion of this manuscript we have edited and re-amended the full manuscript once again using PICO frames follows**.** This systematic review and meta-analysis employed the PICO (Population, Intervention, Comparison, and Outcomes) framework to assess the eligibility of the articles and enhance evidence-based medicine and research by facilitating the structuring of clinical or research questions. This included as follows (P) Population of interest: Children living with HIV on anti-retroviral therapy in Ethiopia,(I) Intervention; all children HIV infected children started Anti-retroviral therapy, (C) Comparison; children without active TB with stand on HIV cohort (O )Outcome of interest: active TB in HIV-infected children found in Ethiopia were used for PICO frameworks. Please you can look at page 5 line 145 to 154

**Comment 3.** Conclusion: “A significant number of HIV-positive children suffered from twine (TB and HIV) infection in Ethiopia.” Objective was determine “pooled prevalence” – but concludes “significant number”. The wording in concluding statements should align to that in the wording in study objectives. For clarity give more specific numerical values that correspond to the objective. The word “twine” is strange, and confusing to readers. A re-worded concluding statement for example could be… “TB occurs in one-tenth of HIV infected children on ART in Ethiopia”…“the risks of TB were significantly higher with studies including WHO clinical stage III &IV” Please re-phrase for clarity.

**Response: We made appropriate change on the manuscript particularly the introduction part has been shuffle in some parts Thank you so much for your constructive comments.**

**Comment 4.** It is noted that some of the articles that were finally selected for the meta-analysis

are quoted in this section. Perhaps the author should omit them from this section so that all review of the 13 articles is reported in results and discussion. Some specific clarifications are needed.

**Response: thanks for your reviewing and indispensible comments since the one which we used for only comparison at regional level prevalence of active TB and made appropriate change on the manuscript particularly idea related with included met-analysis articles were removed and substituted by others which have similar contextual. you can look for from page 3 to page 5 with line number73 to 140**

**Comment 5.** “The waning of the immune system increases the endogenous reactivation of dormant TB bacilli in the lung [5]”. This statement on pathophysiology does not align to pediatric TB where most cases are young children who are presenting with PRIMARY TB, arising from NEW TB INFECTION, and not reactivation of latent TB. Please re-phrase accordingly.

**Response: We rephrased the sentence. Thank you so much for your suggestion.**

**Comment 6.** “TB-related mortality in children accounts for 40% of global TB deaths”. Epidemiologic data quoted appears inaccurate, the following should be rechecked, and correct global epidemiologic data provided from appropriate literature such as the most current global TB report 2023, or the “Roadmap to ending TB in children and adolescents” 2023 edition.

**Response: thanks for your comments and suggestion for the point you raised, and we have removed the point that never ever fitted idea generated and substituted by similar contextual words.**

**Comment 7.** “25% of in-hospital deaths, and 18% of inpatient hospitalizations [8, 10-12].” State more clearly which setting / country / study population these proportions refer to. In current sentence it suggests that these are global figures – but the references appear to be localized to Ethiopian hospitals.

**Response: The figures are used to show mortality in Ethiopia. The references used there are not global (for instance 8 & 9). Thank you for your comment, nevertheless we have removed and substituted and amended with other similar contextual reference figure. You can look on page 4 line number 96to 104**

**Comment 8.** “Extra-pulmonary Tuberculosis (EPTB) is the most common kind of tuberculosis

(TB) diagnosed and treated in seropositive children …” This finding differs from known literature – confirm the quality of evidence, and reliability for this report that EPTB is more common than pulmonary TB in Ethiopian HIV+ children. There is likely bias or confounding in the quoted study.

**Response:** We made changes to the sentence. According to studies conducted outside of Ethiopia, extra-pulmonary tuberculosis is more common in children than pulmonary tuberculosis. This is due to the fact that pulmonary tuberculosis in children is not well identified because they do not cough, and health practitioners frequently misdiagnose them. However, this was not confirmed/studied in Ethiopia, therefore we removed the sentence.

**Comment 9.** “According to national profiles of TB and HIV patients, 11% of TB cases had HIV, while 9.1% of HIV patients had active TB [17, 24, 25].” This is very relevant background evidence to this review, but the age-group to which these data applies is unclear. Please specify if this refers to all ages, or children, and what the age bracket is. If it includes all ages – it will be appropriate to add specific figures for children given that this review focuses on children.

**Response: we have substituted and amended based on your suggested idea . Thank you for your comment. We have accepted your suggestion**

**Comment 10**. “Several small-scale studies in Ethiopia found TB incidence ranging from 7.2% in

Amhara [26] to 23.6% in the south [27] regions.” Very relevant evidence – is this incidence or prevalence? Correct units for incidence would be no. incident cases per 1,000 population, or similar rate.

**Response:** **It is prevalence and we made appropriate change on the sentence.**

For the global audience who are unfamiliar with the geography of Ethiopia it will be helpful if terms specify where and what Amhara is – a county? Province? City? In northern, eastern, western, central region of Ethiopia? e.g. “Amhara, a county in Northern Ethiopia”. Please attempt to provide this general insight either in introduction, or in methods sections.

**Response:** **We made appropriate corrections based on your suggestion. Thank you for your comment and suggestion.**

**Comment 11**. “Despite concomitant administration of ART with IPT demoting> 80% of active TB [5], due to their age and immune-suppressing of HIV, children experienced active TB incidence[28]”. “Demoting” – wrong grammar please correct for clarity. IPT should be written in full. The point on young age and HIV causing immune-suppression should be expanded to give clarity on why these are risk factors for incident TB in children.

**Response;** Thank you for your comment and amended the give point you raised as follows Concomitant administration of ART with isoniazid preventive Therapy (IPT) had significantly effect of reducing active TB cases by over 80% HIV infected Children [21]. However, IPT completion rate and adherence of ART has affected by caregivers and regimen characteristic[18]. Although several small-scale studies have reported on the epidemiology of TB/HIV co-infection among HIV-infected children in various parts of Ethiopia [5, 12, 13]

You can look on page 4 line 108 to 113

**Comment 12**. “Thus, this systematic review and meta-analysis report aimed to estimate the

pooled prevalence of active tuberculosis in HIV-infected children at national levels after ART started from December 30/2012 to January 1st, 2023” At end of introduction the study aim is stated, then repeated in the next two sections. Merge these sections so that study aim/objectives are stated once as last statement of the introduction. Ensure full scope is captured e.g. … “to determine the prevalence and predictors of ….” The review questions should be omitted or incorporated in earlier sections of the introduction.

**Response: We made change. Thank you so much for your comment. We have amended as follow** “this systematic review and meta-analysis aimed to estimate the pooled burden of active TB among HIV-infected children” this is final objective of this manuscript after merged the two idea you mentioned and sentence.

**Comment 13**. Generally, the organization and flow of the methods section is not optimal. There is much repetition, and some key aspects are omitted. There are numerous sub headings to sub-headings as opposed to clear paragraphs outlining the standard sections of methods. The authors should review again the standardized approach to methodology for systematic reviews and meta-analyses and re-organize this section appropriately. Please re-organize the methods so that information flows in the chronologic sequence with which the activities are carried out. Specific suggestions for methods

Response: hanks editors and reviewer if you are interested astonishingly we have amended extensile on the manuscript particularly Method section please referee from page 4 to 7 as follow ; This systematic review was used five international electronic databases were mainly used including Scopus, PubMed, Medline, Web of Science, and Google scholar. The searching was focused on English language published articles and the searching was done back from January 1, 2023 to December 30, 2012 back to 10 years to provide up-to-date pooled estimates of active TB burdenin HIV infected Children. We employed controlled vocabulary terms (MeSH) and free text to extract relevant articles. The search included topics such as active tuberculosis, pulmonary TB, extra pulmonary TB, HIV infection, individuals, children, pediatrics, neonates, lymphadenitis, disseminated TB, and Ethiopia. The search terms used to identify relevant studies included "Epidemiology" OR "Incidence" OR "Case fatality" "Tuberculosis" OR "Pulmonary Tuberculosis" OR "Disseminated Tuberculosis" OR "Lymphadenitis" AND "HIV" OR "AIDS" AND "Children" OR "Pediatrics" OR "Infant" AND "Ethiopia". This systematic review and meta-analysis employed the PICO (Population, Intervention, Comparison, and Outcomes) framework to assess the eligibility of the articles and enhance evidence-based medicine and research by facilitating the structuring of clinical or research questions. This included as follows (P) Population of interest: Children living with HIV on anti-retroviral therapy in Ethiopia,(I) Intervention; all children HIV infected children started Anti-retroviral therapy, (C) Comparison; children without active TB with stand on HIV cohort (O )Outcome of interest: active TB in HIV-infected children found in Ethiopia were used for PICO frameworks. Inclusion and exclusion Criteria:-This systematic review and meta-analysis report had the following inclusion criteria. 1) scientific papers reporting co-infections of TB and HIV in HIV-infected children in Ethiopia, 2) articles containing burden or incidence reports of active TB in HIV-infected children, 3) studies published within the past ten years (up to December 30, 2023), with cross-sectional or cohort designs and published in English, and 4) study subjects limited to children aged ≤15 years. Whereas this systematic review had exclusion to removed unfitted articles before analysis, Articles without a journal name and/or author, conference proceedings and qualitative studies were removed.

**Comment 14**. **Study design, setting**, **and PROSPERO platform** for registering and reporting research proposals for systematic reviews and meta-analyses is also a useful guide to the key content sections, and the flow of information on

**Response**: thanks for your comments We stated the included articles what should be the design and setting before the analysis to be included in the inclusion criteria please make look for on page 5 line number 130 to 140 ; **T**his systematic review and meta-analysis report had the following inclusion criteria. 1) scientific papers reporting co-infections of TB and HIV in HIV-infected children in Ethiopia, 2) articles containing burden or incidence reports of active TB in HIV-infected children, 3) studies published within the past ten years (up to December 30, 2023), with cross-sectional or cohort designs and published in English, and 4) study subjects limited to children aged ≤15 years. Whereas this systematic review had exclusion to removed unfitted articles before analysis, Articles without a journal name and/or author, conference proceedings and qualitative studies were removed and citations without abstract and/or full-text were also excluded before analysis.

**Comments 14.1: did this manuscript registered in PROSPERO platform** for and used the flow of information using PRISMA guideline

**Response :** thanks for reminding to be registered this manuscript and here what we did “Preferred Reporting Items for Systematic Reviews and Meta-Analysis (PRISMA) guideline was followed to report the findings of the selected articles **(S1=PRISMA 2020 checklist )**[22]. Additionally, this systematic review and meta-analysis have been registered in the Prospero protocol with the identification number CRD42024502038”.

**Comment 15**. It will be helpful to global readers if you could here briefly describe study setting a couple of sentences describing Ethiopia’s regions. As I read the results I found it difficult to understand if the reported studies represented most of Ethiopia or were clustered in a small proportion of Ethiopia’s regions. (this could be covered either here, or in background section).

**Response: thanks for your suggestion, there are three types of study setting that articles were published including data were collected from health center ,Hospital set up and both health center and Hospital. In Ethiopia there are nine regions, and two administrative , which are Oromia , Tigray, Amhara, Bensihsnagul, Somalia, Southern nation nationalities, Afar, Harir, and two city administrative Dire Daw and Addis-Ababa**

**Comment 16**. Definitions for PICO (population of interest, intervention/exposure, comparator

group/s, outcome) are scattered throughout various sections of the methods. Please create a couple of paragraphs (not bullet points) to bring all the information on PICO definitions early in the methods (after study design and setting). Please ensure clear definitions are given as follows: Population of interest – what was accepted as case definition for HIV infection in the papers reviewed? If children < 18 months were included would you require mention of HIV PCR antigen confirmation of diagnosis in the paper? Intervention / Exposure of interest – appears to be ART? Comparison group – if appropriate. Were there any comparison populations of interest? Those study participants not yet on ART? Especially from early cohorts prior to universal ART initiation at diagnosis? Outcome – Primary outcome (primary objective): prevalence or TB? Incidence of TB? Proportions with TB?

**Response: Thank you so much for your constructive comment. We have added all components you mentioned in the methods section and we reorganized the methods section. For instant here are listed** this systematic review and meta-analysis employed the PICO (Population, Intervention, Comparison, and Outcomes) framework to assess the eligibility of the articles and enhance evidence-based medicine and research by facilitating the structuring of clinical or research questions. This included as follows (P) Population of interest: Children living with HIV on anti-retroviral therapy in Ethiopia,(I) Intervention; all children HIV infected children started Anti-retroviral therapy, (C) Comparison; children without active TB with stand on HIV cohort (O )Outcome of interest: active TB in HIV-infected children found in Ethiopia were used for PICO frameworks.

**Comment 17**. Eligibility of Studies Please create this heading as an important sub-section of the methods. Capture all information on criteria for deciding if a study was eligible or in-eligible under this heading. Much of this is on page 5 of “inclusion criteria” and “exclusion criteria” should be converted from numbered criteria into paragraph format. If cohort study paper did not give disaggregated information on timing of TB did you still include it? If the TB outcome for the paper mixed all TB cases regardless of whether it occurred before or at time of start of ART, and failed to give the data of post-ART incident TB separately was the paper excluded?

**Response:** We made gross change on the eligibility studies. Thank you for your comment. Here are the final inclusion and exclusion criteria this systematic review and meta-analysis report had included a given articles with defined outcome of any TB types in HIV infected children with the following inclusion criteria. 1) scientific papers reporting co-infections of TB and HIV in HIV-infected children in Ethiopia, 2) articles containing burden or incidence reports of active TB in HIV-infected children, 3) studies published within the past ten years (up to December 30, 2023), with cross-sectional or cohort designs and published in English, and 4) study subjects limited to children aged ≤15 years. Whereas this systematic review had exclusion to removed unfitted articles before analysis, Articles without a journal name and/or author, conference proceedings and qualitative studies were removed. Furthermore, citations without abstract and/or full-text were also excluded before analysis.

**Comment 18**. The sections on search strategy, screening, selection of studies, and appraisal of

final selected studies are written up in various sections, but the sub-headings are confusing and appear over-lapping….“Data synthesis and extraction QUALITY ASSESSMENT AND APPRAISAL PROCEDURES DATA SYNTHESIS AND ANALYSIS PROCEDURES” Please re-organize and re-word the sub-headings so that each section covers a different step in the work, and do not over-lap and some suggestions for distinct sub-headings and sequence are as follows: thanks for your comments we have re-organized

**Response:** We made change based on your suggestion points as follow please make sure and check what we did based on your suggested point for final comments.

1. **Study screening and selection procedures**; based on your comments we have used for Study screening and selection procedures as follows with subheading
   1.1. **Inclusion criteria for final analysis**; **this** systematic review and meta-analysis report had included a given articles with defined outcome of any TB types in HIV infected children with the following inclusion criteria. 1) scientific papers reporting co-infections of TB and HIV in HIV-infected children in Ethiopia, 2) articles containing burden or incidence reports of active TB in HIV-infected children, 3) studies published within the past ten years with **cross-sectional or cohort designs and published** in English, and 4) study subjects limited to children aged ≤15 years where as the

**1.2 Exclusion criteria included;** Studies that reported lacking abstracts and/or full-text, anonymous reports, editorials, and qualitative studies were excluded from the analysis. Furthermore, prior to the analysis, unfitted articles without a journal name and/or author, lacked the year of publication, and citations without abstracts and/or full-text were removed

**2. Data abstraction** Based on your comments we used such narration for article cleaning , quality assessment before finally selected articles **:** Four Authors (FK, BN, DT, and TK) independently extracted articles and evaluated the quality of each study by determining the eligibility based on given criteria for selection of studies. The discussion was used to settle any disagreement or uncertainty that arose during the article extraction and removing duplication process. These reviewers assessed the full-text articles; if one or more of them believed an article could be significant, it qualified after the article was carefully examined its titles, abstracts, and full text by three authors (FK, TK, and DT) used a Microsoft Excel spreadsheet to extract the specifics of each article. Three independent reviewers assessed each included article's quality using the JBI checklists given for all articles **[****24****,** **25****].** All eligible studies approved by all authors’ agreements about principal investigators, year of publication, study period, study setting, study population, and sample size retrieved from the identified articles. The biases of primary studies checked, assessed and screened by four authors (FK, DT, BN and TK), evaluated, and screened (**S2).** Any disagreements among reviewers regarding the critical appraisal were settled through discussion and building consensus for submission.

**3. Data analysis: we used** such narration for your subheading suggested comments Using End-Note Aversion 8.1, all detected and potentially suitable published article citations were exported and gathered; duplications were eliminated during the selection and screening processes. Two independent reviewers (FK, and TK) first reviewed the abstracts of the publications before moving on to the full-text articles, which they then evaluated following the particular standards for ultimate inclusion and modifying the data on a Microsoft Excel spreadsheet, and employed the STATA version 17 for further analysis. Descriptive statistics, and weighted inverse variance random-effects meta-regression were used to present the review's results to estimate pooled burden of active TB in HIV infected children [26]. The eligible articles were extracted using Meta-XL Excel version 5.3sheet [27] using identified risk factors from each selected studies and made combined each categorical variables and estimated risk factors for active TB [26]. The Higgs I^2^ statistics were utilized to detect heterogeneity among studies and elaborated using Cochran's Q test [28]. The degrees of statistical heterogeneity between the studies were assessed using I2 statistics; values of 25%, 50%, and 75% were thought to indicate modest, medium, and high levels of heterogeneity, respectively [38]. The source of heterogeneity among studies was examined using the subgroup and sensitivity analysis. The random effect regression model was used for the data-identified heterogeneous analysis [26]. The publication biases were assessed by visual inspection of funnel plots of the graph and quantitative using Egger’s weighted regression at p <0.1[29, 30]. In addition, we performed a leave-one-out sensitivity analysis to confirm a study with a biased direction of pooled estimates of Begs and Eggers tests[26]. The publication biases were assessed using inspection of funnel plots of the shape of the graph, quantitatively using Egger's weighted regression test at P-value <0.1[29, 30].

**Comment 19**. “We used a random- effect model to assess the overall epidemiological survival

patters and proportions of COVD-19 recover post admission as inpatients in Ethiopia, as well as statistically significant variable with their 95% CI’s[39]. Metaprop package was implemented to estimate the pooled proportion of recovery of COVDI-19 post hospitalized of cases in Ethiopia, and the pooled hazard ration of the associated predictors for time to recovery for COVDID-19 were evaluated from each article.” [39] Egger M, Davey Smith G, Schneider M, Minder This section on COVID-19 is surprising, no relation to TB mentioned. Please explain and correct this section as appropriate.

**Response: Sorry for the mistake we made. We grossly edited the paragraphs. Thank you so much for your constructive comment. We have removed all out mentioned narration from the main manuscript and you can look page 6 line 169**

**RESULTS**

**Comment 20**. Generally, the tables and figures give fairly comprehensive results relevant to describing the selected studies, and responding to the study objectives. The current narrative synthesis is very selective of bits and pieces from the tables and figures, and it does not do justice to the valuable information displayed in the tables and figures. The authors need to enhance the narrative synthesis of this systematic review and meta-analysis to provide a more comprehensive synthesis of the information in the tables and figures.

**Response: thanks for your information sharing as comments and I would like to share each it is the table figures are correctly narrated in descriptive report section detail please see once again Table1 and table2 and compared the descriptive reports part s please Thanks**

**Comment 21**. Study screening Please state the numbers identified, and screened out, AT ALL STEPS - separate results of screening by title, from screening by abstract. Revise the PRISMA flow diagram accordingly – with separate box for titles, then next screening box for abstracts. The arrows showing numbers of articles excluded between one level and the next are incorrectly located, they should be moved and placed between the relevant boxes.

**Response:-**Thank you for your comment and suggestion. We have carefully considered your feedback and made the necessary corrections. Here is the revised version of the PRSIAM, where we have clearly narrated the process of Study screening. We appreciate your input and hope that the changes address your concerns. **Please look here what we did**

**1st steps*;*** *We identify relevant databases like PubMed, Scopus, and Web of Science for searching potential studies using* ***PRISMA check lists*.**

**2^nd^ Steps we development of Search Strategy:**

A systematic search strategy is created to retrieve relevant studies from the identified databases. It includes keywords, Boolean operators, and MeSH terms (if applicable) for a comprehensive search. Initial Screening: Retrieved studies are imported into reference management software (e.g., EndNote) for efficient handling and duplicate removal. Two independent reviewers conduct an initial screening of titles and abstracts to exclude studies that clearly do not meet the inclusion criteria.

**3^rd^ Steps Full-Text Screening:** The remaining studies from the initial screening undergo a full-text assessment to determine their eligibility. Two independent reviewers carefully examine the full texts of the studies and assess them against predefined inclusion and exclusion criteria.

**Inclusion and Exclusion** Criteria: Inclusion and exclusion criteria are established based on research objectives and analysis scope. These criteria may include study design, population characteristics, intervention/exposure, outcome measures, and publication date.

**4^th^ Steps Resolution of Discrepancies:**

When we did this If discrepancies arise between the two independent reviewers during full-text screening, a third reviewer is consulted to reach a consensus. Discussions are held to resolve disagreements and ensure consistent application of the inclusion and exclusion criteria.

**5^th^ Steps Data Extraction:** finally we did this After final selection of studies, relevant data are extracted using a standardized form. It captures essential information such as study characteristics, participant demographics, intervention details, outcome measures, and key findings.

**Comment 22**. Two of the final 13 studies (refs 27 and 46) appear to have either the same study

population, or much overlap between the study populations. If this is true, the same study subjects are double-counted / duplicated throughout the synthesized results which is not statistically sound. Please verify if this is duplication of same study population or not. If they are different, they may be retained. If they are the same participants one study should be removed from the meta-analysis. [46] Firew Tiruneh tuberculosis-incidence among hiv-infected children on-haart-and theirclinical- profile-retrospective-cohort-study-south. Journal of AIDS & Clinical Research Research, Article Volume 11:3,2020, DOI: 1037421/jar202011808, 2020. [27] Deyas FTY. Effect of highly active antiretroviral treatment on TB incidence among HIV infected children and their clinical profile, retrospective cohort study, South West Ethiopia. Scientific Reports 10:21468, https://doiorg/101038/s41598-020-78466-0 2020.

**Response: We removed one study entitled Firew Tiruneh tuberculosis-incidence among HIV-infected children on-HAART-and their clinical- profile-retrospective-cohort-study-south. Journal of AIDS & Clinical Research, Article Volume 11:3,2020, DOI: 1037421/jar202011808, and 2020. Thank you for your comment and suggestions. Ale they are different studies in different area**

**Comment 23**. Descriptive characteristics of the studies. Table 1. These are well tabulated in table 1, but only partially described in the introductory paragraph.

• Please create sub-heading “Descriptive characteristics of the studies.” We did it

• Please explain what HT and HC refer to – and combined HT&HC described clearly in the methods section that covers factors of interest as it is analyzed.

**Response: We have made the necessary corrections based on your suggestion. Thank you very much for your constructive comments and suggestions. Regarding the narration, the study settings are classified into three categories. The first category includes study results based solely on health center data. The second category includes results from a hospital setup. The third category includes results from both health center and hospital setups. Data were collected accordingly, and subgroup analysis was performed based on each setting.**

**Comment 24**. Describing study setting: For global audience that may be unfamiliar with Ethiopia consider adding a supplemental figure map of Ethiopia that highlights where each study is located within Ethiopia. In narrative brief statement on where these identified regions are – e.g. mainly in central and southern regions of Ethiopia? The selected studies represent what proportion of the country’s regions?

**Response: Thank you for your suggestion.**

**Comment 25**. Table formatting comments:

• Please enhance column headings as follows: we have addressed the issue you raised

• state the unit for age (years?) we have narrated on given tables please refer it table 1

• Events – specify what event (No. children with TB?) We have stated please refer Table 1

**Results for study objectives**

**Response:** **We made appropriate change based on your suggestion. Thank you so much.**

**Comment 26**. Results on objective 1 is reported under a sub-heading “meta-analysis” which is confusing, since all the analyses reported in figures 2 to 6 are meta-analyses. Suggest you re-word the sub-headings using the actual language of the objective, as this makes results sections clear to reader. Suggested sub-headings for the remaining results:

**Comment 26.1**. **Pooled Prevalence of TB in Children with HIV on ART;**

In the final meta-analysis report, utilizing 13 published studies, we discovered that the estimated pooled burden of active TB among HIV-infected children in Ethiopia was 12.1% (95% CI: 10.7-13.4; I2 = 63.4%, P=0.001) (Fig 2).

**Comment 26.2** **Factors associated with TB in Children with HIV on ART**

In this systematic review, we analyzed adjusted odds ratios from primary studies to identify the risk factors for tuberculosis (TB) in HIV-infected children. We grouped statistically significant categorical variables from previous studies into themes, including WHO advanced clinical stages (III&IV), baseline CD4 count, missed isoniazid preventive therapy (IPT), missed cotrimoxazole preventive therapy (CPT), level of hemoglobin, antiretroviral therapy (ART) adherence status, and functional status of children. However, it is important to note that only missed IPT, missed CPT, WHO advanced clinical stages (III&IV) and level of hemoglobin were found to be predictors for active TB.

Accordingly, studies containing HIV infected children who missed IPT was double fold increase the odds of active TB occurrence compared with ever given children (OR: 2.28; 95% CI: 1.99-3.08). Moreover, the odds of TB co-infection for HIV-infected children for those who missed CPT were four time increased active TB infection compared with counter groups (OR: 4.26, 95% CI: 3.47--5.28). Likewise the likelihood of active TB occurrence in HIV infected children who are on advanced WHO clinical stage (III&IV) was 2.27 times (OR: 2.27; 95% CI:

1.81-2.73) higher than with children were on WHO clinical stage II and I. Finally, the probability of TB co-infection for HIV-infected children was 3.11 times higher (OR= 3.11, 95% CI: 1.57-4.7) for those cases had hemoglobin≤10 mg/dl compared to children with a hemoglobin level >10 mg/dl.

**Response: We made appropriate change based on your suggestion. Thank you so much.**

**Comment 27**. The majority (7/13= 53.8%) [1, 4, 23, 27, 29, 43, 47] of studies reported TB magnitudes, whereas the remaining four [26, 44, 48, 49] included crude incidence. The challenges of failure to clearly define the outcome of interest are reflected here.

PLEASE DECIDE WHAT YOUR OBJ. 1 OUTCOME MEASURE IS AND ADHERE TO THE SAME WORDING THROUGHOUT THE MANUSCRIPT. Magnitude needs to be quantified using a specific statistical measure such as Prevalence or proportion? Crude incidence vs incidence? Here 7 + 4 = 11 studies. Two studies omitted here, explain why they were omitted here.

“The final meta-analysis report indicated the pooled active TB prevalence among children after ART was estimated to be 12.1% (95% CI: 10.7 - 13.4; I2 = 63.4%, p=0.001) (Fig 1).” Wrong figure listed – I assume this statement reports on Figure 2.

**Response: Thank you so much for your a very important comment. We made appropriate change on the result section. Our primary outcome was to determine the pooled prevalence of TB among children on ART.**

**Comment 28**. I propose that you consider reporting separately the prevalence results for the 7 cross-sectional studies in a distinct forest plot. E.g. Prevalence of TB reported in seven cross-sectional studies ranged from …. (lowest prevalence) in study x, to …..(highest prevalence) in study z. Then the meta-analyses pooled prevalence for the 7 cross-sectional studies. Then report on the 9 cohort study findings – if they provided both proportions of children with TB (surrogate for prevalence?), as well as incidence of TB – these may be reported in separate forest plots.

**Response: We made change on the results. Thank you.**

**Comment 29**. There is need to report separately the results for TB incidence across those nine cohort studies that reported on it. It would be useful to report the pooled incidence and provide a separate forest plot for this value. Objective 2 results: Factors associated with TB. Figures 3 to 6 generally very nice sub-group analyses comparing various sub-groups at geographical level, study setting, and patient-specific risk factors.

**Response: Thanks for your nice comments, we have amended and It is a similar comment with the above comments. We made change on it and corrected based on the suggested one .**

**Here is what we did Factors associated with active TB in HIV infected children on ART**

In our subgroup analysis, we examined TB pooled prevalence among HIV infected children based on the study setting (Hospital set up and Health center and both). Accordingly, the studies result indicated, pooled prevalence was slightly lower at 11.05% (95%CI: 9.4-12.3) in hospital set up than health center studies reports 14.1% (95%CI: 11.74-16.33) **(Fig 3).** Pooled TB burden among HIV infected children was significantly higher for studies conducted in the Oromia region at 15.6% (95%CI: 10.2-20.6) compared with studies included from SNNR results of 12.8% (95%CI: 10.03-15.67)**.**

In this report, the duration of follow-up periods was found to be significantly associated with the occurrence of active TB. Sub-group analysis revealed that the pooled burden of TB among HIV-infected children with a follow-up period of ≤10 years was significantly higher at 13.67% (95%CI: 11.24-15.1) compared to those with a follow-up period of >10 years, which had estimation of 10.9% (95%CI: 9.1-12.8) **(Fig 4).** .

In this systematic review, to identify factors associated with active TB we analyzed adjusted odds ratios from primary studies to identify the risk factors for tuberculosis (TB) in HIV-infected children. We grouped statistically significant categorical variables from previous studies into themes, including WHO advanced clinical stages (III&IV), baseline CD4 count, missed isoniazid preventive therapy (IPT), missed cotrimoxazole preventive therapy (CPT), level of hemoglobin, antiretroviral therapy (ART) adherence status, and functional status of children. However, it is important to note that only missed IPT, missed CPT, WHO advanced clinical stages (III&IV) and level of hemoglobin were found to be predictors for active TB **(Table 2)**.

Accordingly, studies containing HIV infected children who missed IPT was double fold increase the odds of active TB occurrence compared with ever given children (OR: 2.28; 95% CI: 1.99-3.08). Moreover, the odds of TB co-infection for HIV-infected children for those who missed CPT were four time increased active TB infection compared with counter groups (OR: 4.26, 95% CI: 3.47--5.28). Likewise the likelihood of active TB occurrence in HIV infected children who are on advanced WHO clinical stage (III&IV) was 2.27 times (OR: 2.27; 95% CI: 1.81-2.73) higher than with children were on WHO clinical stage II and I. Finally, the probability of TB co-infection for HIV-infected children was 3.11 times higher (OR= 3.11, 95% CI: 1.57-4.7) for cases having hemoglobin≤10 mg/dl compared to children with a Hgb >10 mg/dl (**Fig 5).**

**Comment 30**. Two headings “sub-group analyses” and “risk factors analyses” not necessary. Both sections cover objective 2, and both involve sub-group analyses – so the different headings are confusing. Please merge the two sections and use one heading e.g. “Factors associated with TB in children with HIV on ART”

**Response: We made change on it and please referee it**

**Comment 31**. There is inadequate detail in explaining clearly some of the sub-group variables, and incomplete narrative for each forest plot. The researchers are encouraged to ensure the following: That each sub-group is clearly defined where appropriate (e.g. HT vs HT&HC, but plot reports facility and hospital,

**Response: Thank you. We rewrote the section on the given Figure particular we made re-arranged, re- depicted the graph of subgroup- analysis by categorizing only in two sections that is studies from Health center, and Hospital set up only and sorry for mistakenly written.** IPT no definition is there a non-IPT population, and how are they defined) and That the risk group and reference group to which it is compared is clearly stated (e.g. IPT and cotrimoxazole meta-analyses, it is unclear who is compared to whom)

**Response** thanks reviewer for your comments. Regarding the blurring of your viewing for narration of IPT / CPT in the our result , we selected articles based on their published article categories. If patient ever IPT /CPT were given during follow-up, it was labeled as "yes." If they missed IPT/CPT or not given was considered as not given. We analyzed the cases where patients did not take the treatment (one group) and controls were those who received or took it. This is a summary of what we did. I understand that you may have concerns, but in the field of science, it is clear and cases are not given children and control is who missed IPT/CPT

**Comment 32**. “In our subgroup analysis, TB prevalence among HIV-positive children in studies in Hospital setup was slightly lower than facility-based studies at 11.05% (95%CI: 9.4- 12.3 Vs. 14.1%; 95%CI: 11.74- 16.33).” A new term appears “facility-based” that was not described in table 1. For studies in HT versus studies in HT&HC settings was data of those children reported in HC only separated to provide this result on “facility-based” sub-group? Please clarify. If so – a supplemental data table that stratifies data (numerator – events, and denominator number of children) for the studies that enrolled children from both HT and HC settings would be helpful, and would give confidence of validity of this finding.

**Response: We made appropriate change on it. Still you nagged on similar question and Thank you so much please we have responded it .**

**Comment 33**. There is no forest plot for the comparison of regions, but there is narrative reporting on the same. Please provide the forest plot.

**Response: Thank you so much for your comment. Figure 3 states about subgroup analysis based on setting regions.**

**Comment 34**. Comments on formatting and legends of the figures:

• If possible, add a column of the actual numbers for sample size of each study within the plot against each study, and the pooled sample size.

• In the legend, after the legend title add more information as appropriate. E.g. fig 5 and fig 6. State selection criteria for this sub-set of children that are analyzed in the plots.

**Response: we have amended please accepted it and proceeded the next steps for publication**

**Comment 35**. **Discussion**

Once the suggested improvements are done in the analysis and presentation of results – especially separation of prevalence from incidence results, the discussion should be revised accordingly.

**Response: We revised the discussion section thoroughly. Thank you for your comment.**

**Comment 36**. **Conclusions**

May be refined based on comments given for study abstract concluding statement, and enhanced to incorporate conclusions on pooled incidence as well as pooled prevalence.

**Response:** We rewrote the conclusion section based on the revision. Here is what seems to be the conclusion of the final report “This systematic review found a higher prevalence of active TB in HIV-infected children in Ethiopia compared to the estimated rates in the end TB strategy. Risk factors for active TB were identified included WHO clinical stages IV and III, low hemoglobin, missed IPT, and missed CPT were predictors. To reduce the risk of active TB, it is crucial to implement effective strategies such as regular IPT mission and addressing the gaps in treatment, and routine screening for active TB during follow-ups to prevent premature death.”

**Reviewer 3**

**Comment** a) Review the spelling errors as indicated in the attachment in the previous and current review. eg Whorl Health Organization for World Health Organization. Human Immune Deficiency Varies for Human Immunodeficiency Virus Forest Plotted for Forest Plot

**Response: We have made change based on your suggestions. We have also seen the whole document thoroughly and we made corrections for spelling and Grammer errors. Thank you so much for your suggestion.**

**Comment** b) Gross errors like use of Hgb <10mg/dl instead of 10gm/dl in the paper are serious oversights by the team of authors! Please review and rectify.

**Response: We made change from mg to gm. Thank you so much for your comment.**

**Comment** c) Results to be provided as absolute facts based on your findings, avoid use of terminologies like "more than half", "most" and "Almost all".

**Response: We used appropriate words instead of the terminologies mentioned above. Thank you so much for your suggestion.**

**Comment** d) Conclusions require adequate referencing to prevent it to seem like arbitrary statements and opinions. Here the corrected version of conclusion This systematic review found a higher prevalence of active TB in HIV-infected children in Ethiopia compared to the estimated rates in the end TB strategy. Risk factors for active TB were identified included WHO clinical stages IV and III, low hemoglobin, missed IPT, and missed CPT were predictors. To reduce the risk of active TB, it is crucial to implement effective strategies such as regular IPT mission and addressing the gaps in treatment, and routine screening for active TB during follow-ups to prevent premature death.

**Comment** e) Title and Short title require work to be done - Title speaks of incidence of TB, however in your methodology there is use of prevalence. Title is not clear and concise and short title is incomplete.

**Response: We made appropriate change on the tittle and short tittle. Throughout the document, we used pooled prevalence instead of prevalence consistently. Thank you again for your constructive comment.**

**Comment** f) Few of the results and inferences like CPT do not have p values provided for

**Response: We made appropriate corrections on the mother document.**

g) Rephrasing of the results and inferences. There is repetition between CPT and IPT.

**Response: We made appropriate corrections on the mother document.**
